# Supplementary figures and images for: Thermal insulation of poly(methyl methacrylate) bone cement and hydroxyapatite coatings under induction heating of metal implants (part 1 of 3)
Source: PLoS One. 2025 Dec 11;20(12):e0338325. doi: 10.1371/journal.pone.0338325 (PMC12698007; doi:10.1371/journal.pone.0338325)

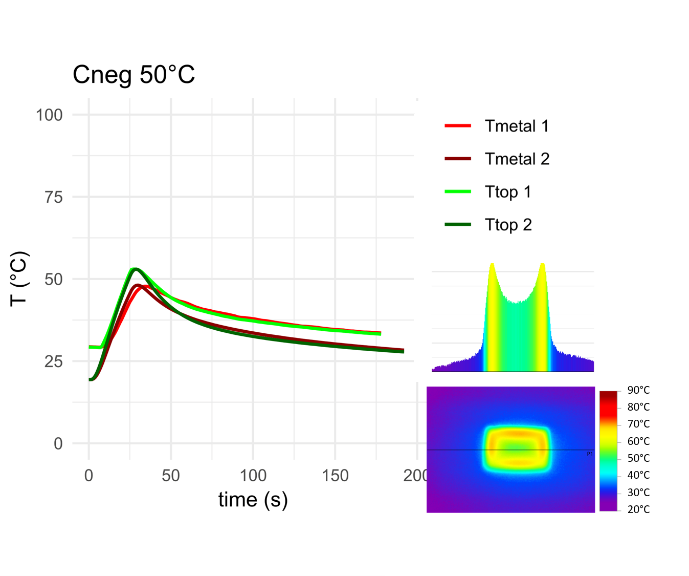

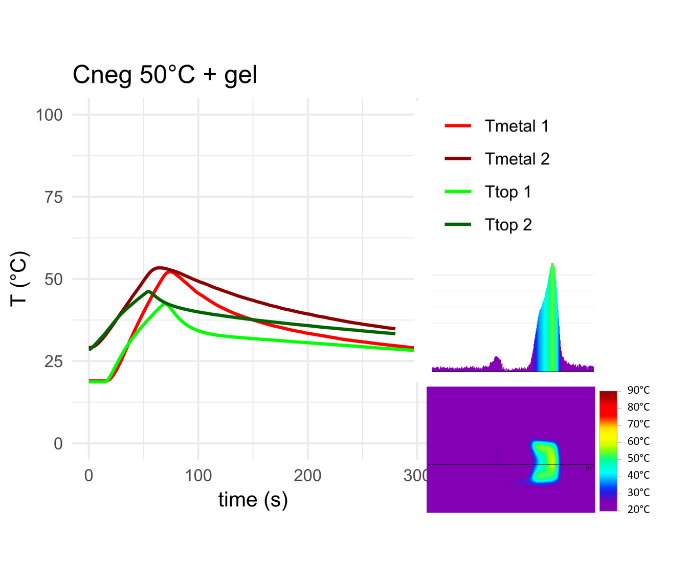

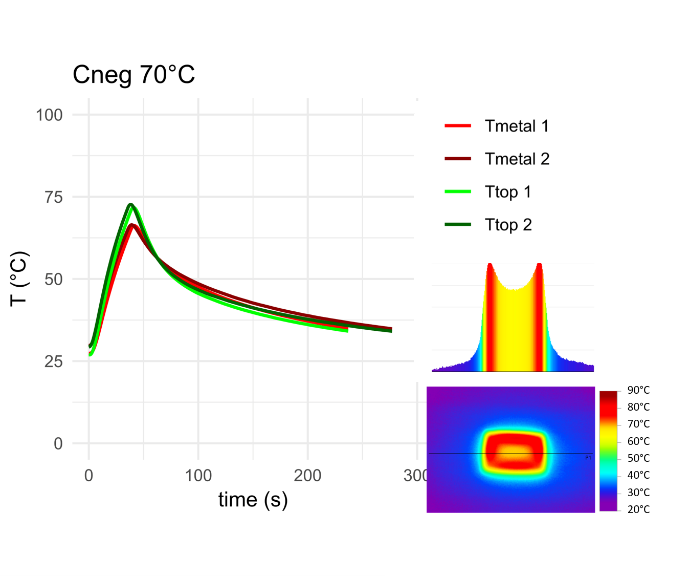

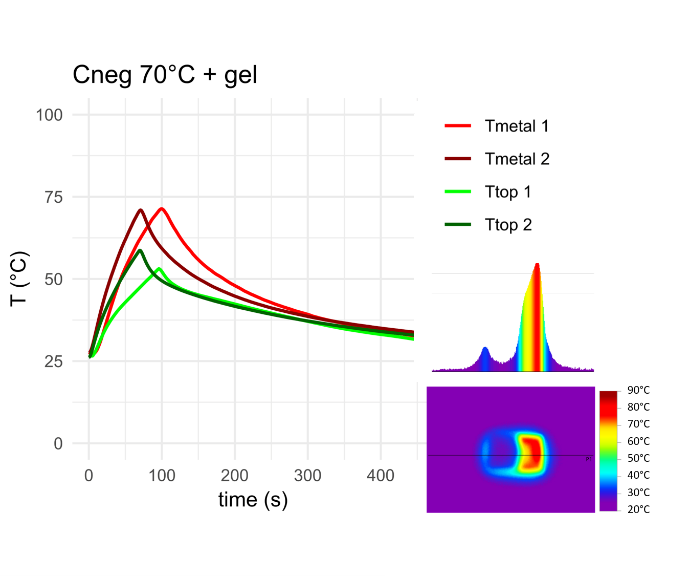

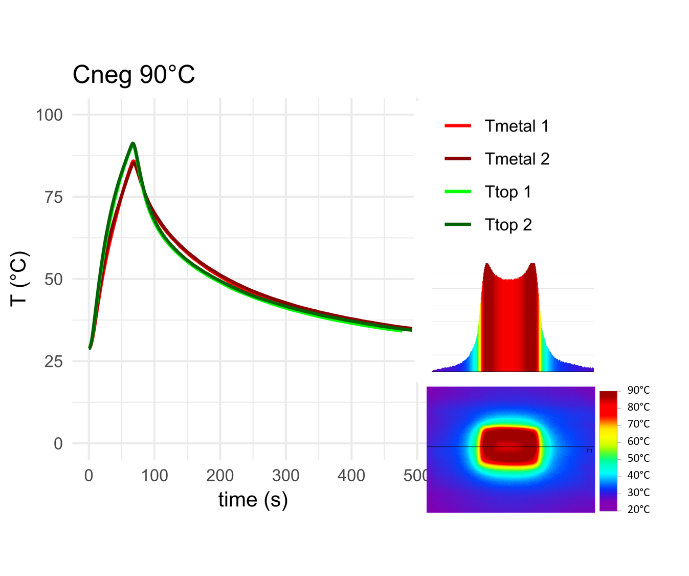

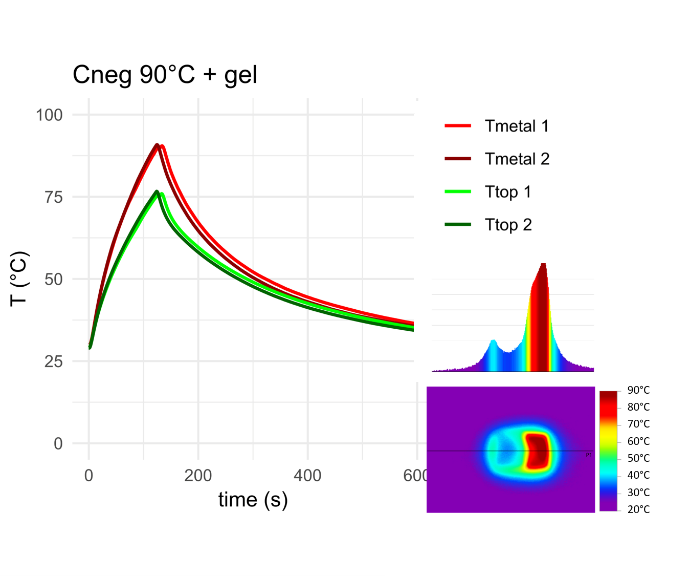


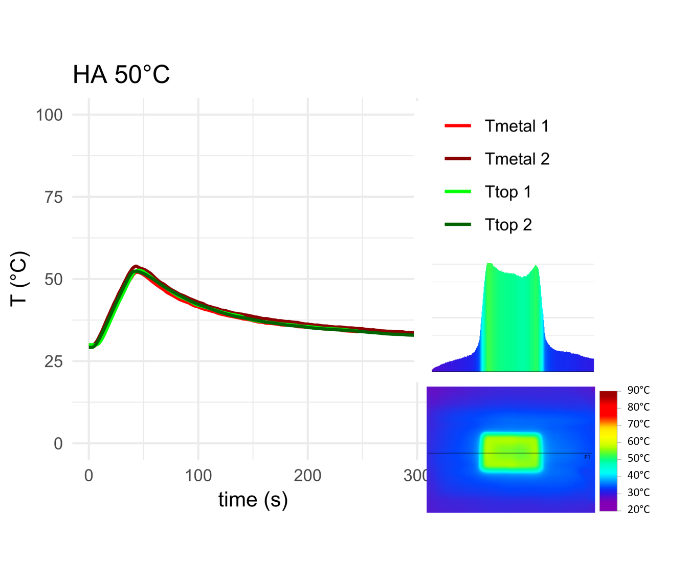

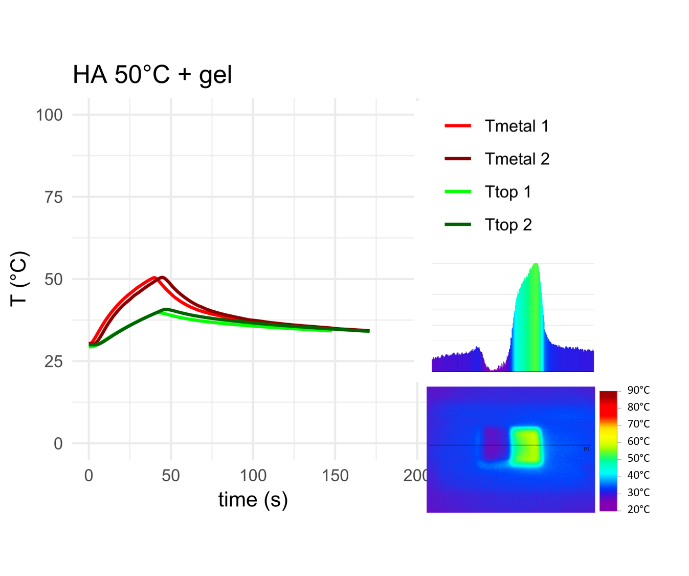

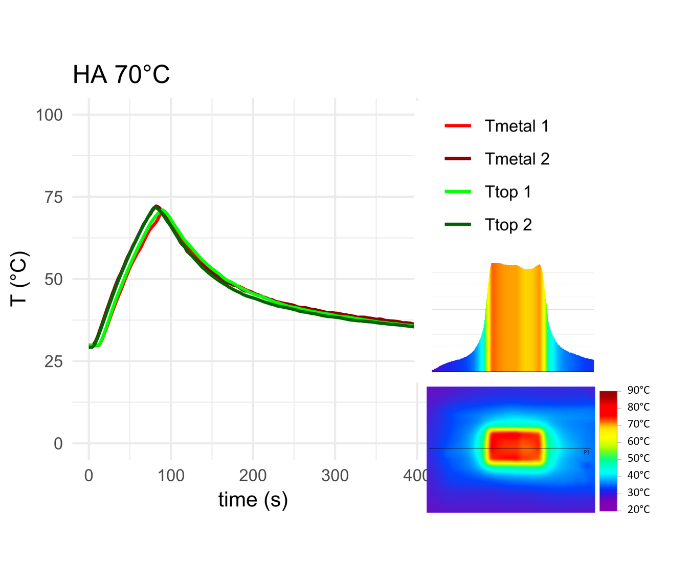

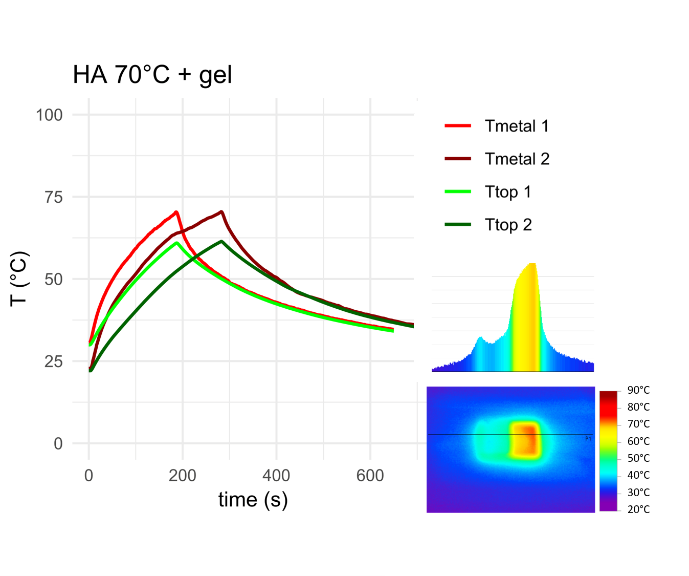

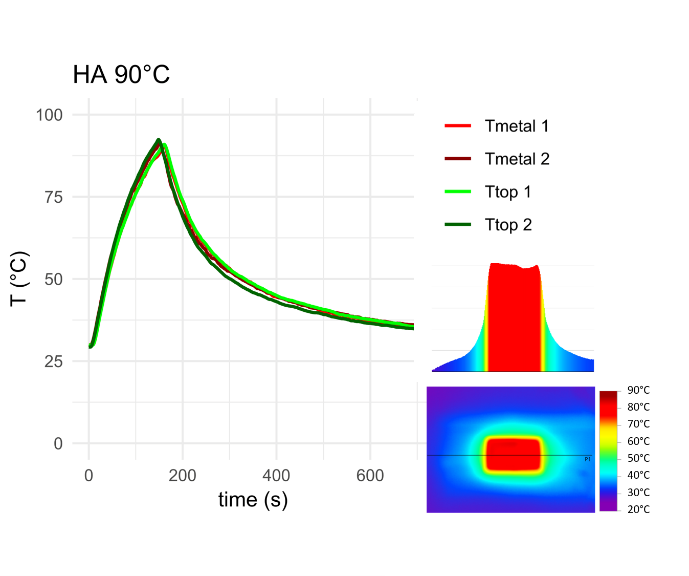

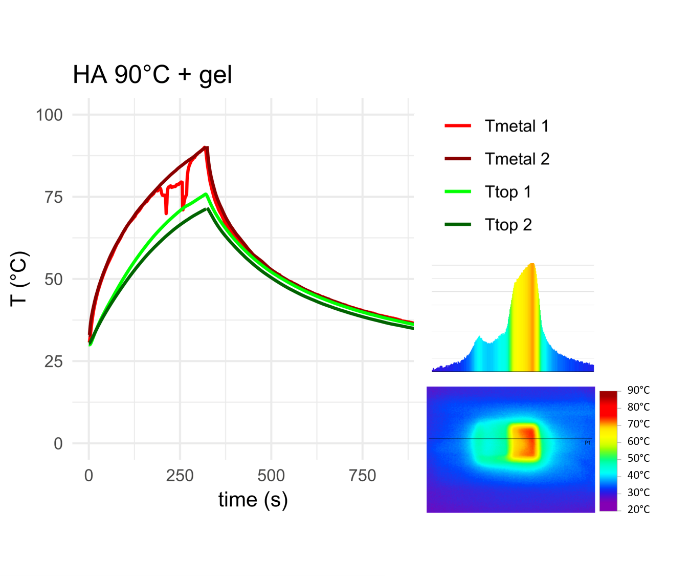


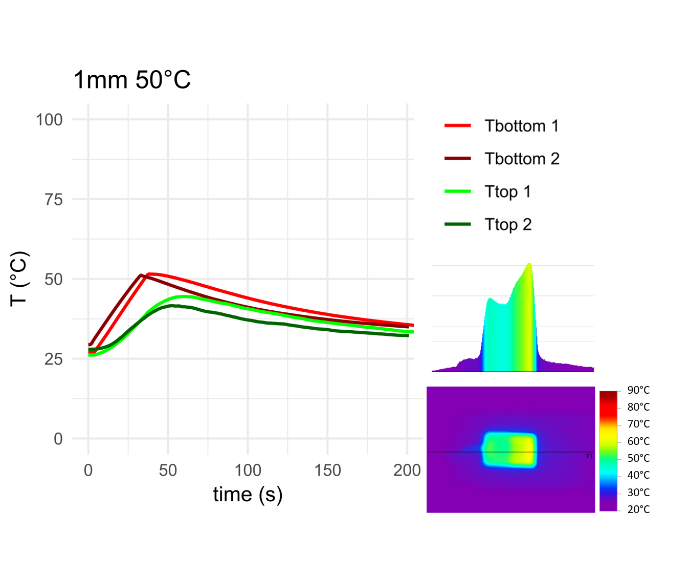

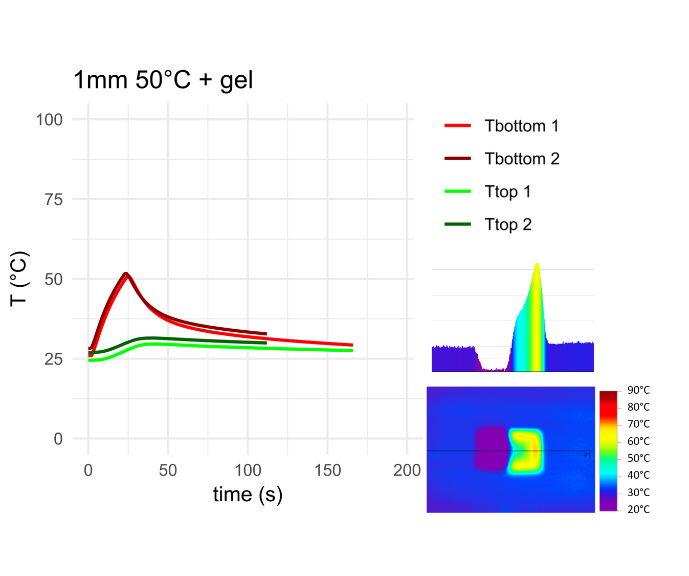

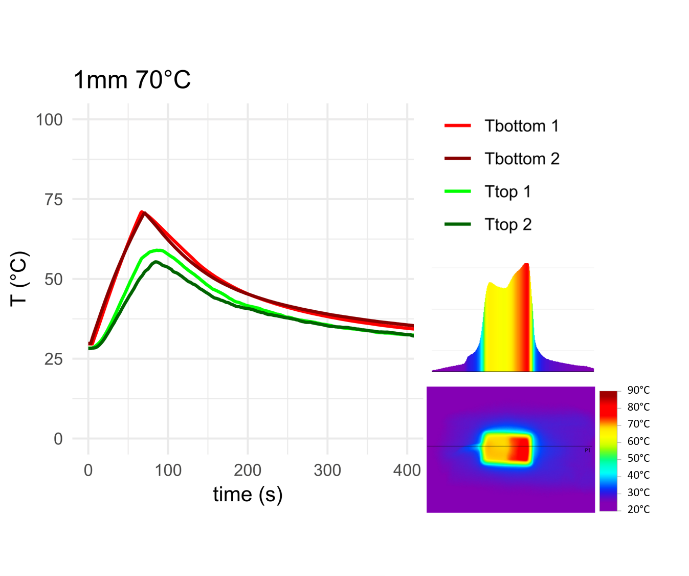

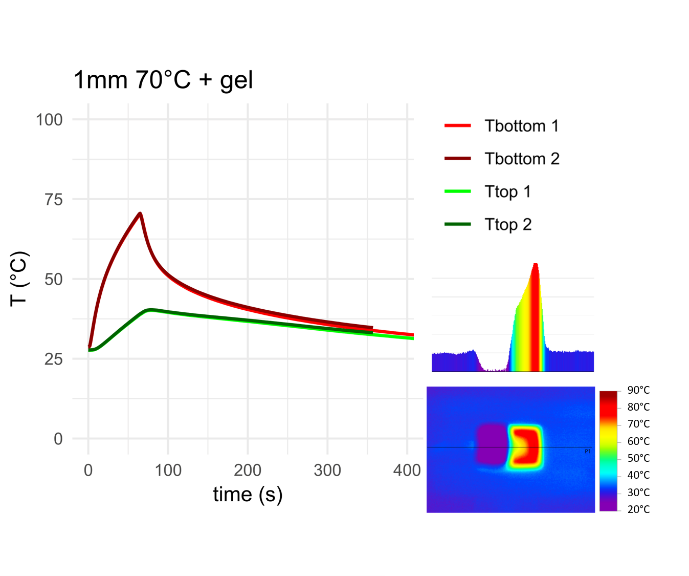

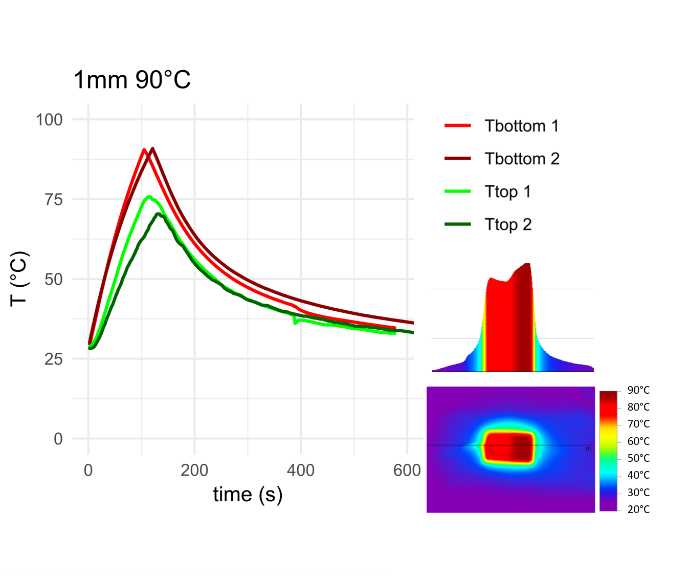

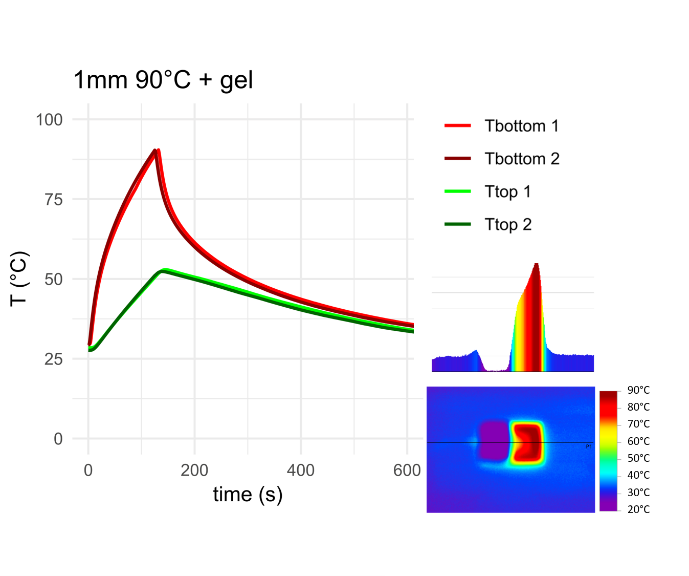

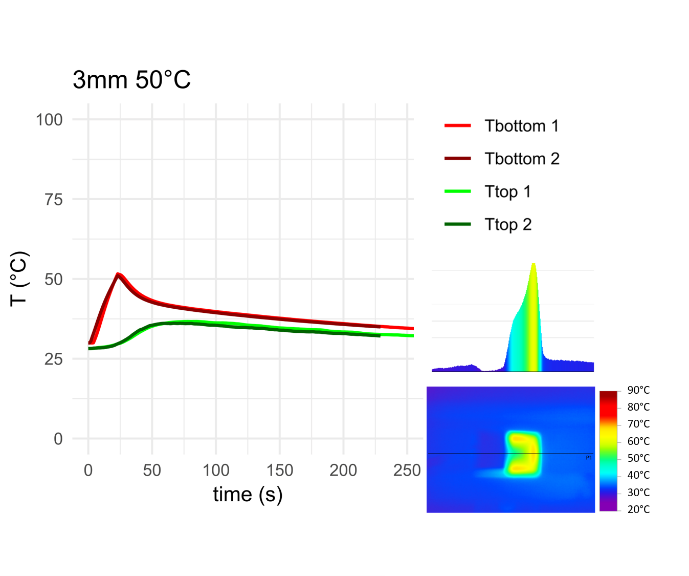

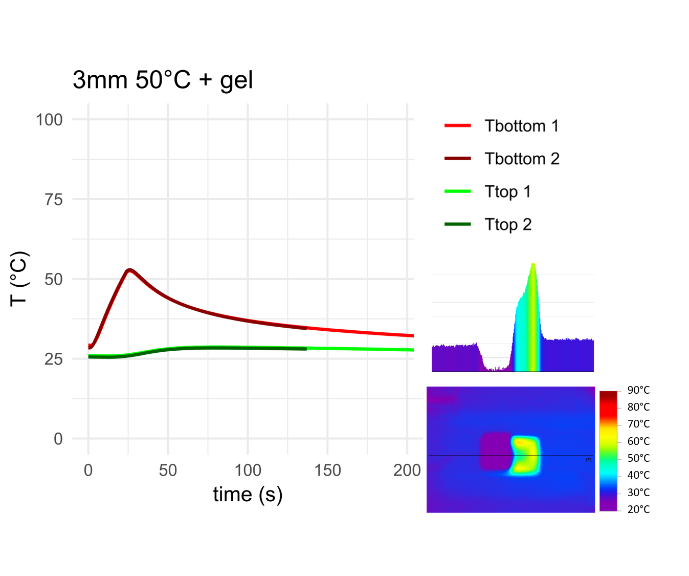

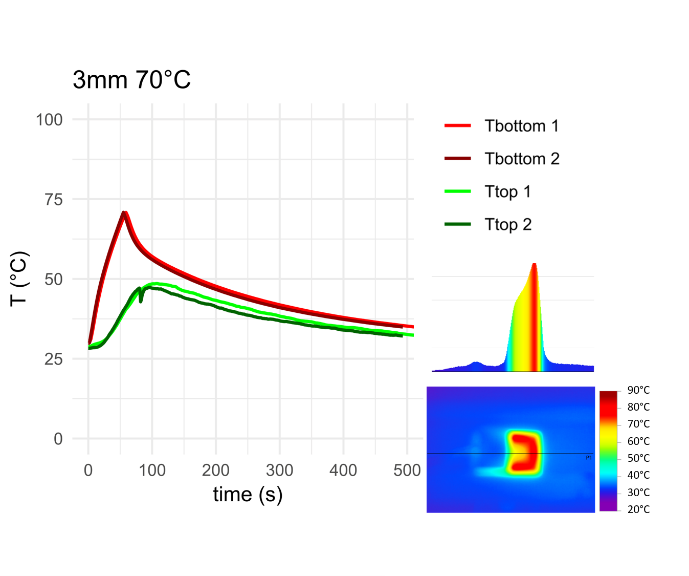

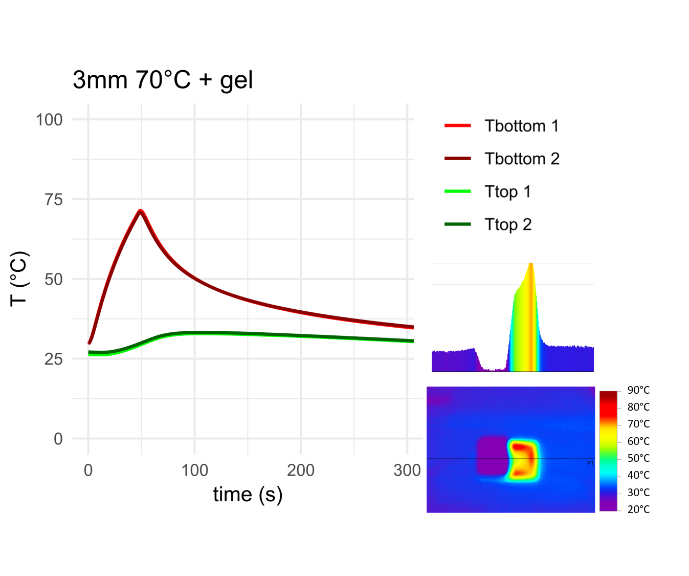

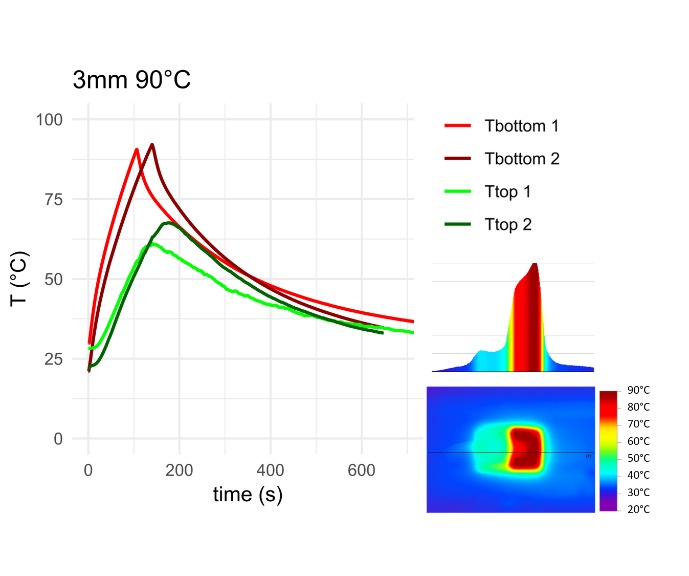

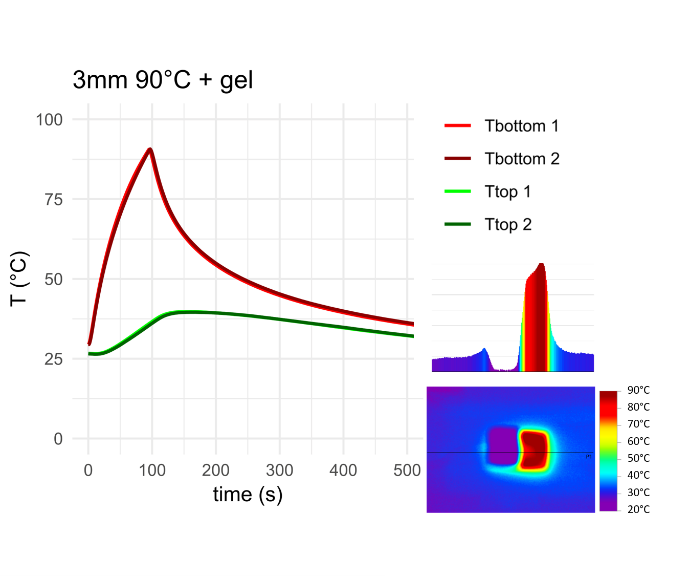

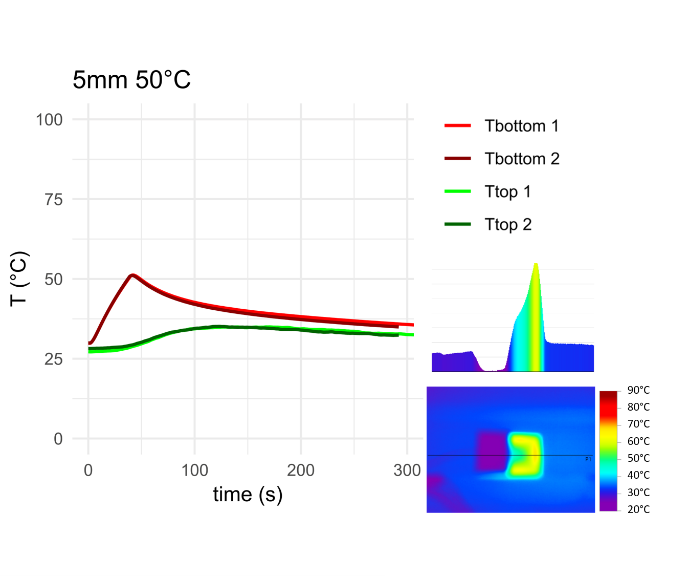

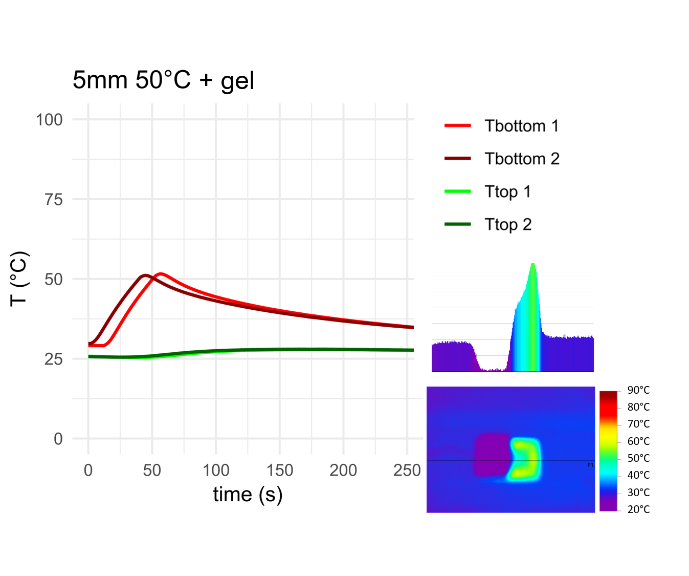

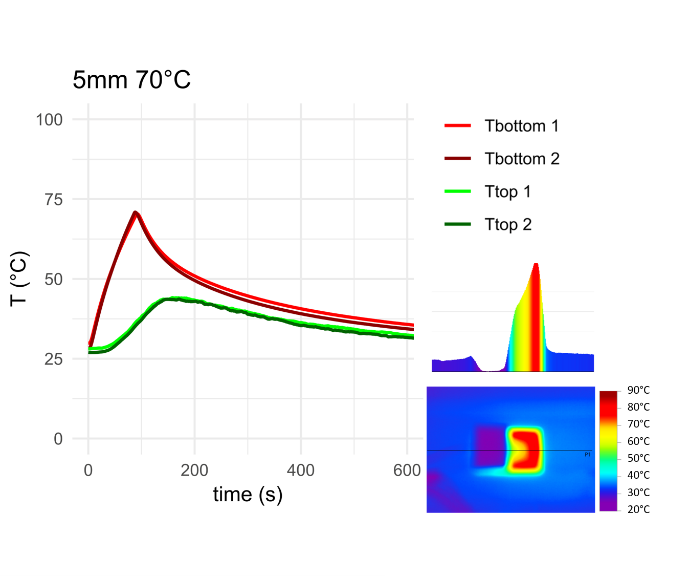

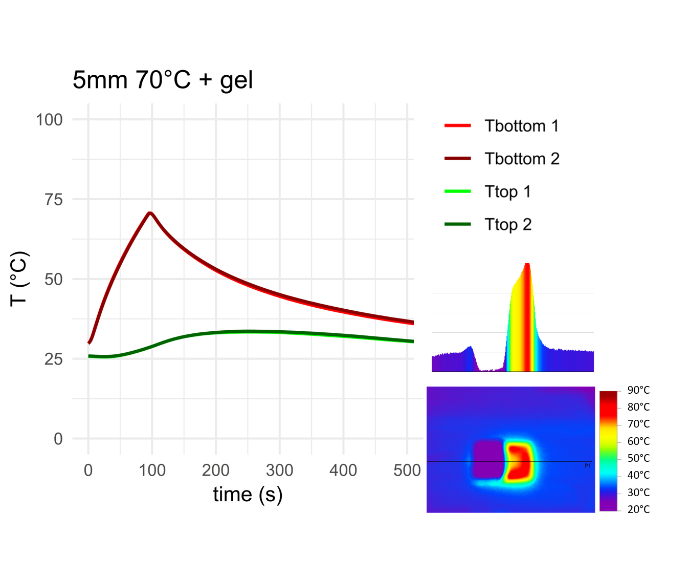

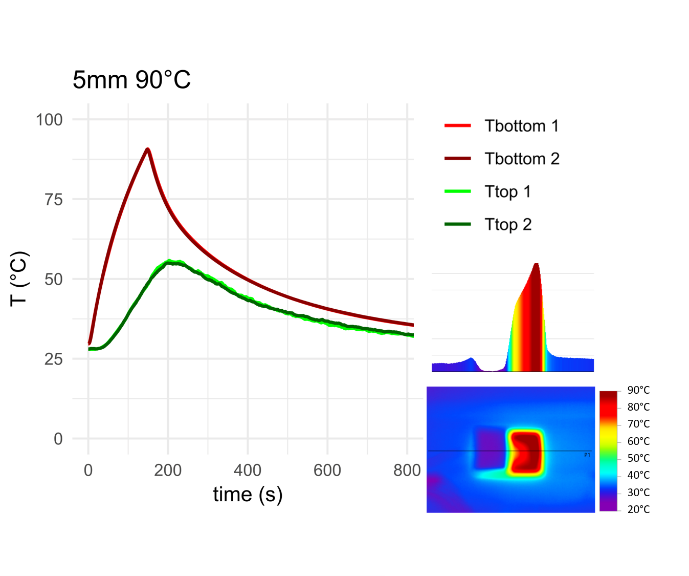

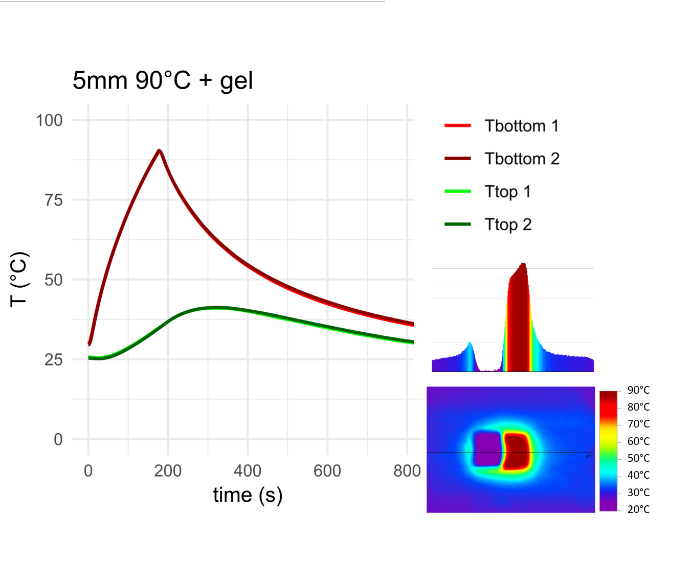

Supplement: S1 Figs — (DOCX) [file pone.0338325.s001.docx]

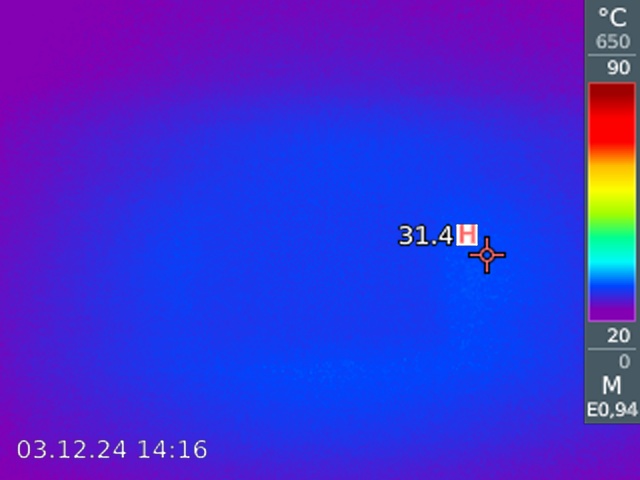

Supplement: S2 Figs — (ZIP) [file pone.0338325.s002.zip › image series/0. Cneg/TR004687.JPG]

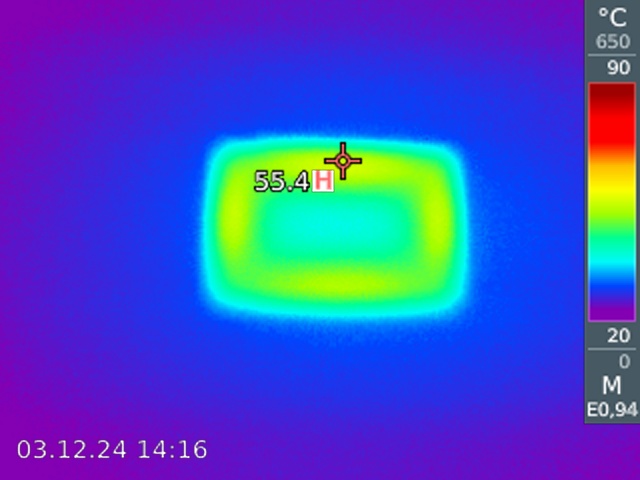

Supplement: S2 Figs — (ZIP) [file pone.0338325.s002.zip › image series/0. Cneg/TR004688.JPG]

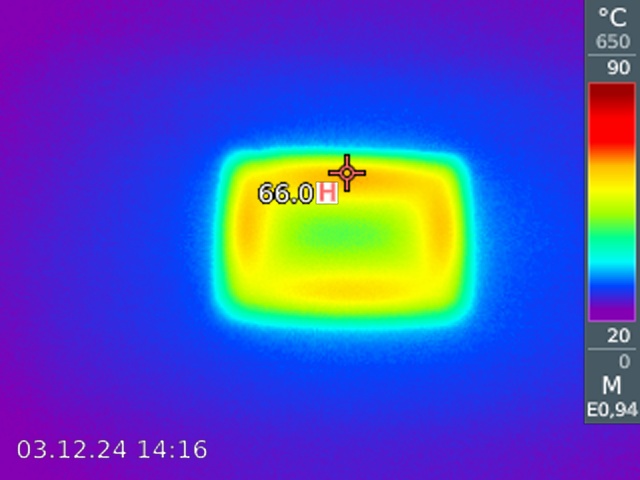

Supplement: S2 Figs — (ZIP) [file pone.0338325.s002.zip › image series/0. Cneg/TR004689.JPG]

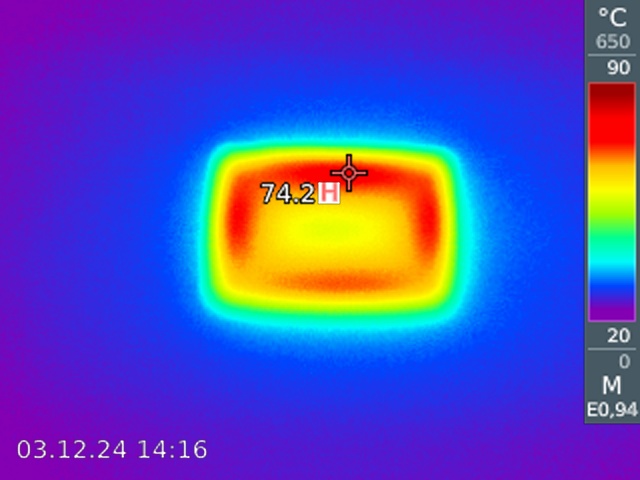

Supplement: S2 Figs — (ZIP) [file pone.0338325.s002.zip › image series/0. Cneg/TR004690.JPG]

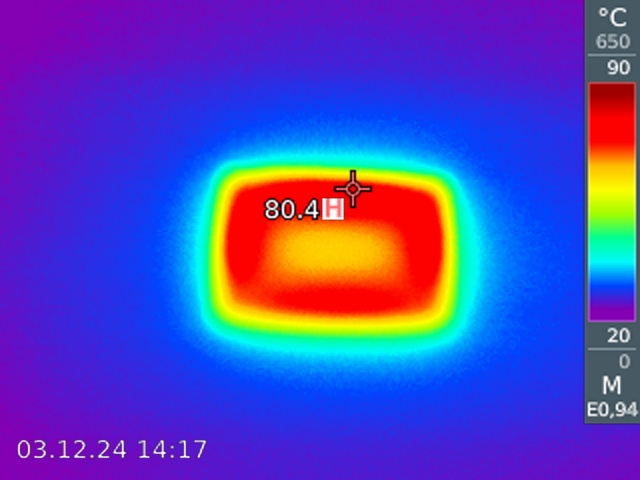

Supplement: S2 Figs — (ZIP) [file pone.0338325.s002.zip › image series/0. Cneg/TR004691.JPG]

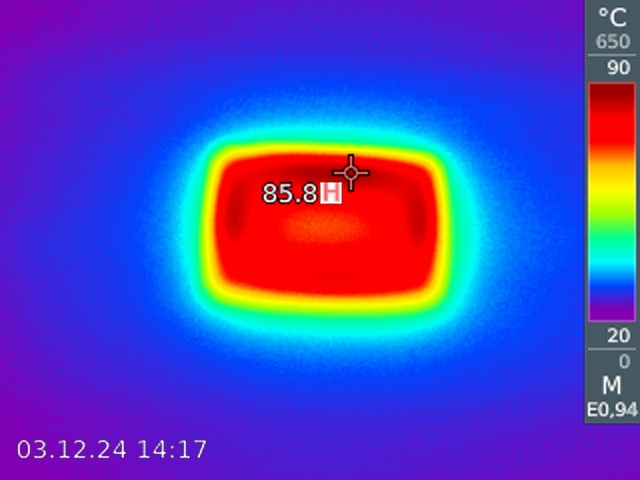

Supplement: S2 Figs — (ZIP) [file pone.0338325.s002.zip › image series/0. Cneg/TR004692.JPG]

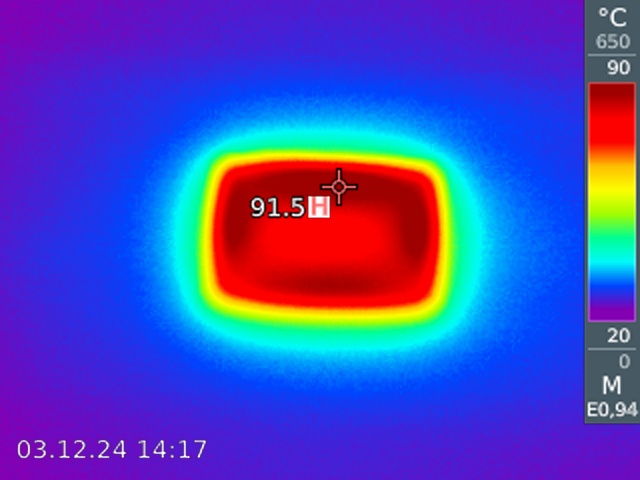

Supplement: S2 Figs — (ZIP) [file pone.0338325.s002.zip › image series/0. Cneg/TR004693.JPG]

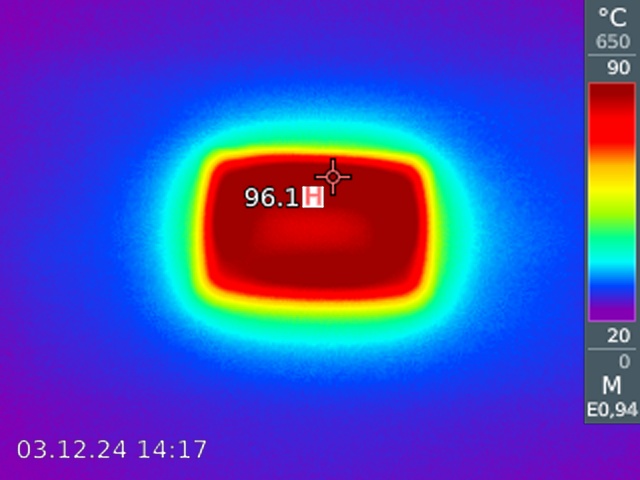

Supplement: S2 Figs — (ZIP) [file pone.0338325.s002.zip › image series/0. Cneg/TR004694.JPG]

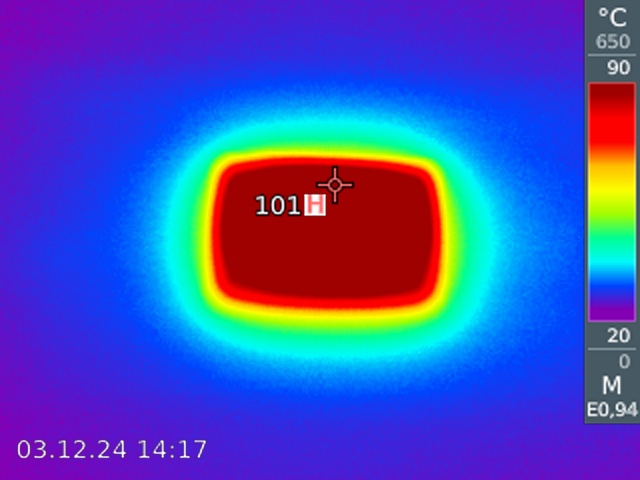

Supplement: S2 Figs — (ZIP) [file pone.0338325.s002.zip › image series/0. Cneg/TR004695.JPG]

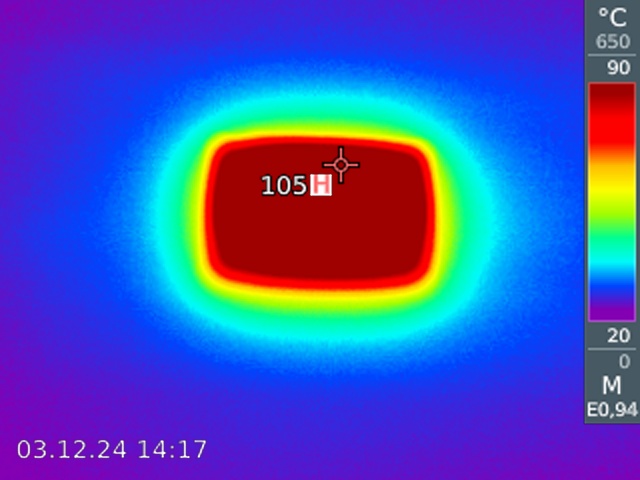

Supplement: S2 Figs — (ZIP) [file pone.0338325.s002.zip › image series/0. Cneg/TR004696.JPG]

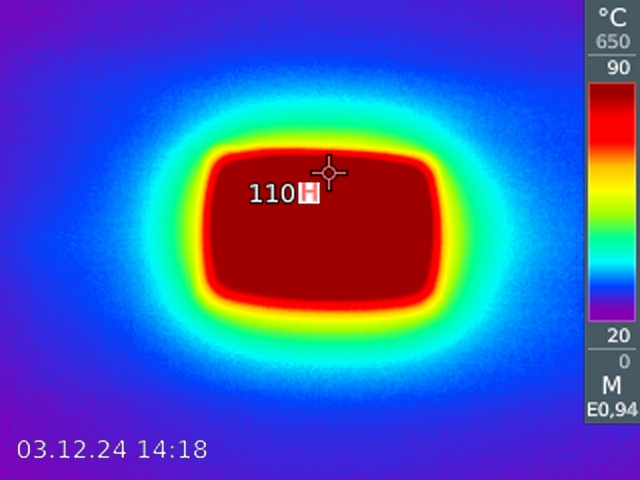

Supplement: S2 Figs — (ZIP) [file pone.0338325.s002.zip › image series/0. Cneg/TR004697.JPG]

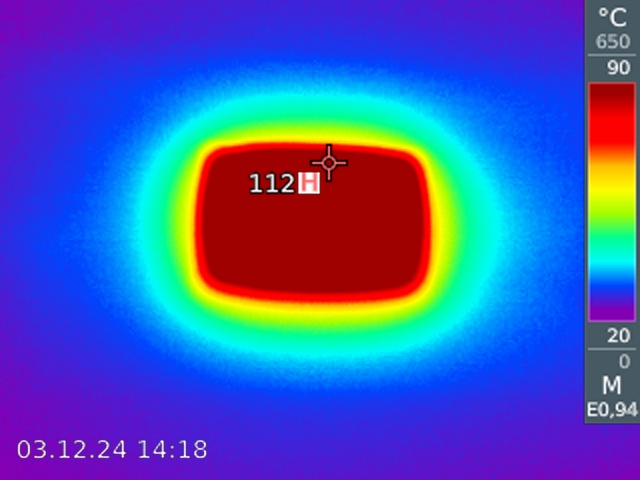

Supplement: S2 Figs — (ZIP) [file pone.0338325.s002.zip › image series/0. Cneg/TR004698.JPG]

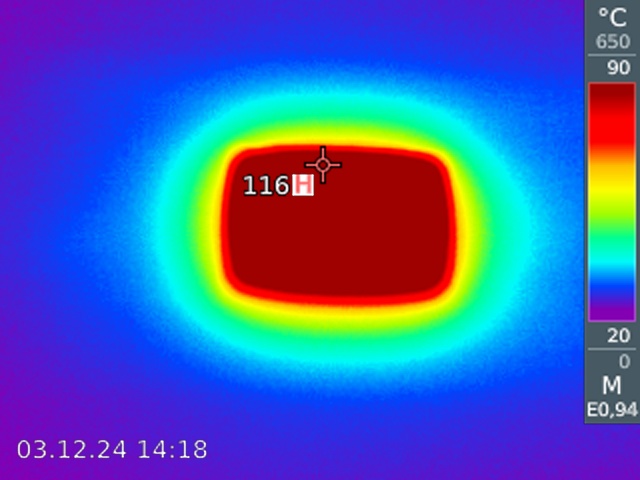

Supplement: S2 Figs — (ZIP) [file pone.0338325.s002.zip › image series/0. Cneg/TR004699.JPG]

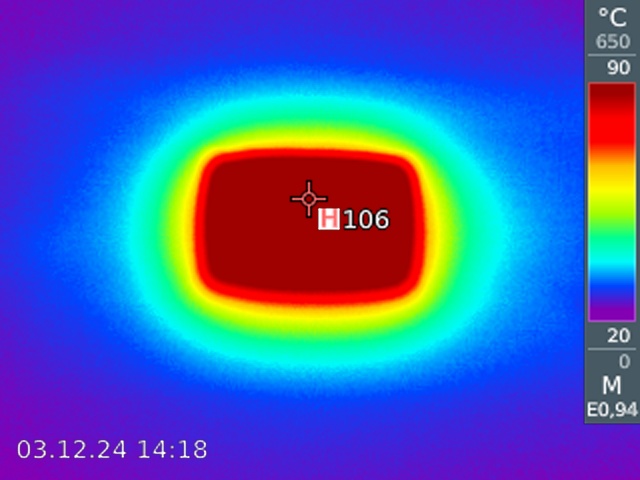

Supplement: S2 Figs — (ZIP) [file pone.0338325.s002.zip › image series/0. Cneg/TR004700.JPG]

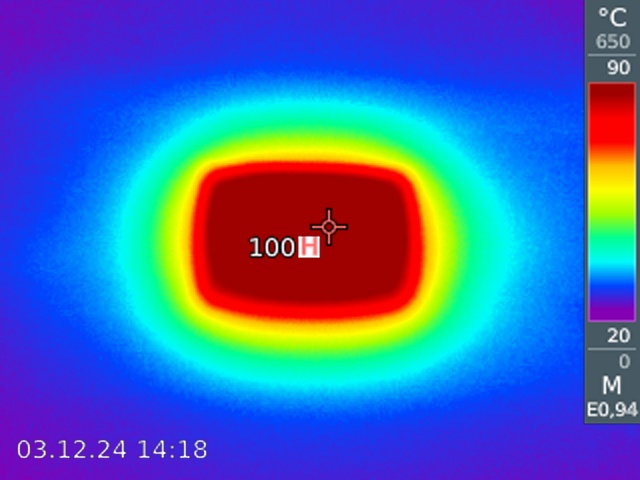

Supplement: S2 Figs — (ZIP) [file pone.0338325.s002.zip › image series/0. Cneg/TR004701.JPG]

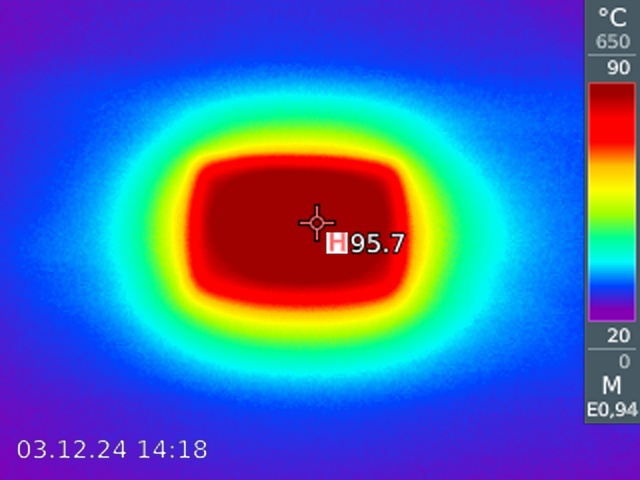

Supplement: S2 Figs — (ZIP) [file pone.0338325.s002.zip › image series/0. Cneg/TR004702.JPG]

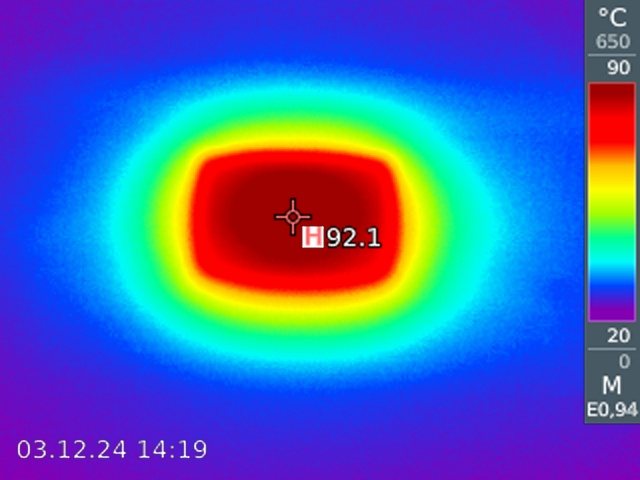

Supplement: S2 Figs — (ZIP) [file pone.0338325.s002.zip › image series/0. Cneg/TR004703.JPG]

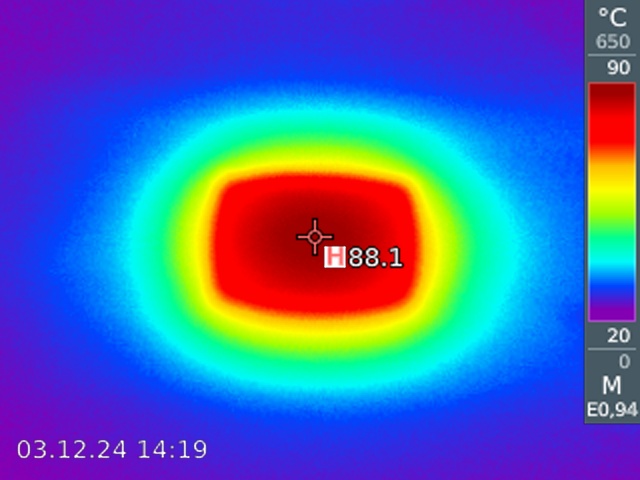

Supplement: S2 Figs — (ZIP) [file pone.0338325.s002.zip › image series/0. Cneg/TR004704.JPG]

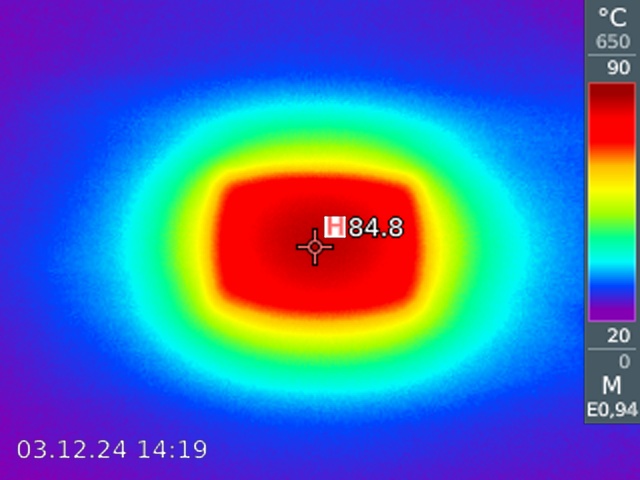

Supplement: S2 Figs — (ZIP) [file pone.0338325.s002.zip › image series/0. Cneg/TR004705.JPG]

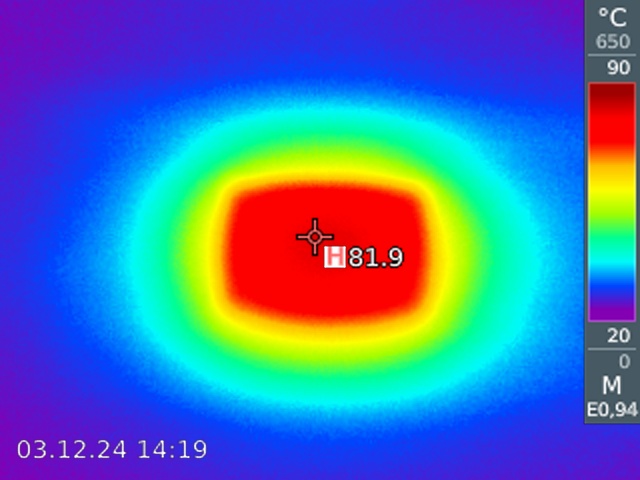

Supplement: S2 Figs — (ZIP) [file pone.0338325.s002.zip › image series/0. Cneg/TR004706.JPG]

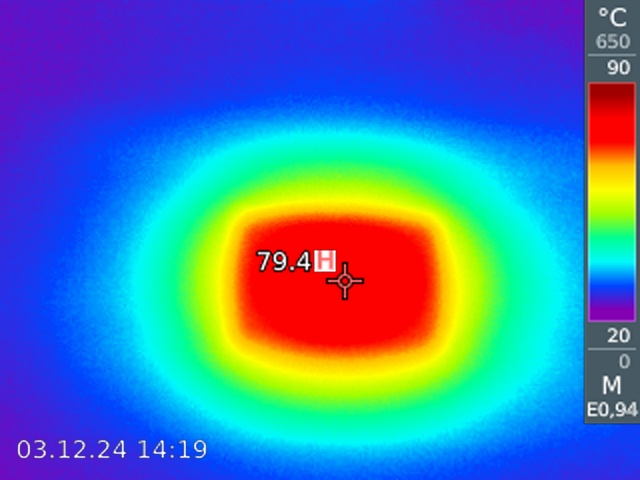

Supplement: S2 Figs — (ZIP) [file pone.0338325.s002.zip › image series/0. Cneg/TR004707.JPG]

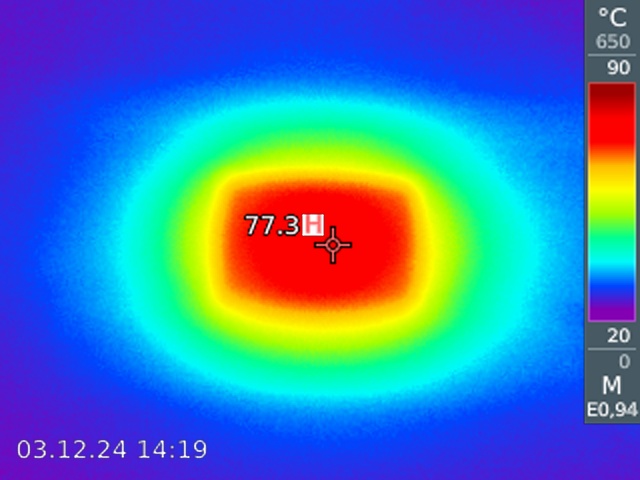

Supplement: S2 Figs — (ZIP) [file pone.0338325.s002.zip › image series/0. Cneg/TR004708.JPG]

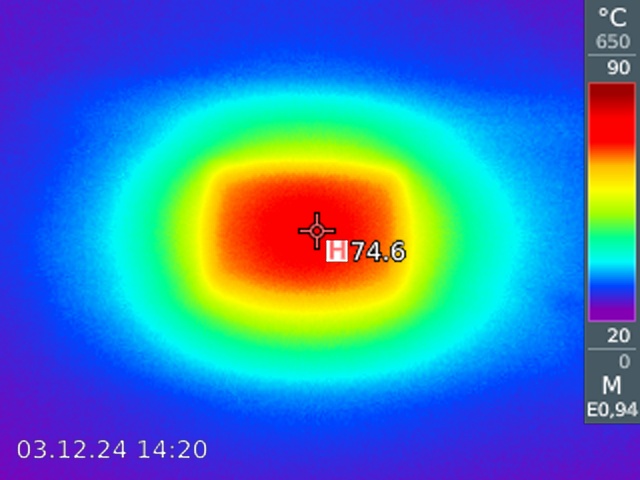

Supplement: S2 Figs — (ZIP) [file pone.0338325.s002.zip › image series/0. Cneg/TR004709.JPG]

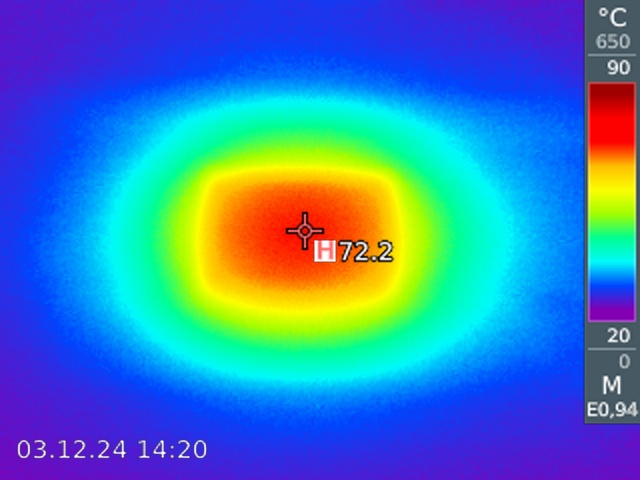

Supplement: S2 Figs — (ZIP) [file pone.0338325.s002.zip › image series/0. Cneg/TR004710.JPG]

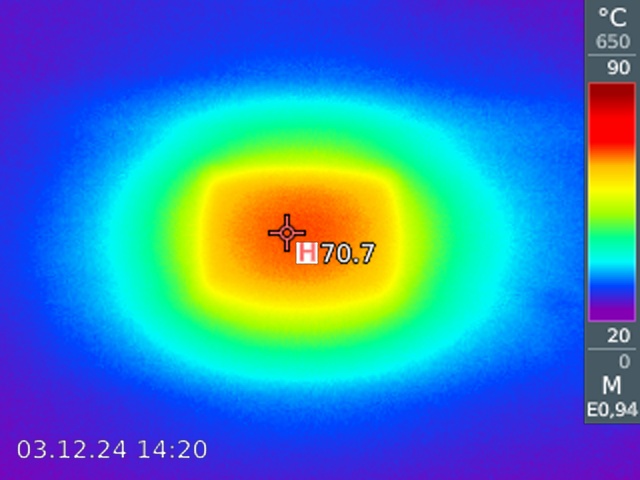

Supplement: S2 Figs — (ZIP) [file pone.0338325.s002.zip › image series/0. Cneg/TR004711.JPG]

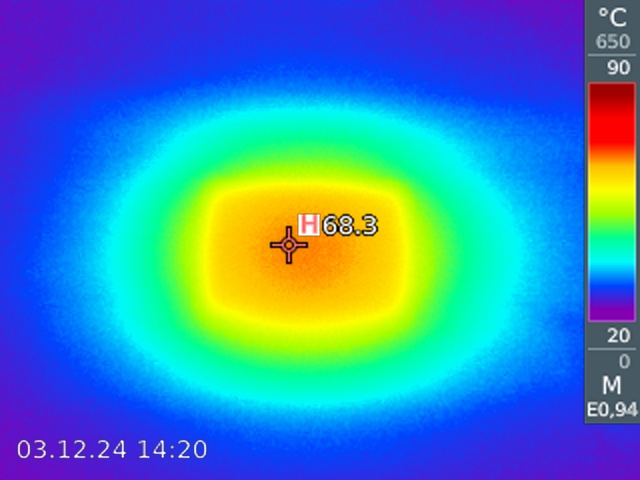

Supplement: S2 Figs — (ZIP) [file pone.0338325.s002.zip › image series/0. Cneg/TR004712.JPG]

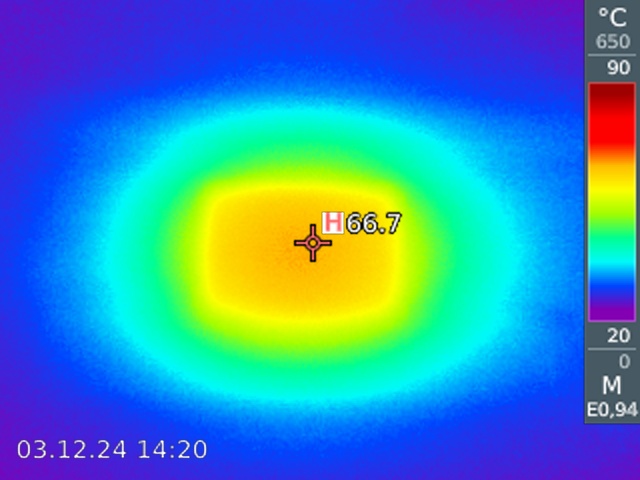

Supplement: S2 Figs — (ZIP) [file pone.0338325.s002.zip › image series/0. Cneg/TR004713.JPG]

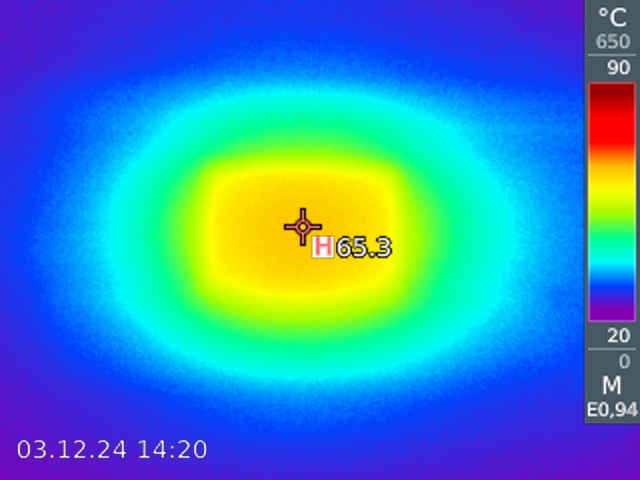

Supplement: S2 Figs — (ZIP) [file pone.0338325.s002.zip › image series/0. Cneg/TR004714.JPG]

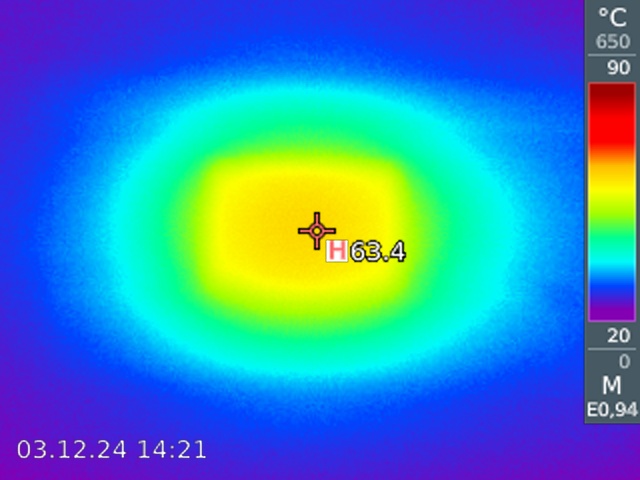

Supplement: S2 Figs — (ZIP) [file pone.0338325.s002.zip › image series/0. Cneg/TR004715.JPG]

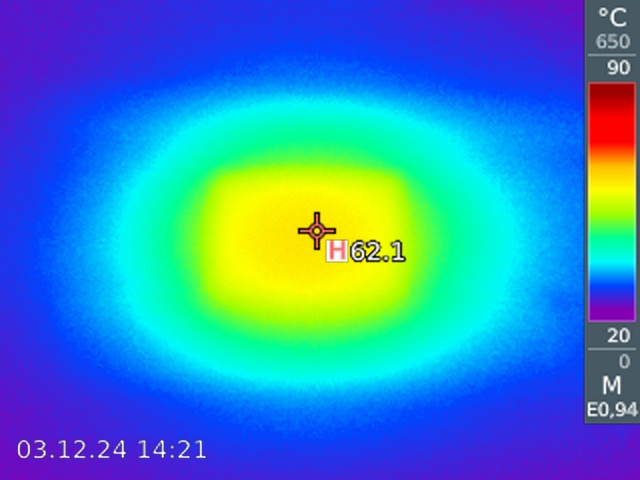

Supplement: S2 Figs — (ZIP) [file pone.0338325.s002.zip › image series/0. Cneg/TR004716.JPG]

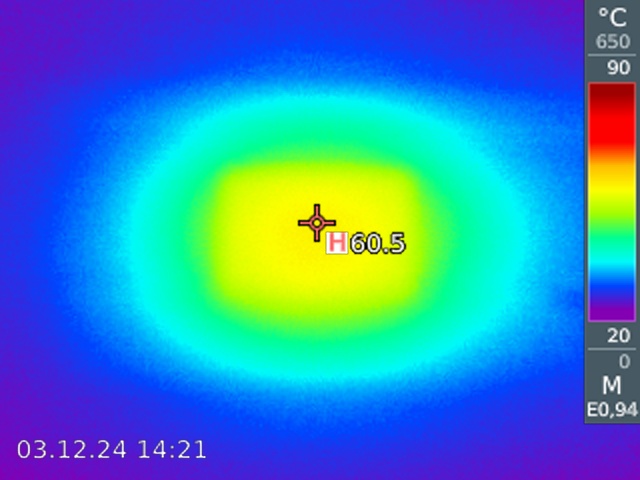

Supplement: S2 Figs — (ZIP) [file pone.0338325.s002.zip › image series/0. Cneg/TR004717.JPG]

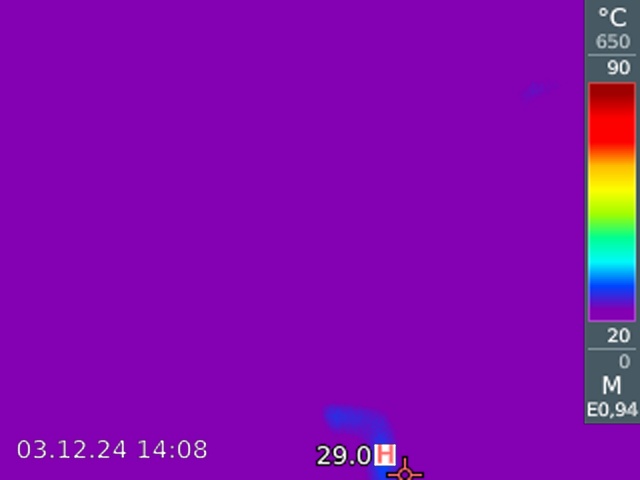

Supplement: S2 Figs — (ZIP) [file pone.0338325.s002.zip › image series/1. Cneg gel/TR004657.JPG]

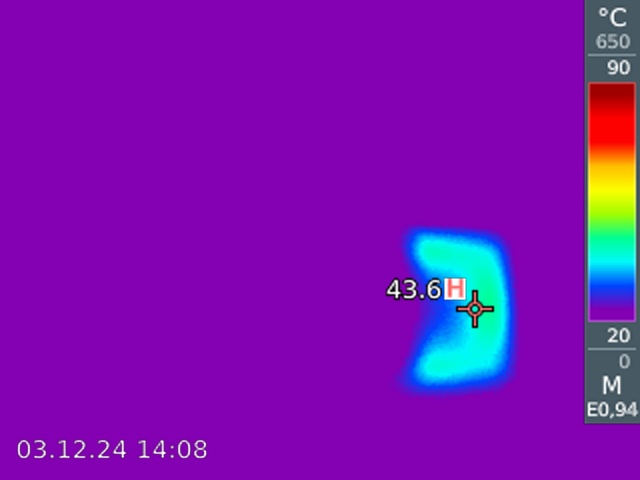

Supplement: S2 Figs — (ZIP) [file pone.0338325.s002.zip › image series/1. Cneg gel/TR004658.JPG]

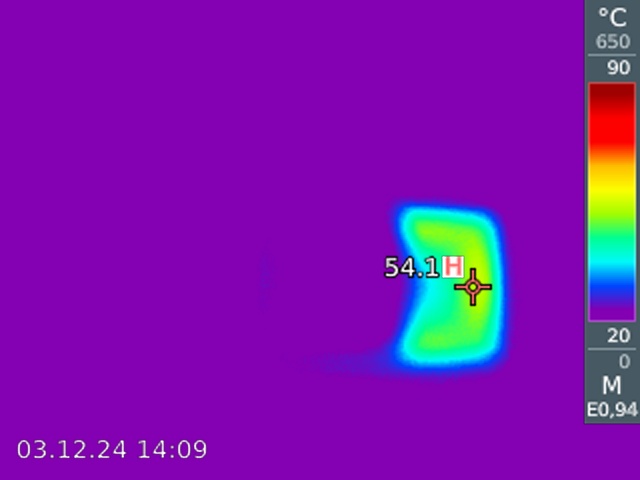

Supplement: S2 Figs — (ZIP) [file pone.0338325.s002.zip › image series/1. Cneg gel/TR004659.JPG]

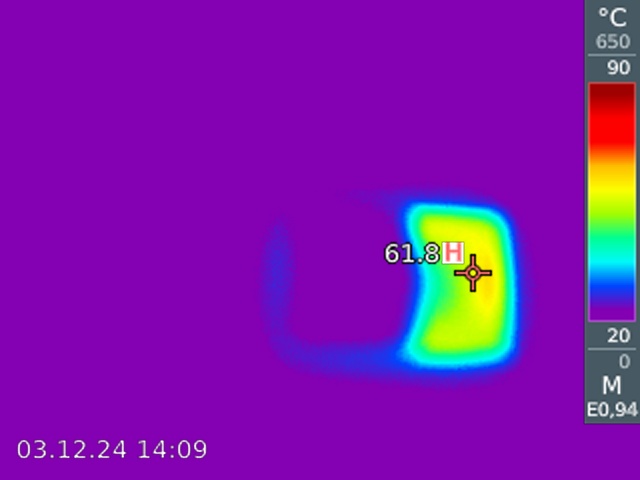

Supplement: S2 Figs — (ZIP) [file pone.0338325.s002.zip › image series/1. Cneg gel/TR004660.JPG]

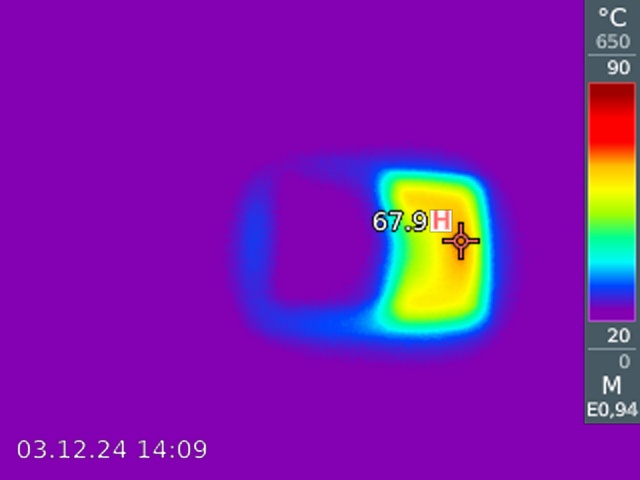

Supplement: S2 Figs — (ZIP) [file pone.0338325.s002.zip › image series/1. Cneg gel/TR004661.JPG]

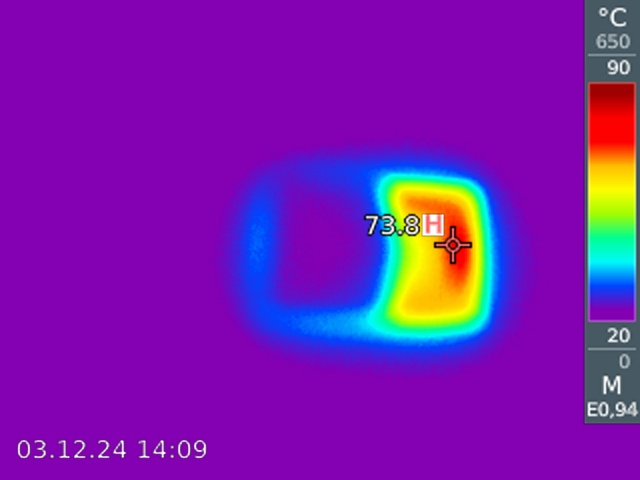

Supplement: S2 Figs — (ZIP) [file pone.0338325.s002.zip › image series/1. Cneg gel/TR004662.JPG]

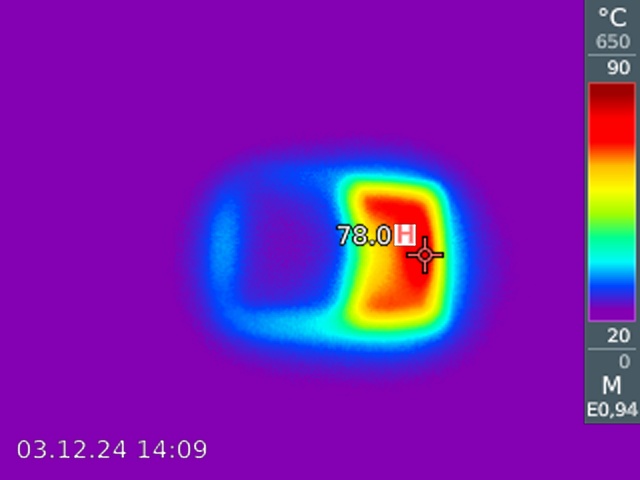

Supplement: S2 Figs — (ZIP) [file pone.0338325.s002.zip › image series/1. Cneg gel/TR004663.JPG]

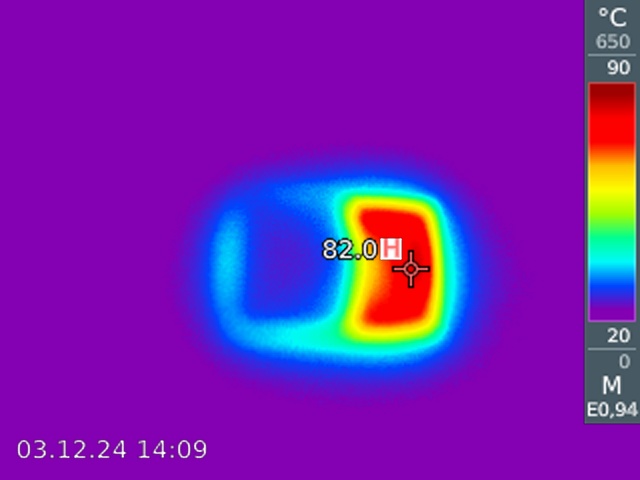

Supplement: S2 Figs — (ZIP) [file pone.0338325.s002.zip › image series/1. Cneg gel/TR004664.JPG]

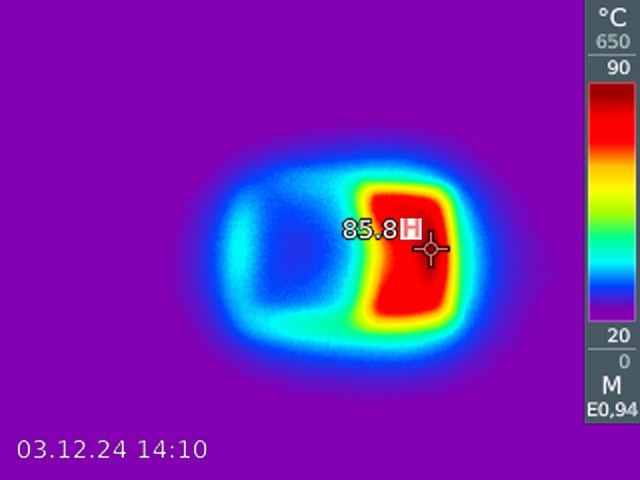

Supplement: S2 Figs — (ZIP) [file pone.0338325.s002.zip › image series/1. Cneg gel/TR004665.JPG]

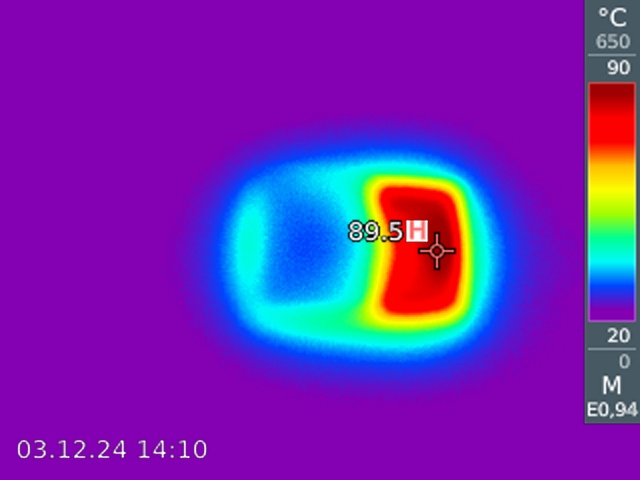

Supplement: S2 Figs — (ZIP) [file pone.0338325.s002.zip › image series/1. Cneg gel/TR004666.JPG]

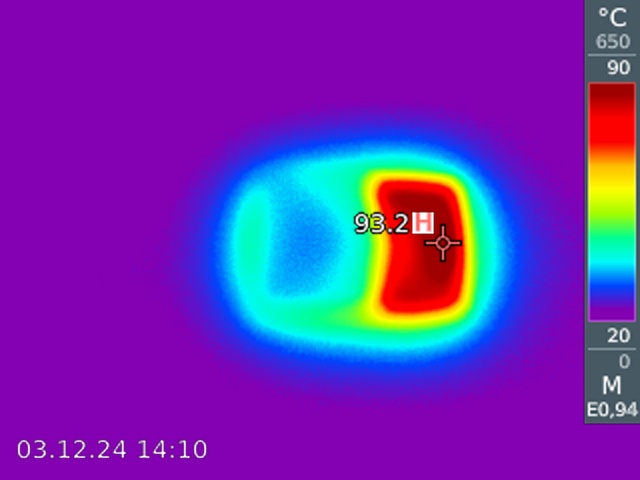

Supplement: S2 Figs — (ZIP) [file pone.0338325.s002.zip › image series/1. Cneg gel/TR004667.JPG]

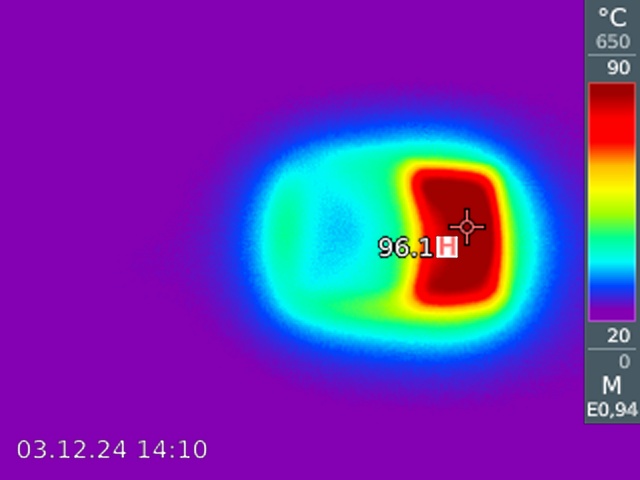

Supplement: S2 Figs — (ZIP) [file pone.0338325.s002.zip › image series/1. Cneg gel/TR004668.JPG]

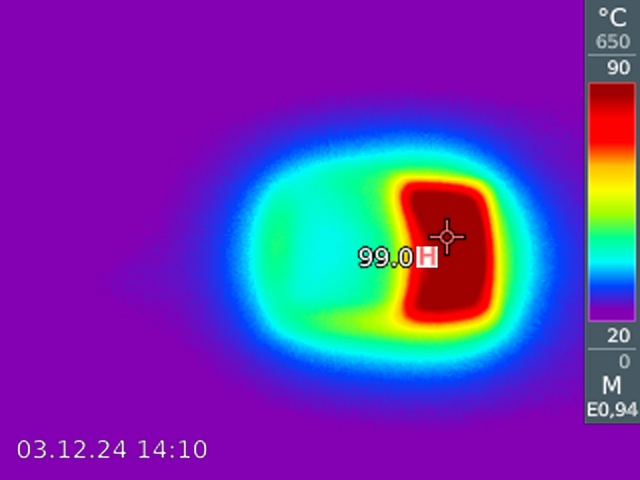

Supplement: S2 Figs — (ZIP) [file pone.0338325.s002.zip › image series/1. Cneg gel/TR004669.JPG]

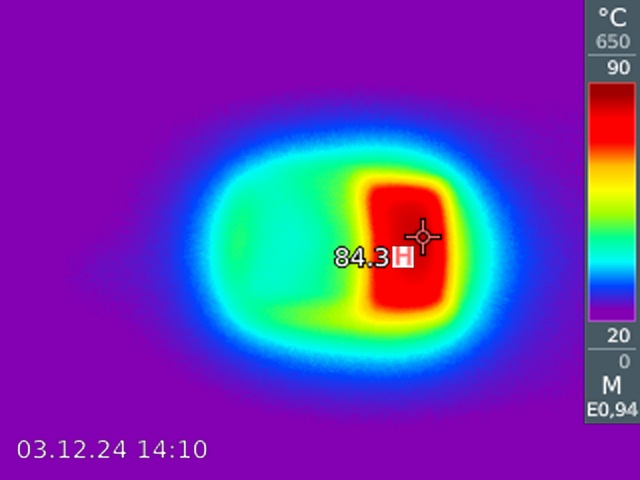

Supplement: S2 Figs — (ZIP) [file pone.0338325.s002.zip › image series/1. Cneg gel/TR004670.JPG]

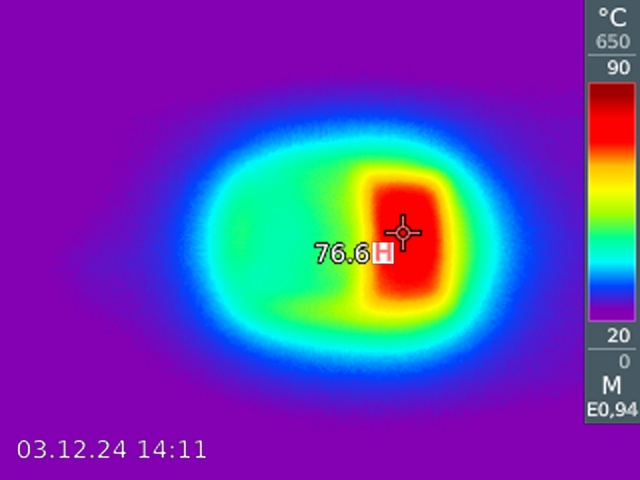

Supplement: S2 Figs — (ZIP) [file pone.0338325.s002.zip › image series/1. Cneg gel/TR004671.JPG]

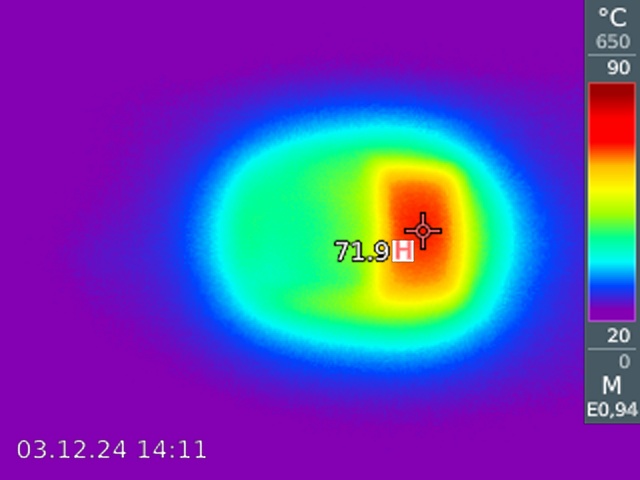

Supplement: S2 Figs — (ZIP) [file pone.0338325.s002.zip › image series/1. Cneg gel/TR004672.JPG]

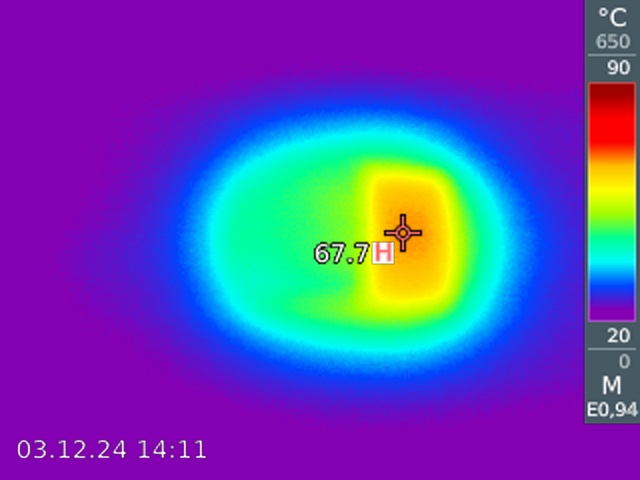

Supplement: S2 Figs — (ZIP) [file pone.0338325.s002.zip › image series/1. Cneg gel/TR004673.JPG]

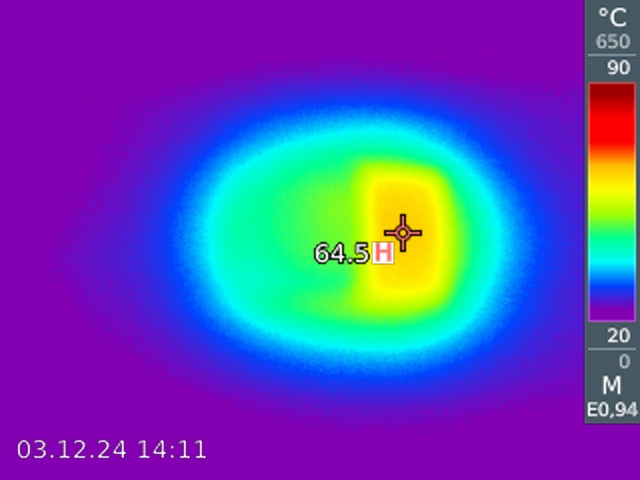

Supplement: S2 Figs — (ZIP) [file pone.0338325.s002.zip › image series/1. Cneg gel/TR004674.JPG]

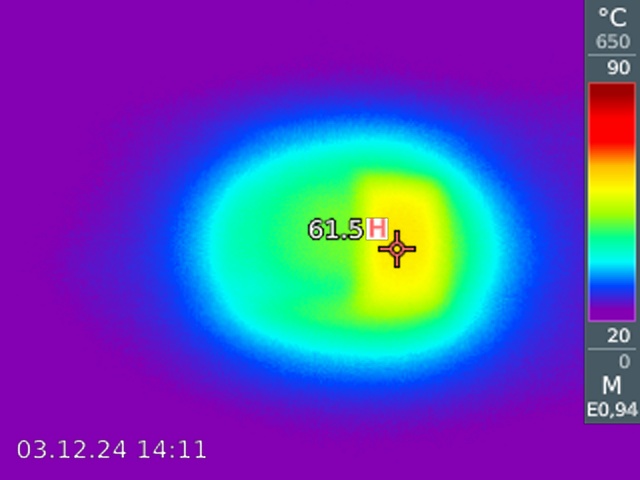

Supplement: S2 Figs — (ZIP) [file pone.0338325.s002.zip › image series/1. Cneg gel/TR004675.JPG]

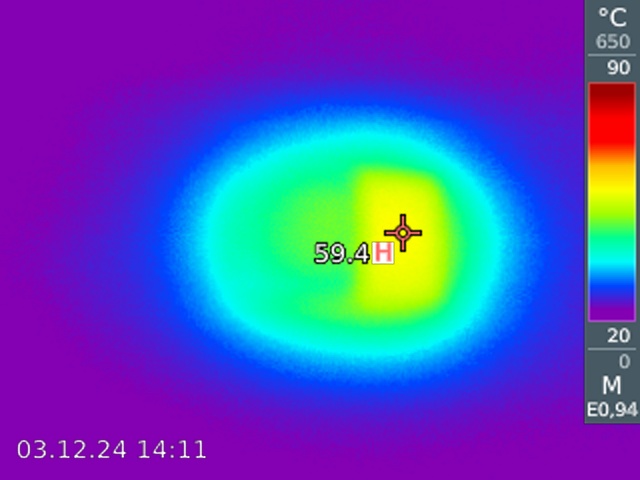

Supplement: S2 Figs — (ZIP) [file pone.0338325.s002.zip › image series/1. Cneg gel/TR004676.JPG]

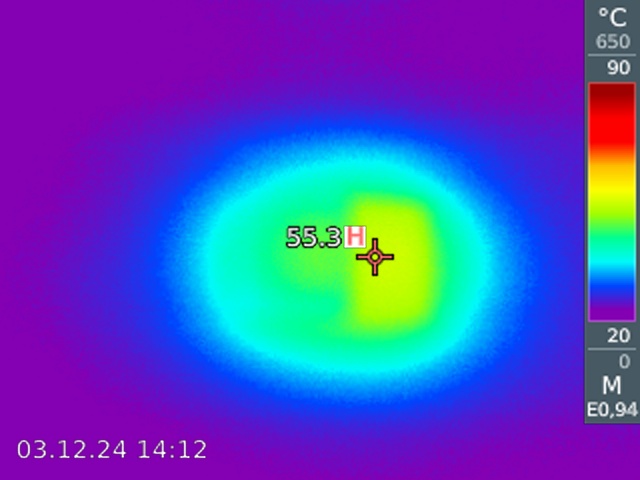

Supplement: S2 Figs — (ZIP) [file pone.0338325.s002.zip › image series/1. Cneg gel/TR004677.JPG]

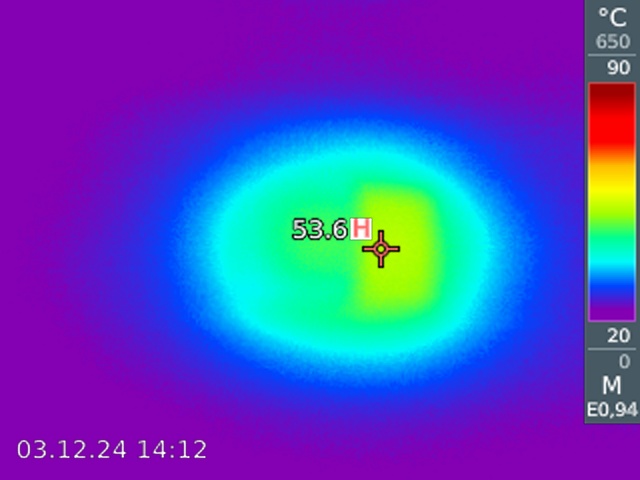

Supplement: S2 Figs — (ZIP) [file pone.0338325.s002.zip › image series/1. Cneg gel/TR004678.JPG]

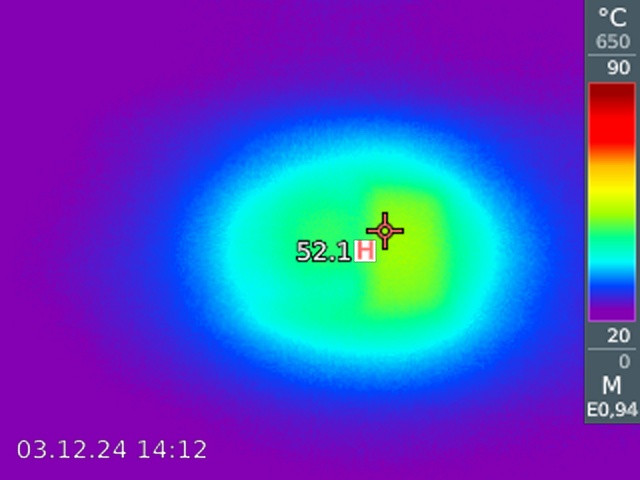

Supplement: S2 Figs — (ZIP) [file pone.0338325.s002.zip › image series/1. Cneg gel/TR004679.JPG]

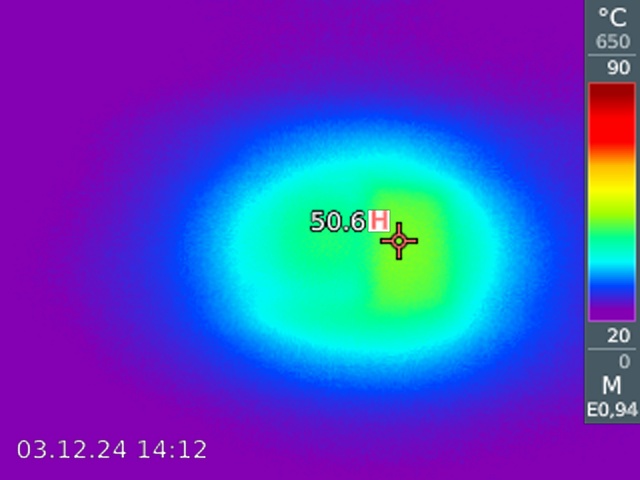

Supplement: S2 Figs — (ZIP) [file pone.0338325.s002.zip › image series/1. Cneg gel/TR004680.JPG]

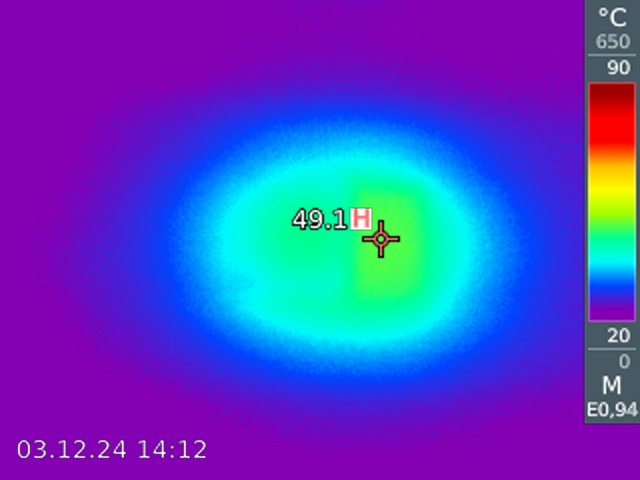

Supplement: S2 Figs — (ZIP) [file pone.0338325.s002.zip › image series/1. Cneg gel/TR004681.JPG]

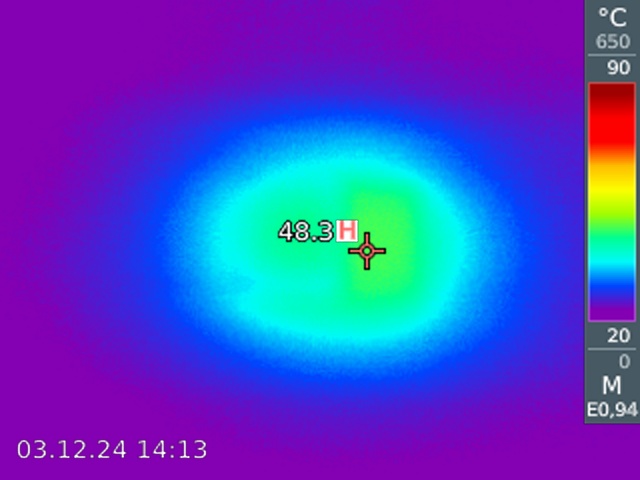

Supplement: S2 Figs — (ZIP) [file pone.0338325.s002.zip › image series/1. Cneg gel/TR004682.JPG]

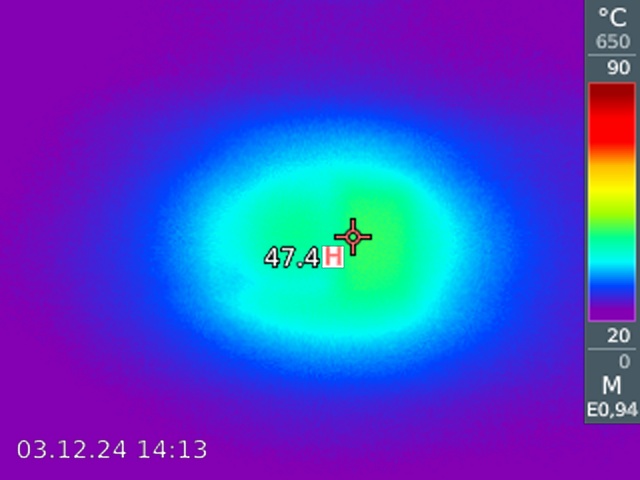

Supplement: S2 Figs — (ZIP) [file pone.0338325.s002.zip › image series/1. Cneg gel/TR004683.JPG]

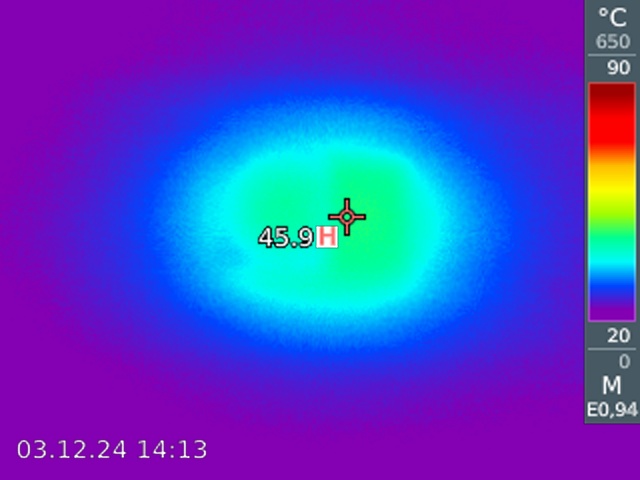

Supplement: S2 Figs — (ZIP) [file pone.0338325.s002.zip › image series/1. Cneg gel/TR004684.JPG]

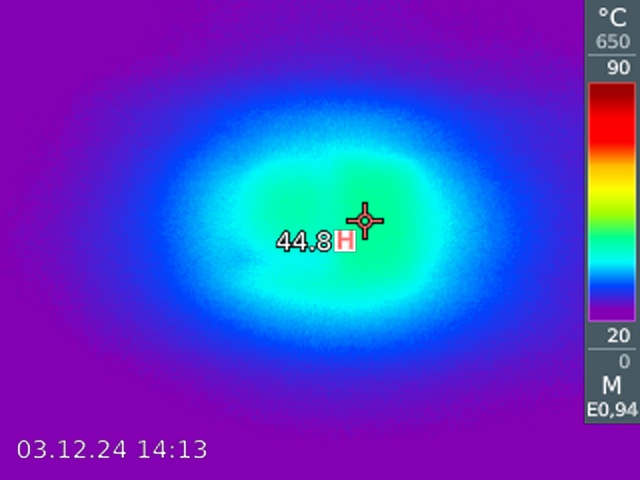

Supplement: S2 Figs — (ZIP) [file pone.0338325.s002.zip › image series/1. Cneg gel/TR004685.JPG]

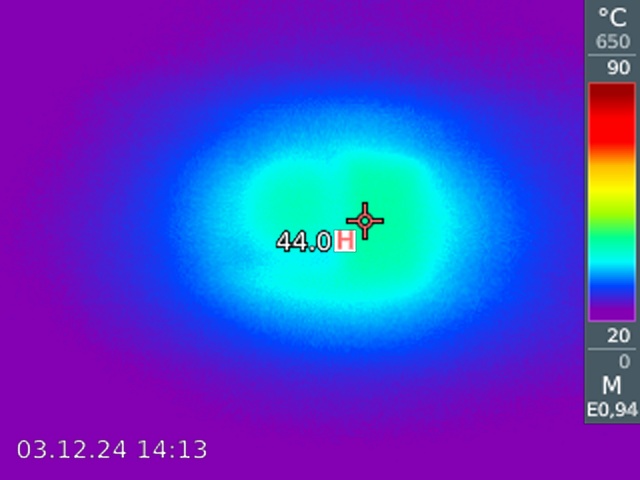

Supplement: S2 Figs — (ZIP) [file pone.0338325.s002.zip › image series/1. Cneg gel/TR004686.JPG]

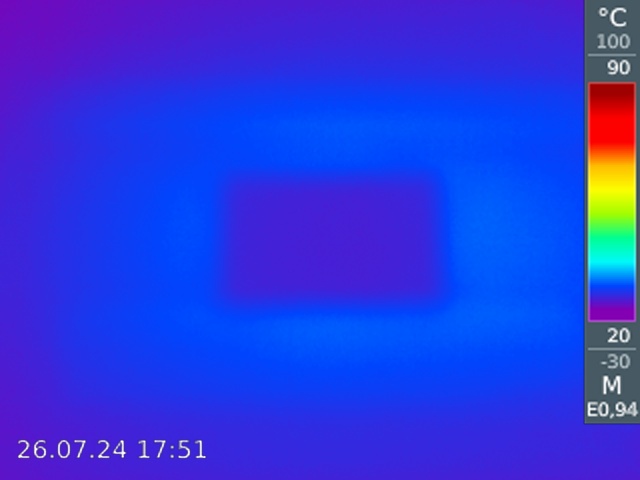

Supplement: S2 Figs — (ZIP) [file pone.0338325.s002.zip › image series/2. HA/TR004606.JPG]

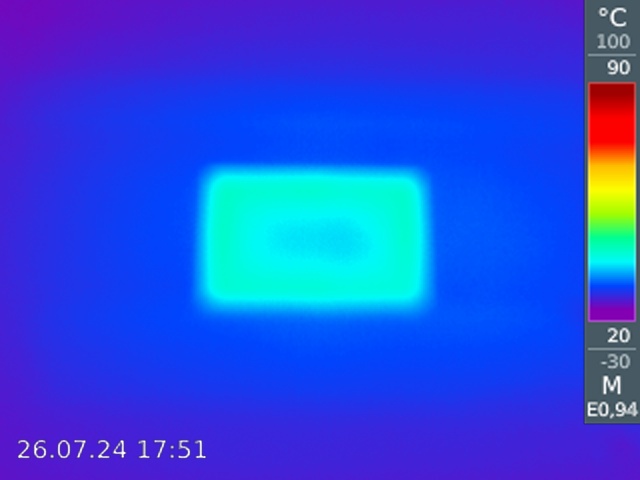

Supplement: S2 Figs — (ZIP) [file pone.0338325.s002.zip › image series/2. HA/TR004607.JPG]

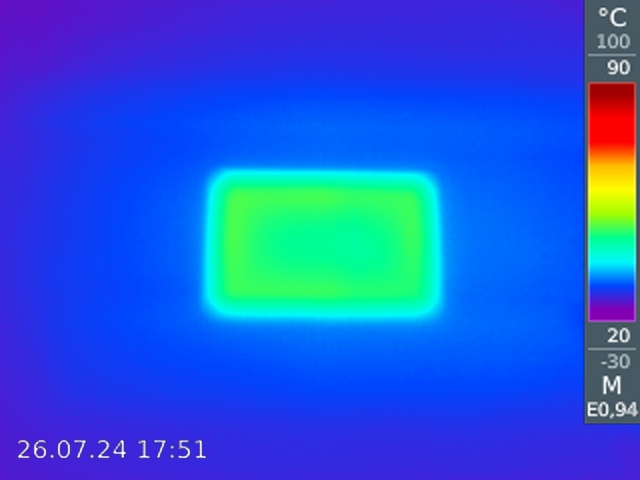

Supplement: S2 Figs — (ZIP) [file pone.0338325.s002.zip › image series/2. HA/TR004608.JPG]

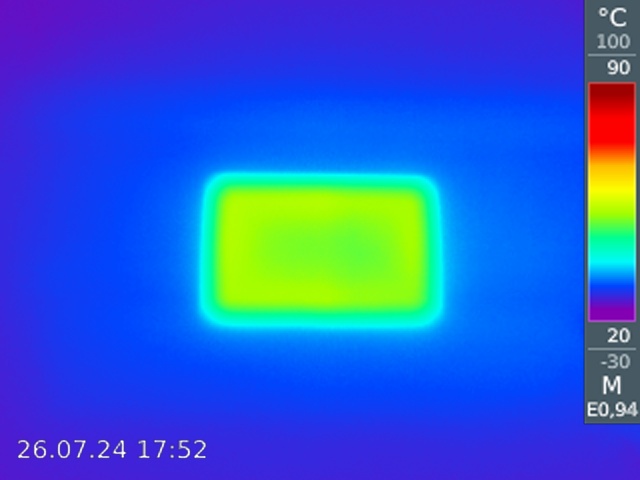

Supplement: S2 Figs — (ZIP) [file pone.0338325.s002.zip › image series/2. HA/TR004609.JPG]

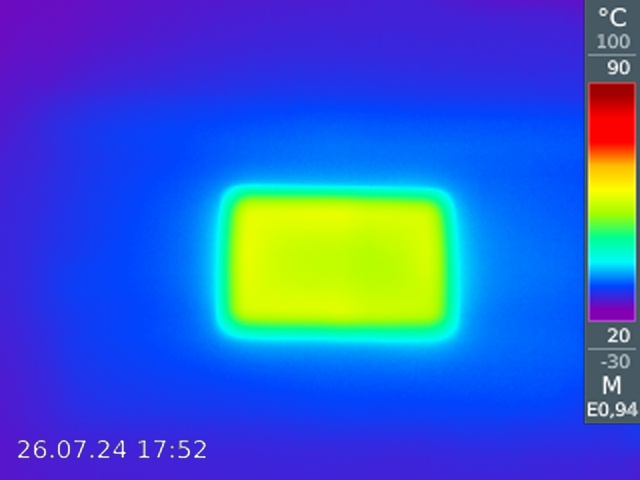

Supplement: S2 Figs — (ZIP) [file pone.0338325.s002.zip › image series/2. HA/TR004610.JPG]

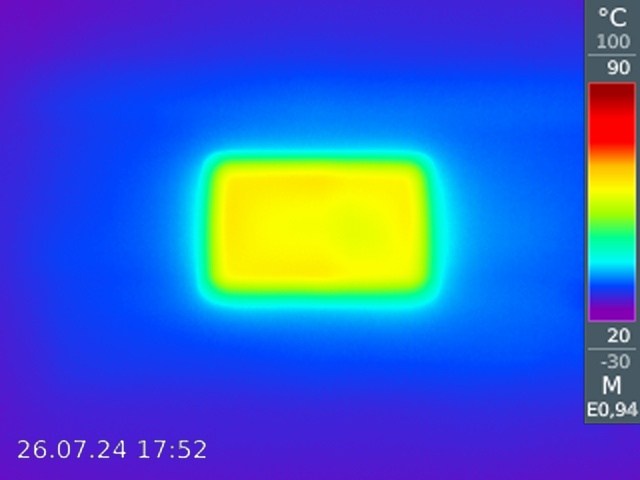

Supplement: S2 Figs — (ZIP) [file pone.0338325.s002.zip › image series/2. HA/TR004611.JPG]

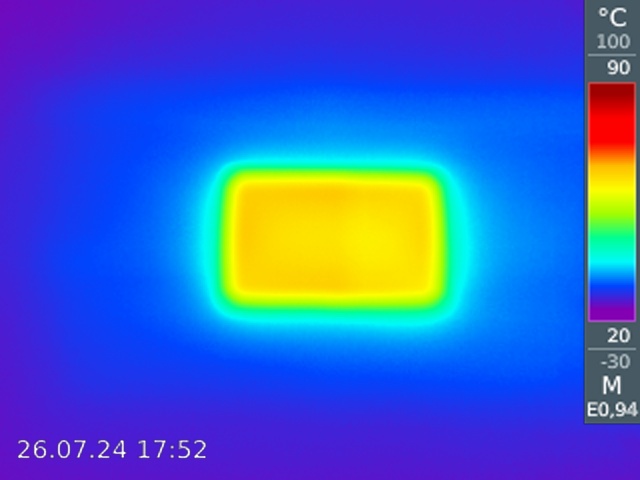

Supplement: S2 Figs — (ZIP) [file pone.0338325.s002.zip › image series/2. HA/TR004612.JPG]

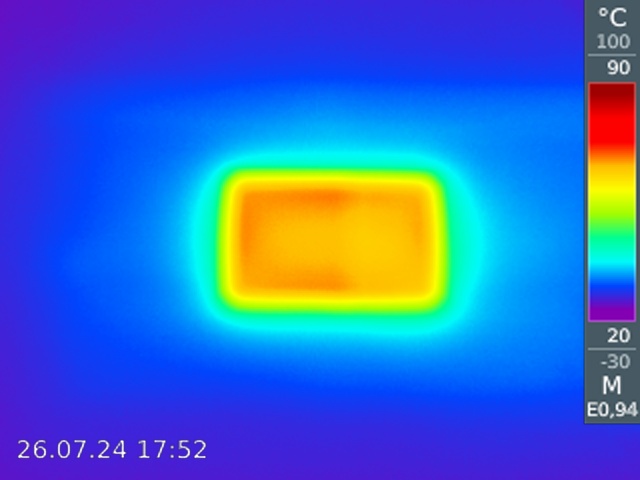

Supplement: S2 Figs — (ZIP) [file pone.0338325.s002.zip › image series/2. HA/TR004613.JPG]

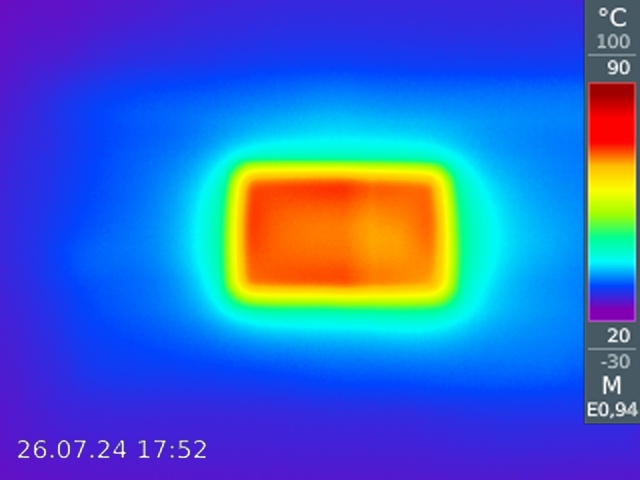

Supplement: S2 Figs — (ZIP) [file pone.0338325.s002.zip › image series/2. HA/TR004614.JPG]

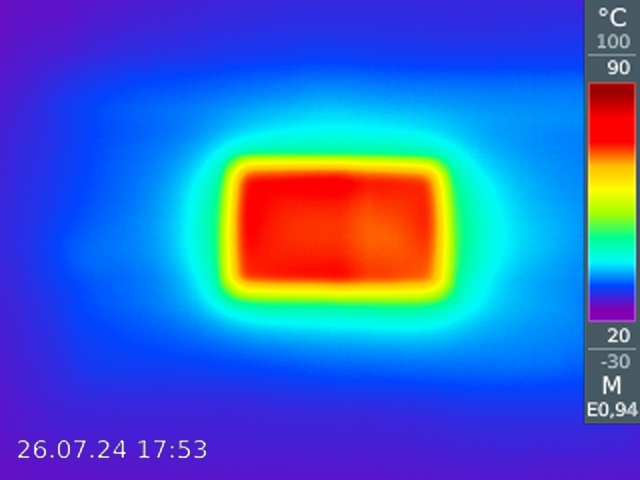

Supplement: S2 Figs — (ZIP) [file pone.0338325.s002.zip › image series/2. HA/TR004615.JPG]

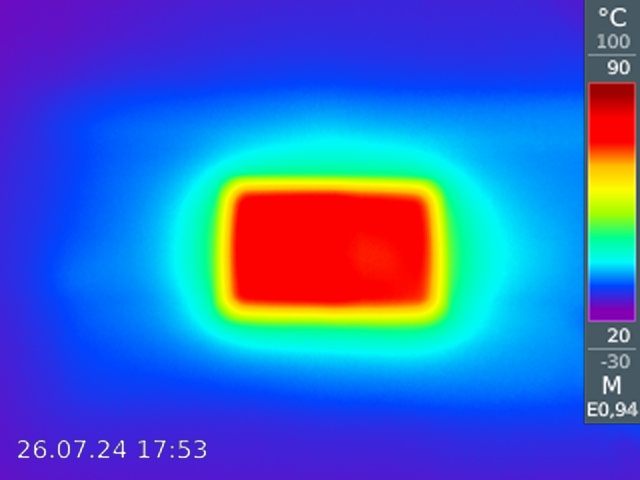

Supplement: S2 Figs — (ZIP) [file pone.0338325.s002.zip › image series/2. HA/TR004616.JPG]

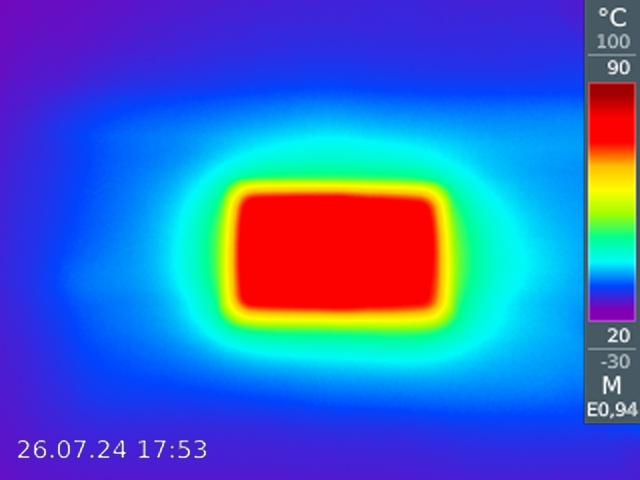

Supplement: S2 Figs — (ZIP) [file pone.0338325.s002.zip › image series/2. HA/TR004617.JPG]

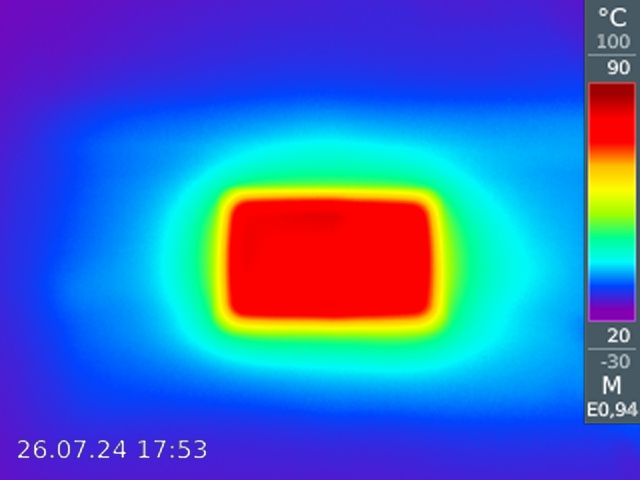

Supplement: S2 Figs — (ZIP) [file pone.0338325.s002.zip › image series/2. HA/TR004618.JPG]

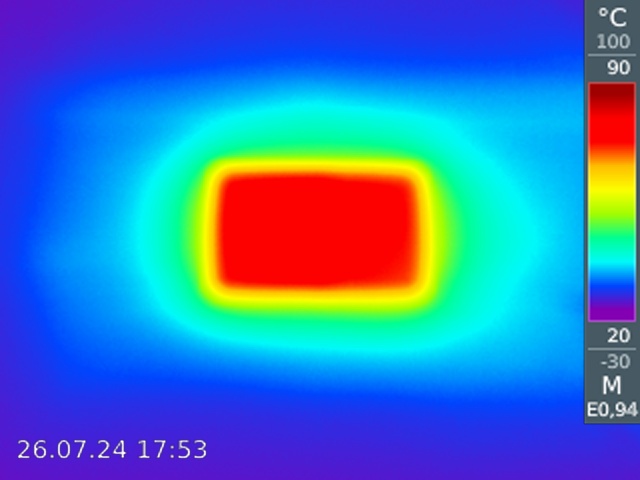

Supplement: S2 Figs — (ZIP) [file pone.0338325.s002.zip › image series/2. HA/TR004619.JPG]

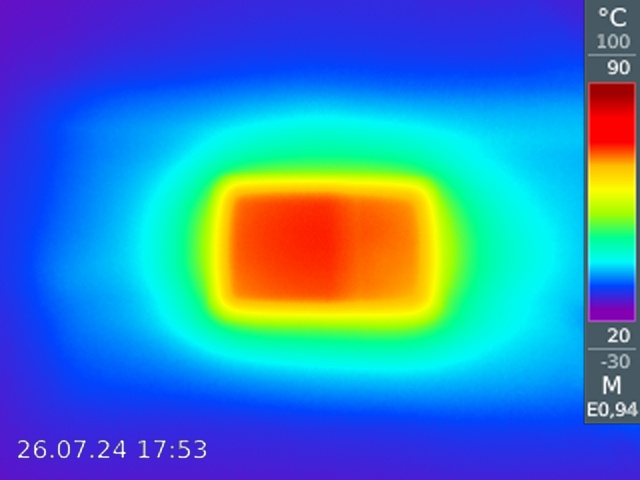

Supplement: S2 Figs — (ZIP) [file pone.0338325.s002.zip › image series/2. HA/TR004620.JPG]

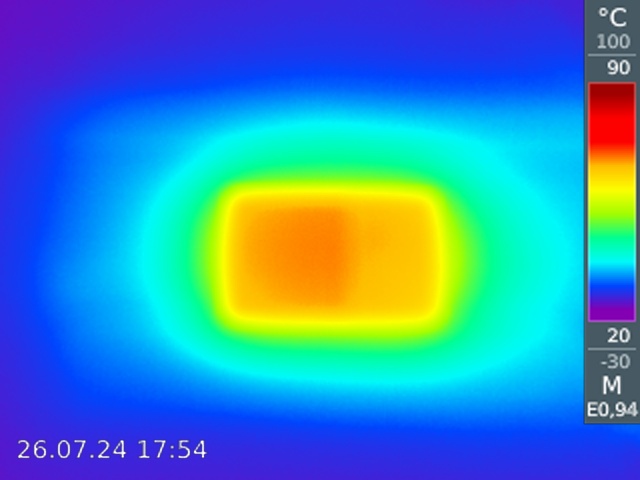

Supplement: S2 Figs — (ZIP) [file pone.0338325.s002.zip › image series/2. HA/TR004621.JPG]

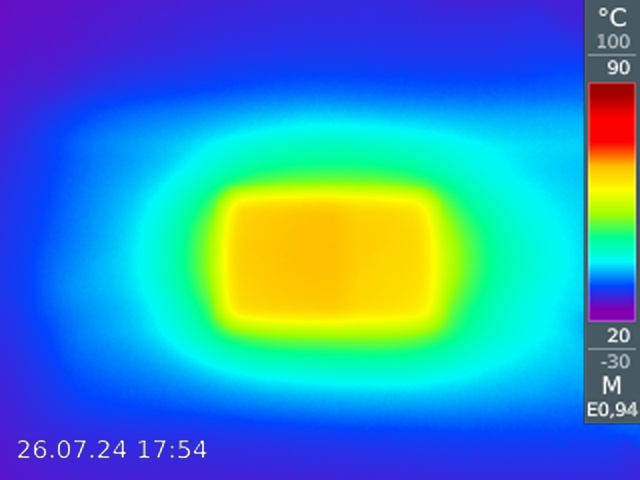

Supplement: S2 Figs — (ZIP) [file pone.0338325.s002.zip › image series/2. HA/TR004622.JPG]

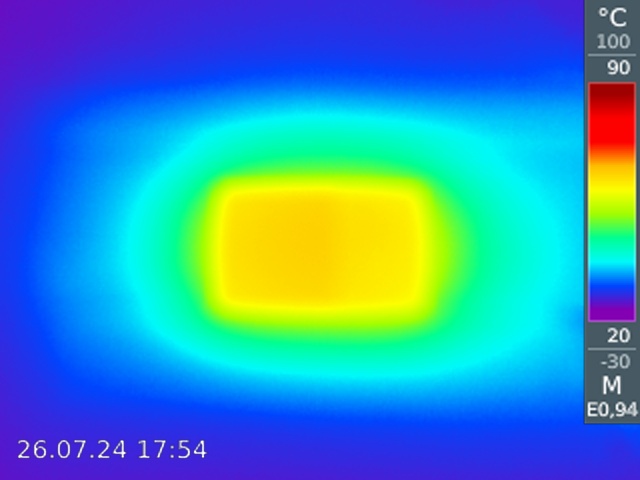

Supplement: S2 Figs — (ZIP) [file pone.0338325.s002.zip › image series/2. HA/TR004623.JPG]

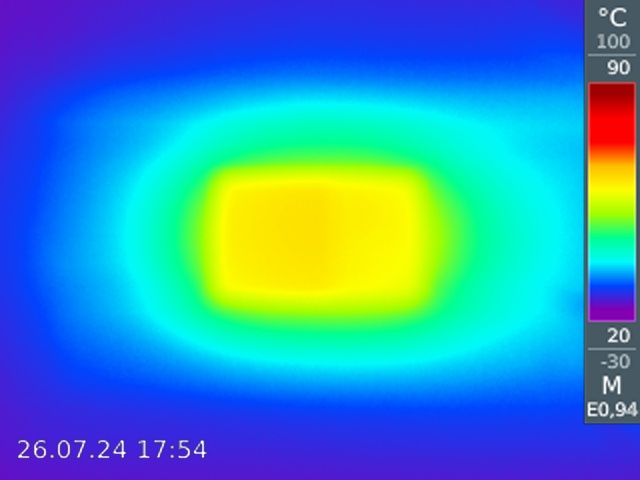

Supplement: S2 Figs — (ZIP) [file pone.0338325.s002.zip › image series/2. HA/TR004624.JPG]

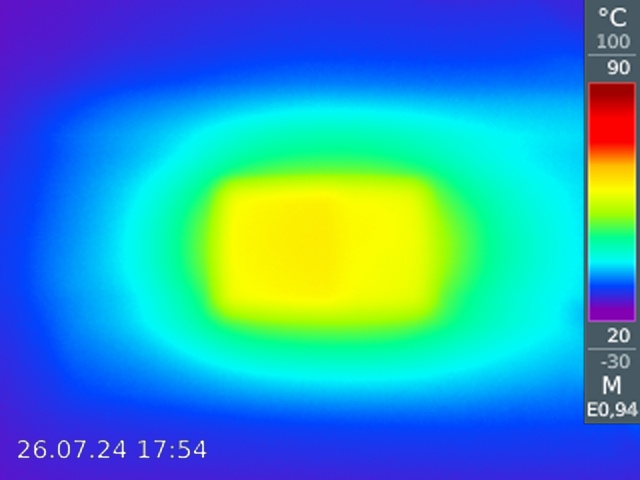

Supplement: S2 Figs — (ZIP) [file pone.0338325.s002.zip › image series/2. HA/TR004625.JPG]

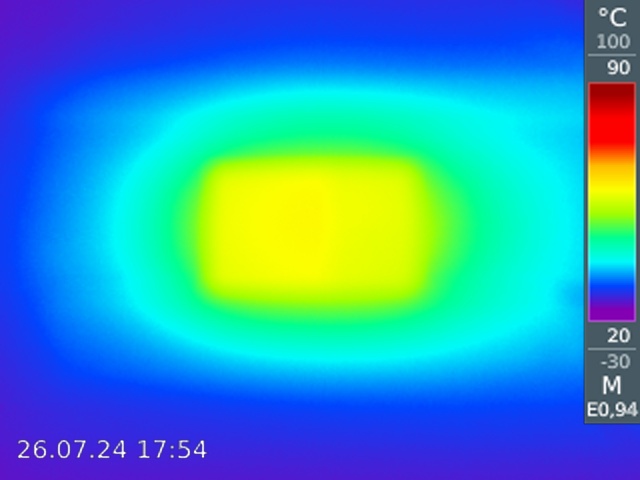

Supplement: S2 Figs — (ZIP) [file pone.0338325.s002.zip › image series/2. HA/TR004626.JPG]

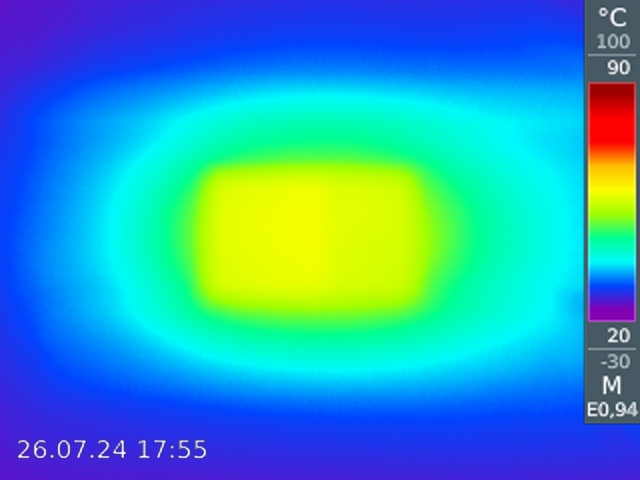

Supplement: S2 Figs — (ZIP) [file pone.0338325.s002.zip › image series/2. HA/TR004627.JPG]

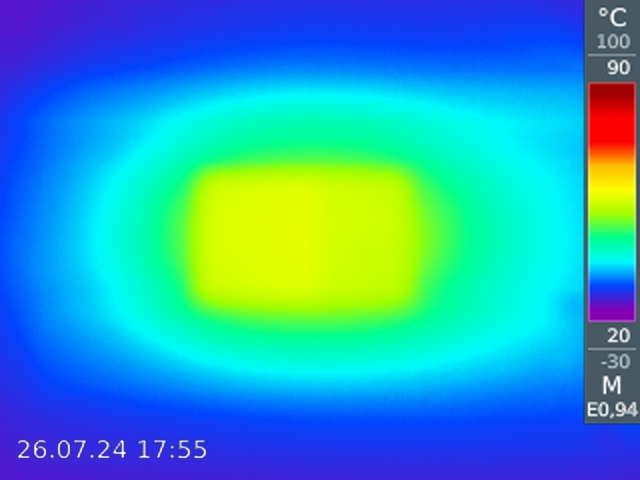

Supplement: S2 Figs — (ZIP) [file pone.0338325.s002.zip › image series/2. HA/TR004628.JPG]

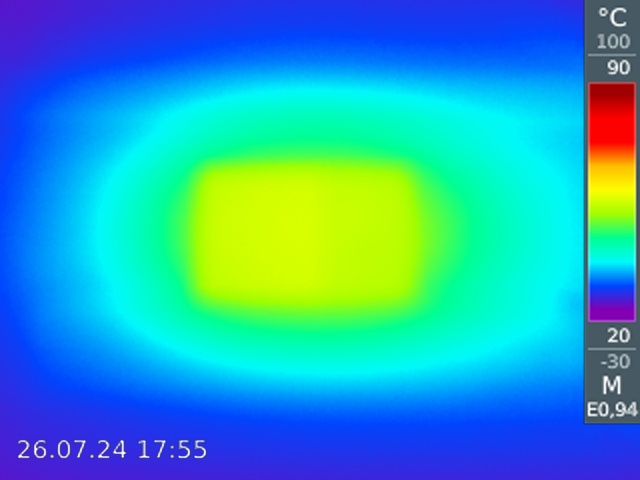

Supplement: S2 Figs — (ZIP) [file pone.0338325.s002.zip › image series/2. HA/TR004629.JPG]

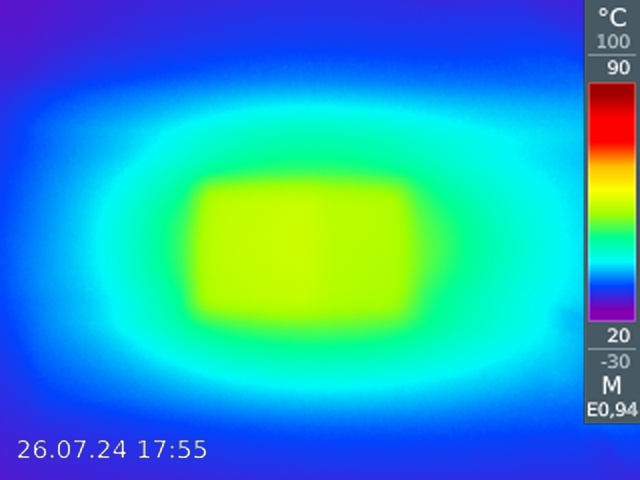

Supplement: S2 Figs — (ZIP) [file pone.0338325.s002.zip › image series/2. HA/TR004630.JPG]

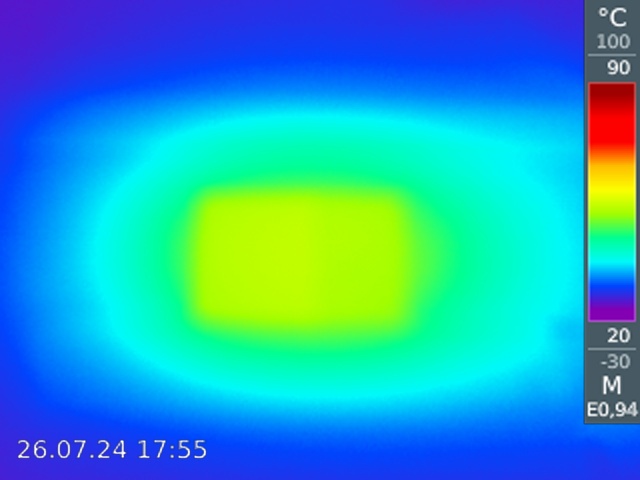

Supplement: S2 Figs — (ZIP) [file pone.0338325.s002.zip › image series/2. HA/TR004631.JPG]

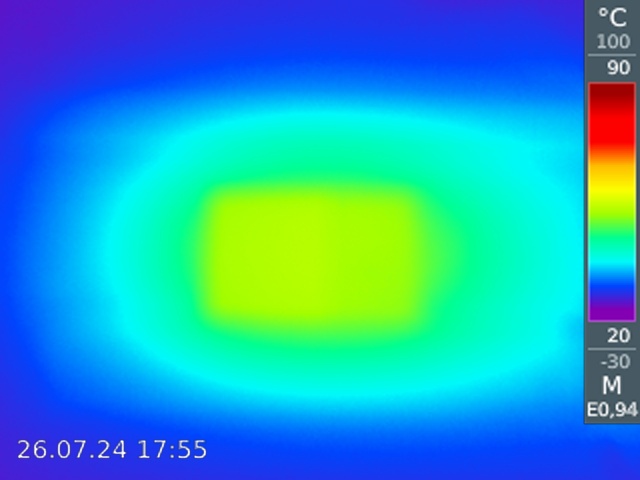

Supplement: S2 Figs — (ZIP) [file pone.0338325.s002.zip › image series/2. HA/TR004632.JPG]

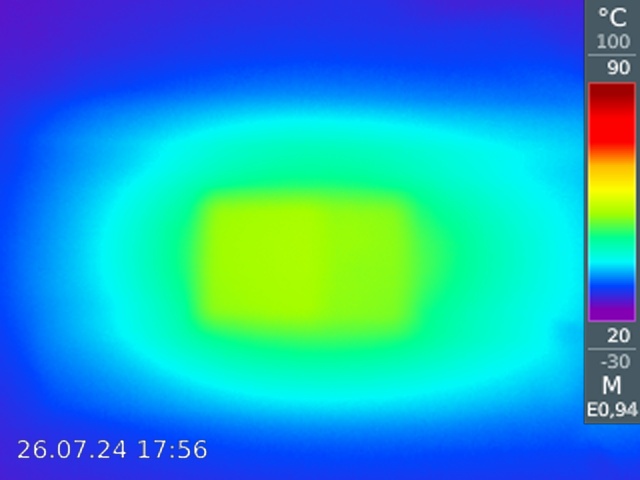

Supplement: S2 Figs — (ZIP) [file pone.0338325.s002.zip › image series/2. HA/TR004633.JPG]

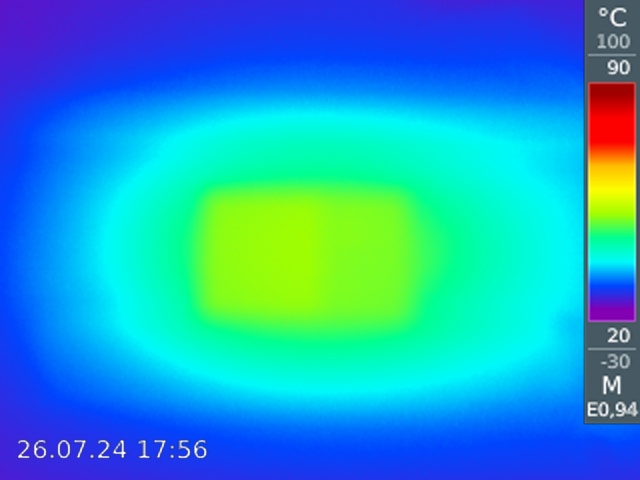

Supplement: S2 Figs — (ZIP) [file pone.0338325.s002.zip › image series/2. HA/TR004634.JPG]

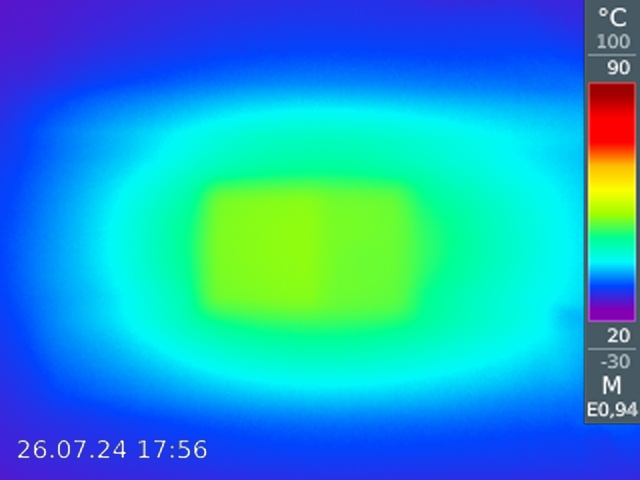

Supplement: S2 Figs — (ZIP) [file pone.0338325.s002.zip › image series/2. HA/TR004635.JPG]

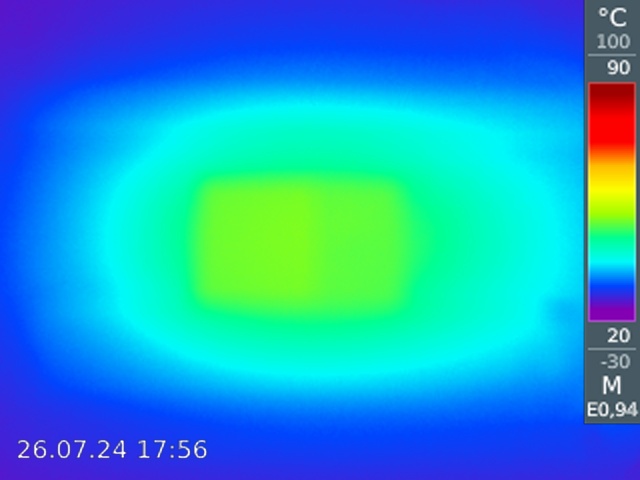

Supplement: S2 Figs — (ZIP) [file pone.0338325.s002.zip › image series/2. HA/TR004636.JPG]

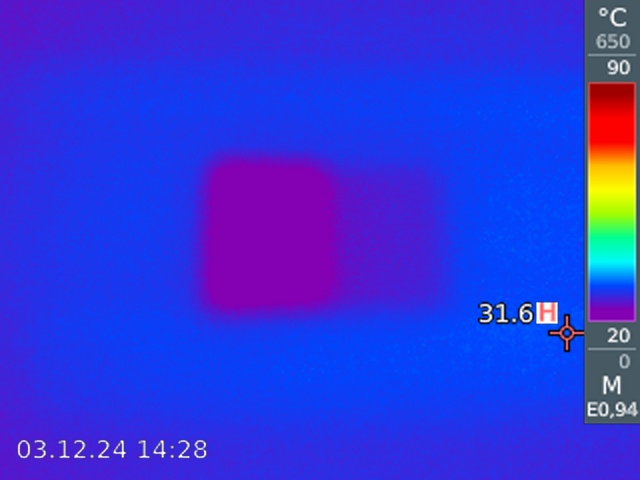

Supplement: S2 Figs — (ZIP) [file pone.0338325.s002.zip › image series/3. HA gel/TR004718.JPG]

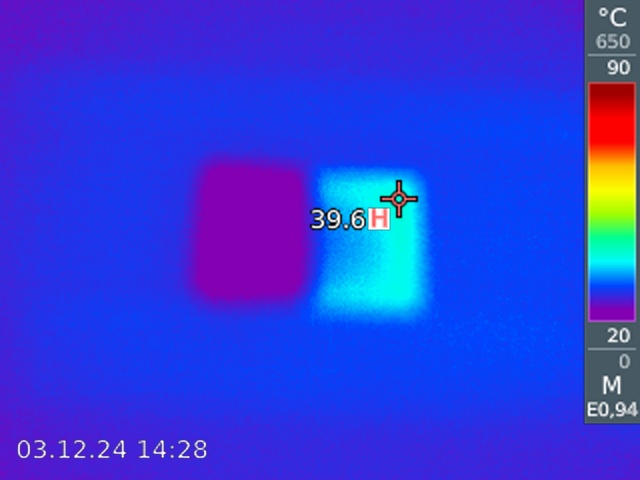

Supplement: S2 Figs — (ZIP) [file pone.0338325.s002.zip › image series/3. HA gel/TR004719.JPG]

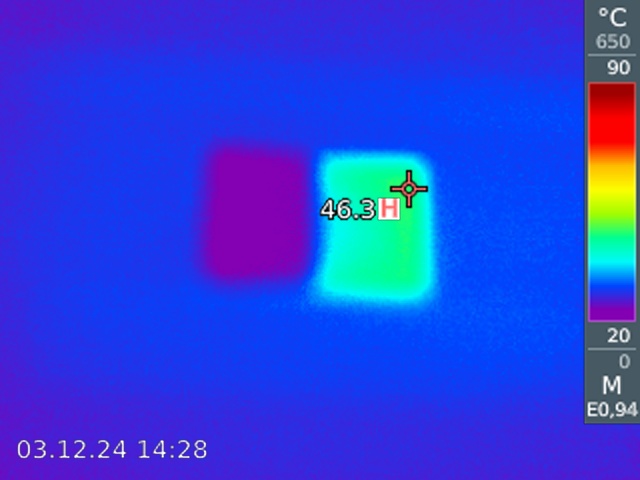

Supplement: S2 Figs — (ZIP) [file pone.0338325.s002.zip › image series/3. HA gel/TR004720.JPG]

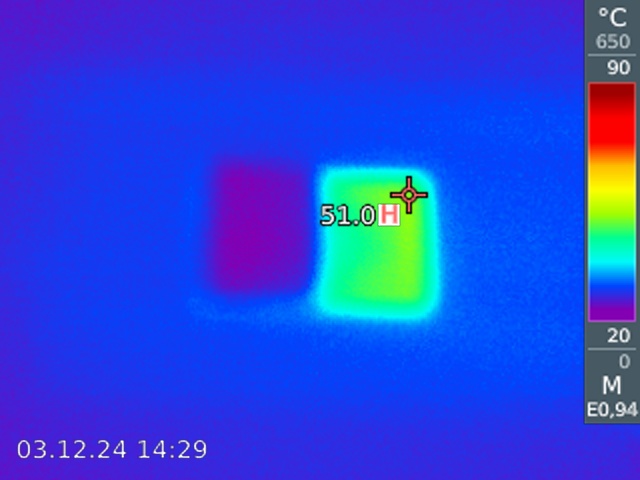

Supplement: S2 Figs — (ZIP) [file pone.0338325.s002.zip › image series/3. HA gel/TR004721.JPG]

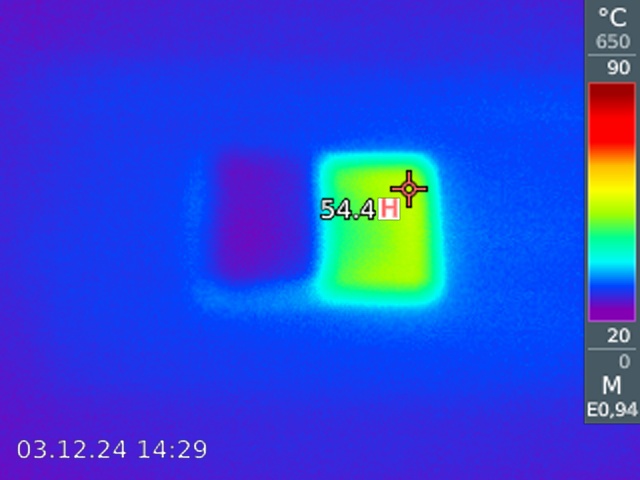

Supplement: S2 Figs — (ZIP) [file pone.0338325.s002.zip › image series/3. HA gel/TR004722.JPG]

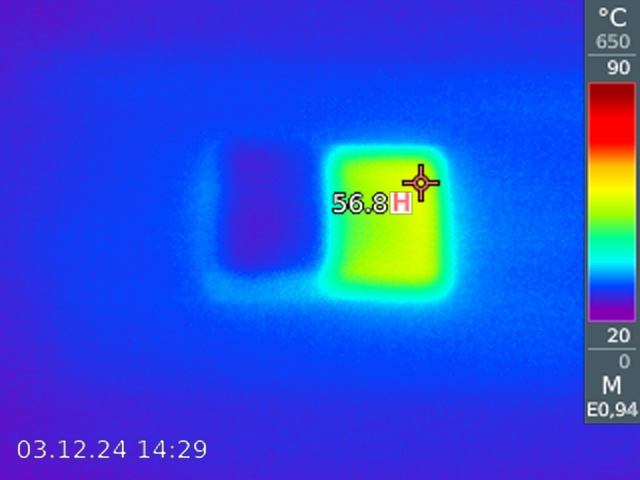

Supplement: S2 Figs — (ZIP) [file pone.0338325.s002.zip › image series/3. HA gel/TR004723.JPG]

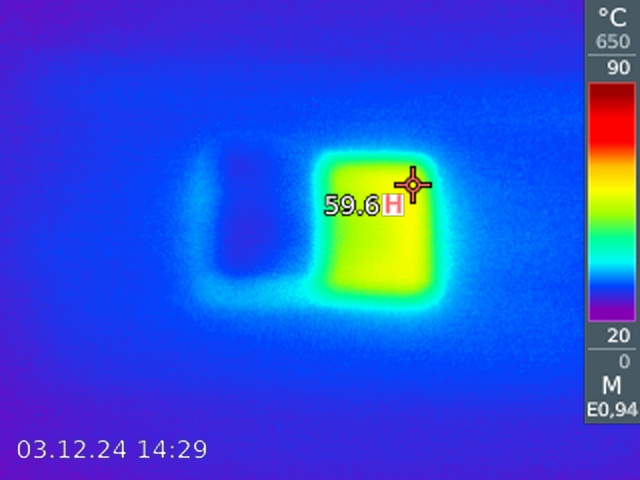

Supplement: S2 Figs — (ZIP) [file pone.0338325.s002.zip › image series/3. HA gel/TR004724.JPG]
