# Supplementary figures and images for: Thermal insulation of poly(methyl methacrylate) bone cement and hydroxyapatite coatings under induction heating of metal implants (part 3 of 3)
Source: PLoS One. 2025 Dec 11;20(12):e0338325. doi: 10.1371/journal.pone.0338325 (PMC12698007; doi:10.1371/journal.pone.0338325)

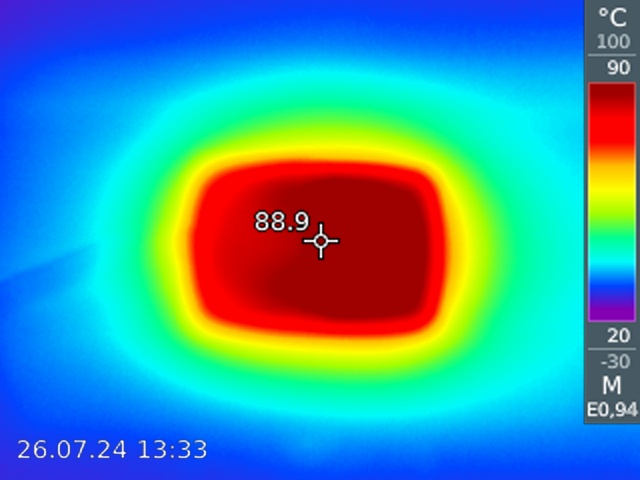

Supplement: S2 Figs — (ZIP) [file pone.0338325.s002.zip › image series/6. 3mm/TR004482.JPG]
