# Supplementary figures and images for: Thermal insulation of poly(methyl methacrylate) bone cement and hydroxyapatite coatings under induction heating of metal implants (part 2 of 3)
Source: PLoS One. 2025 Dec 11;20(12):e0338325. doi: 10.1371/journal.pone.0338325 (PMC12698007; doi:10.1371/journal.pone.0338325)

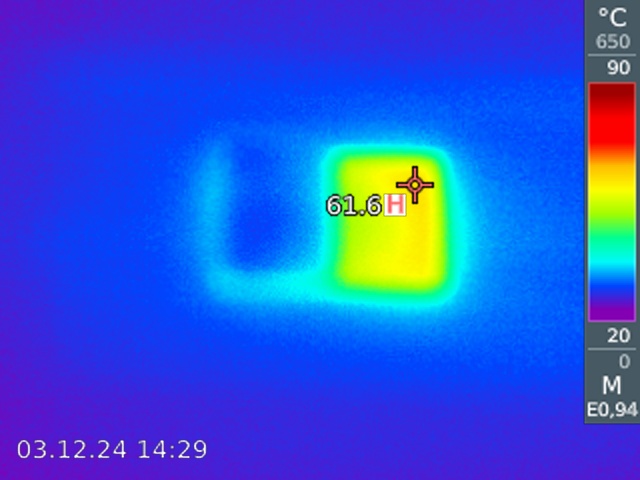

Supplement: S2 Figs — (ZIP) [file pone.0338325.s002.zip › image series/3. HA gel/TR004725.JPG]

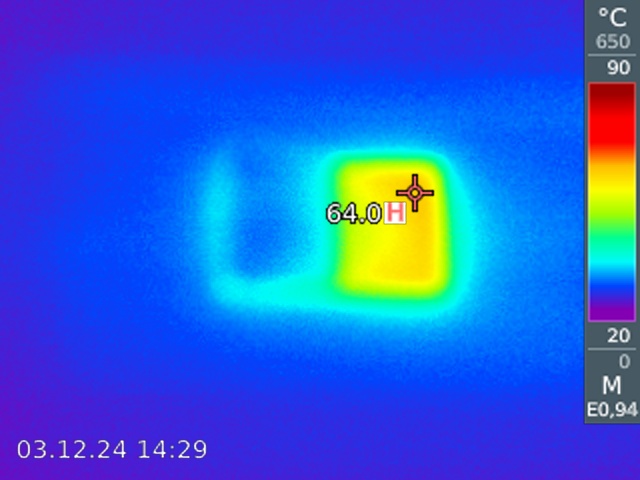

Supplement: S2 Figs — (ZIP) [file pone.0338325.s002.zip › image series/3. HA gel/TR004726.JPG]

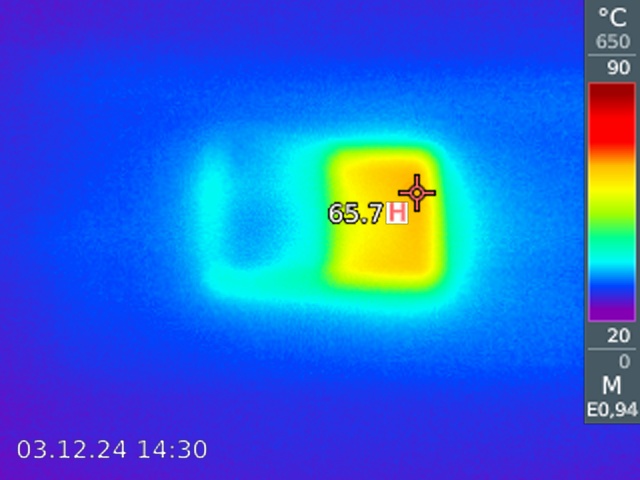

Supplement: S2 Figs — (ZIP) [file pone.0338325.s002.zip › image series/3. HA gel/TR004727.JPG]

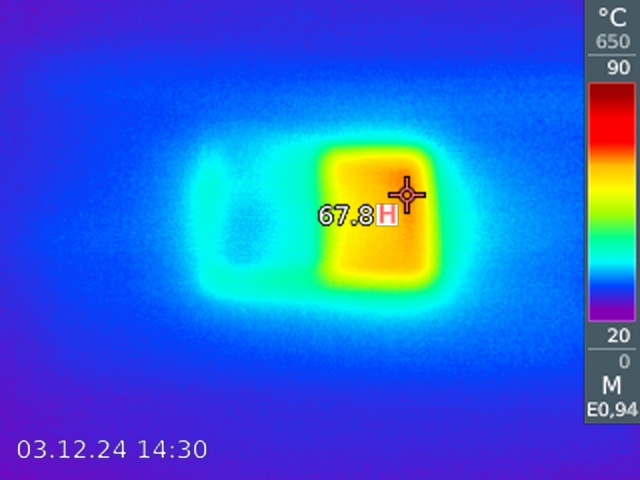

Supplement: S2 Figs — (ZIP) [file pone.0338325.s002.zip › image series/3. HA gel/TR004728.JPG]

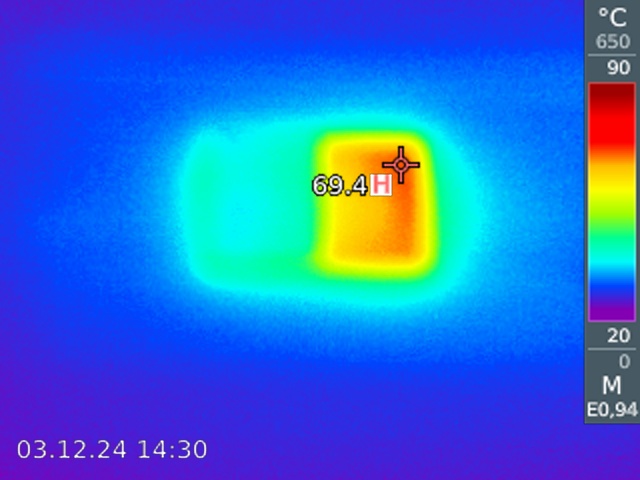

Supplement: S2 Figs — (ZIP) [file pone.0338325.s002.zip › image series/3. HA gel/TR004729.JPG]

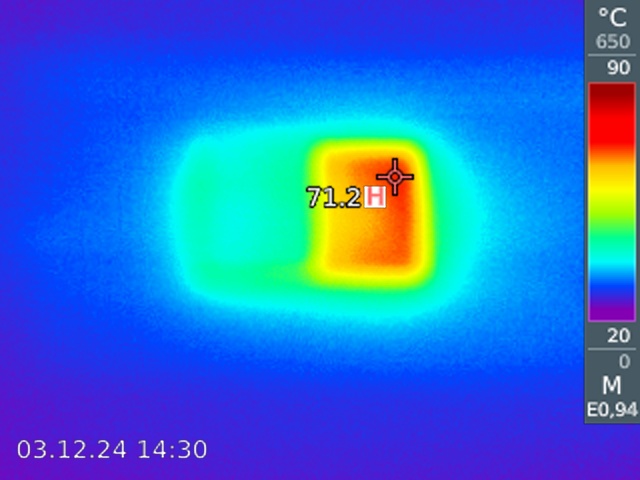

Supplement: S2 Figs — (ZIP) [file pone.0338325.s002.zip › image series/3. HA gel/TR004730.JPG]

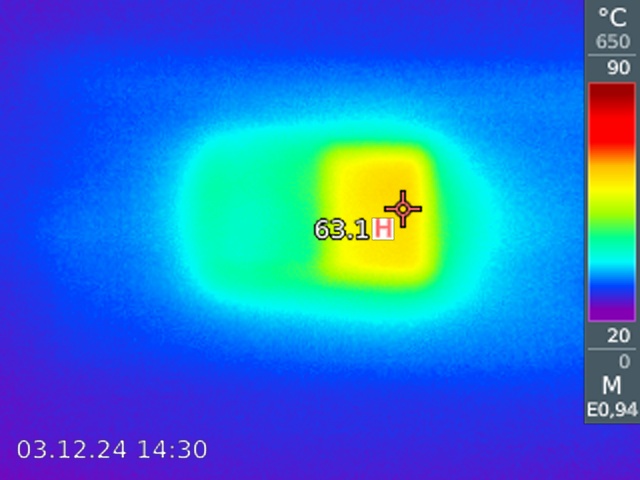

Supplement: S2 Figs — (ZIP) [file pone.0338325.s002.zip › image series/3. HA gel/TR004731.JPG]

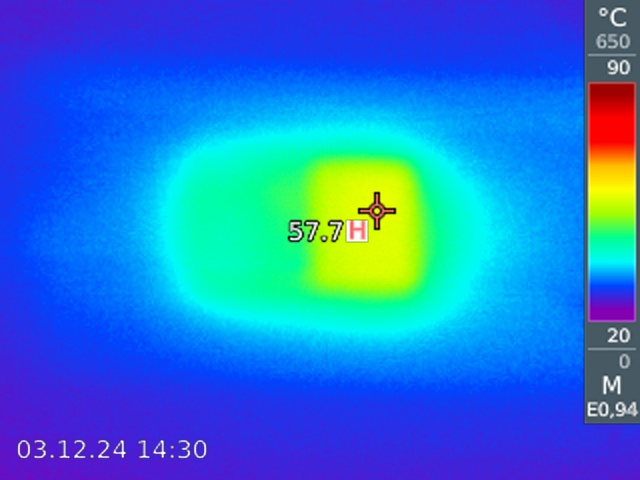

Supplement: S2 Figs — (ZIP) [file pone.0338325.s002.zip › image series/3. HA gel/TR004732.JPG]

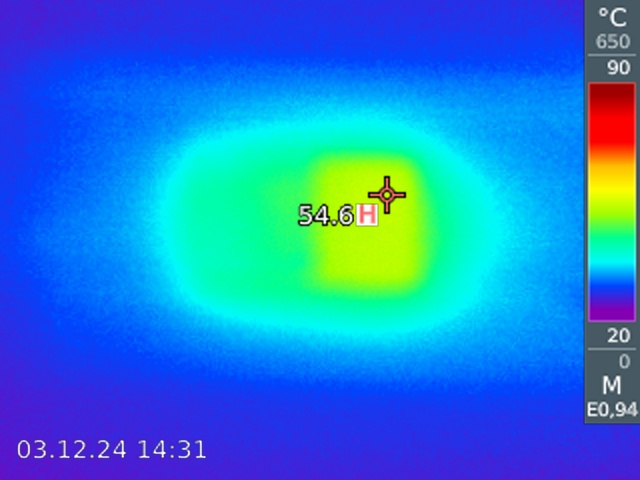

Supplement: S2 Figs — (ZIP) [file pone.0338325.s002.zip › image series/3. HA gel/TR004733.JPG]

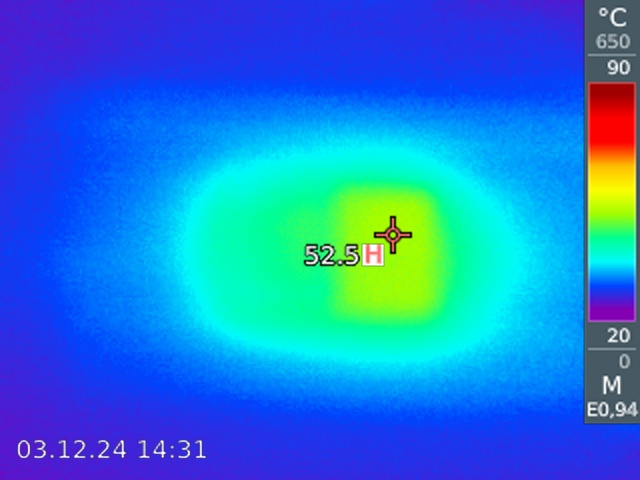

Supplement: S2 Figs — (ZIP) [file pone.0338325.s002.zip › image series/3. HA gel/TR004734.JPG]

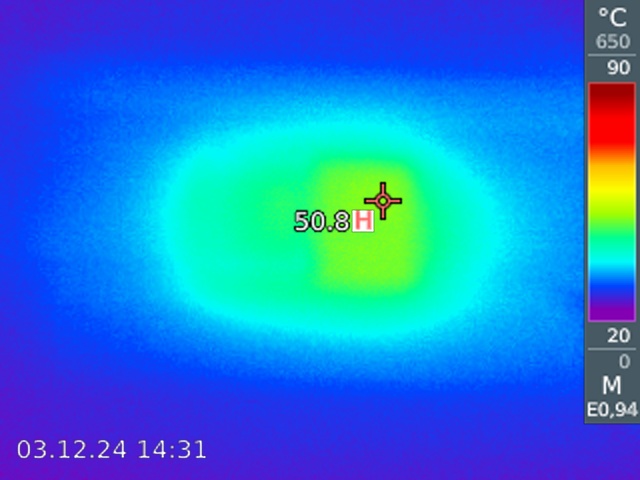

Supplement: S2 Figs — (ZIP) [file pone.0338325.s002.zip › image series/3. HA gel/TR004735.JPG]

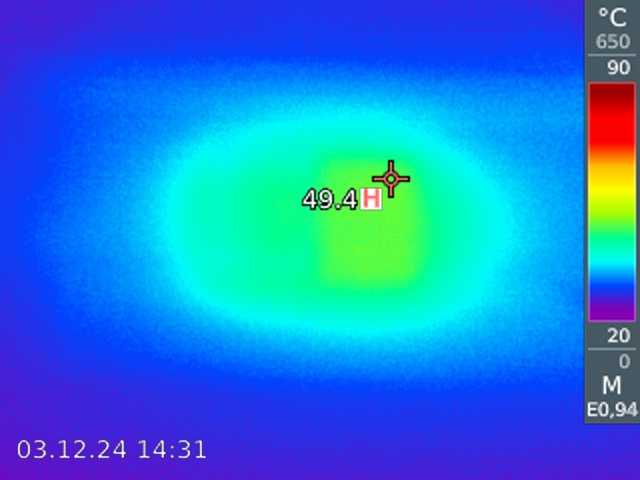

Supplement: S2 Figs — (ZIP) [file pone.0338325.s002.zip › image series/3. HA gel/TR004736.JPG]

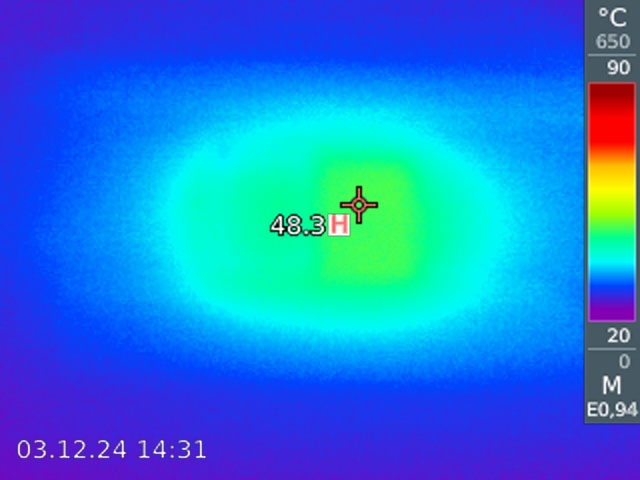

Supplement: S2 Figs — (ZIP) [file pone.0338325.s002.zip › image series/3. HA gel/TR004737.JPG]

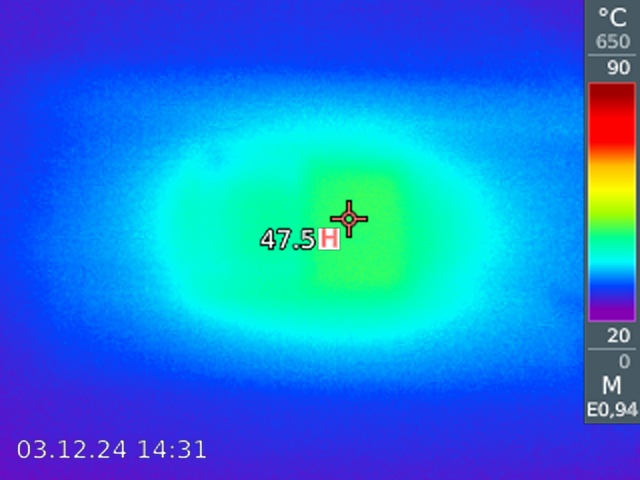

Supplement: S2 Figs — (ZIP) [file pone.0338325.s002.zip › image series/3. HA gel/TR004738.JPG]

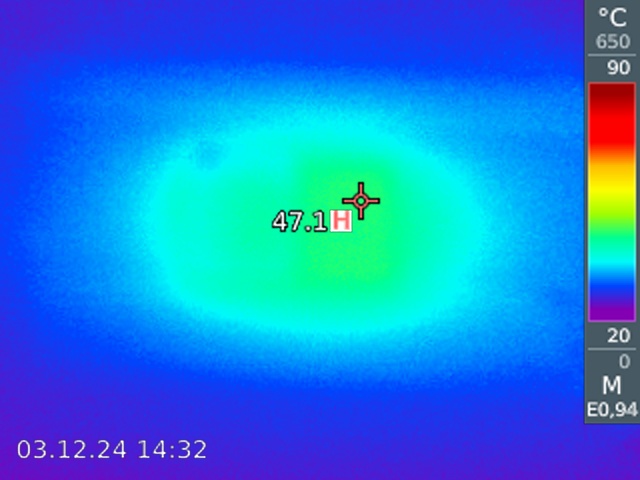

Supplement: S2 Figs — (ZIP) [file pone.0338325.s002.zip › image series/3. HA gel/TR004739.JPG]

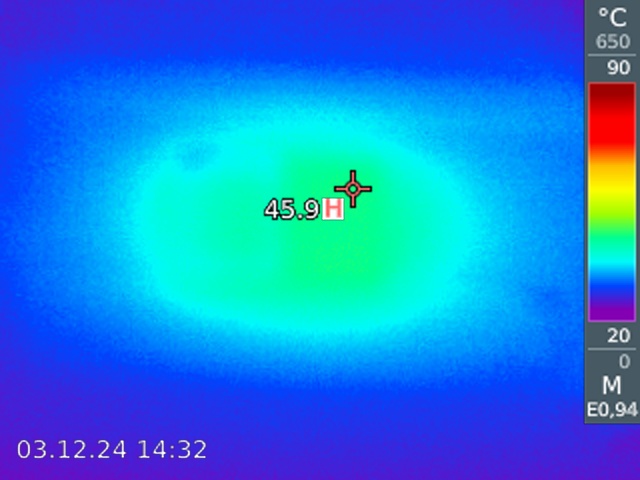

Supplement: S2 Figs — (ZIP) [file pone.0338325.s002.zip › image series/3. HA gel/TR004740.JPG]

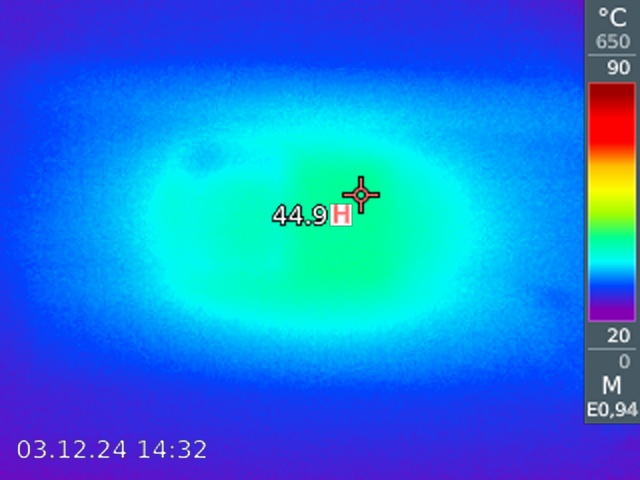

Supplement: S2 Figs — (ZIP) [file pone.0338325.s002.zip › image series/3. HA gel/TR004741.JPG]

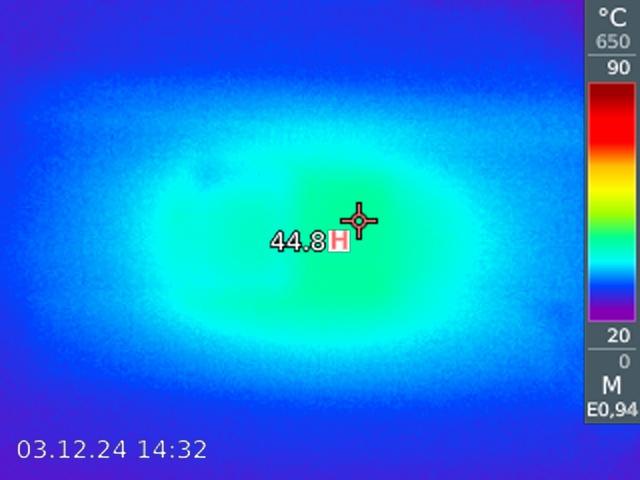

Supplement: S2 Figs — (ZIP) [file pone.0338325.s002.zip › image series/3. HA gel/TR004742.JPG]

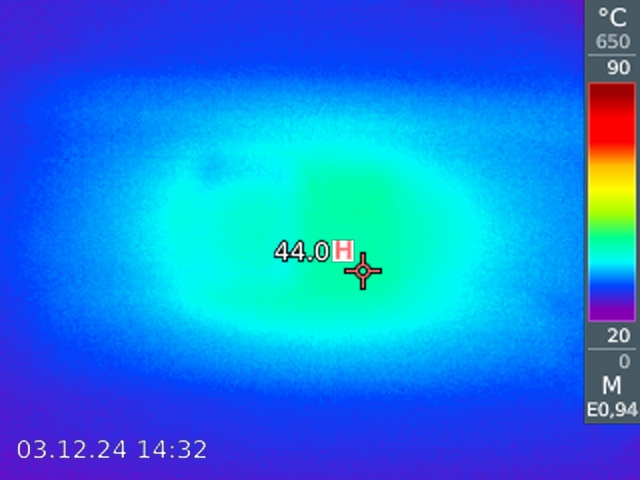

Supplement: S2 Figs — (ZIP) [file pone.0338325.s002.zip › image series/3. HA gel/TR004743.JPG]

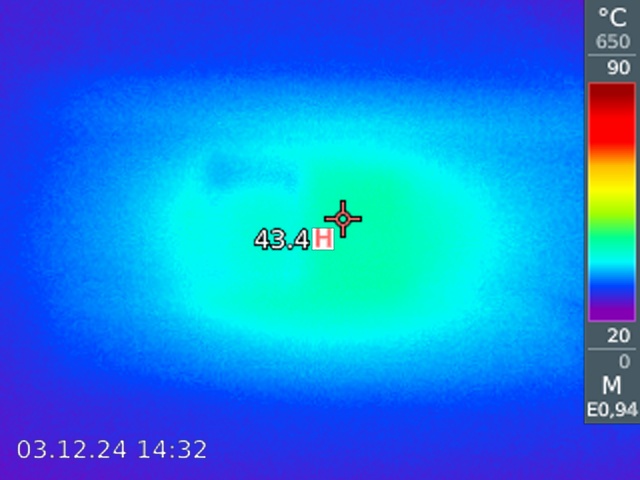

Supplement: S2 Figs — (ZIP) [file pone.0338325.s002.zip › image series/3. HA gel/TR004744.JPG]

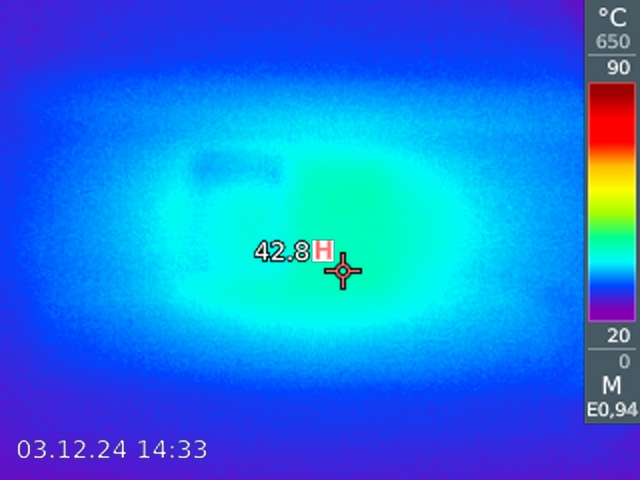

Supplement: S2 Figs — (ZIP) [file pone.0338325.s002.zip › image series/3. HA gel/TR004745.JPG]

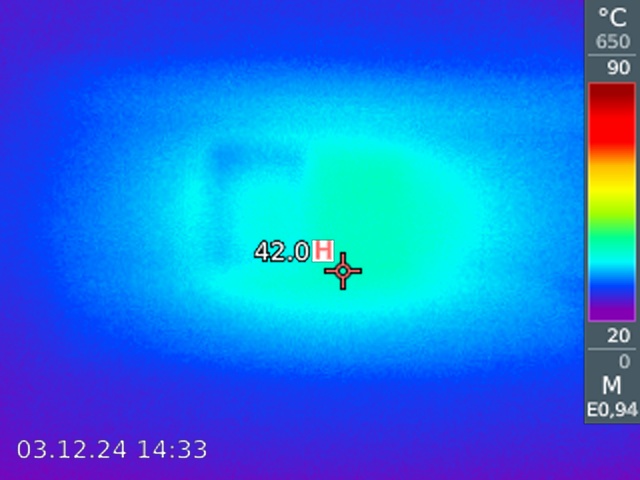

Supplement: S2 Figs — (ZIP) [file pone.0338325.s002.zip › image series/3. HA gel/TR004746.JPG]

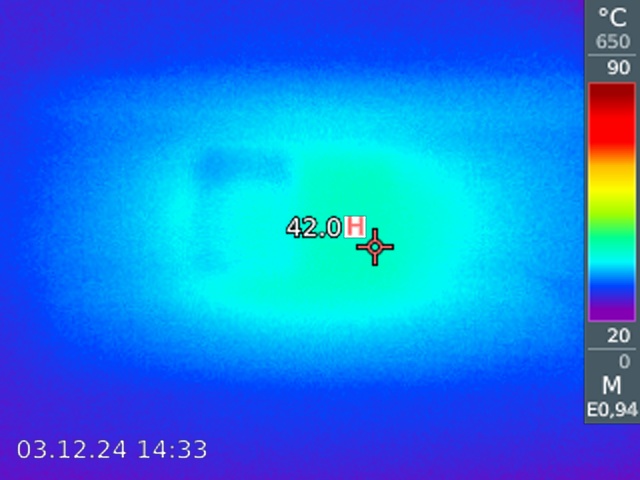

Supplement: S2 Figs — (ZIP) [file pone.0338325.s002.zip › image series/3. HA gel/TR004747.JPG]

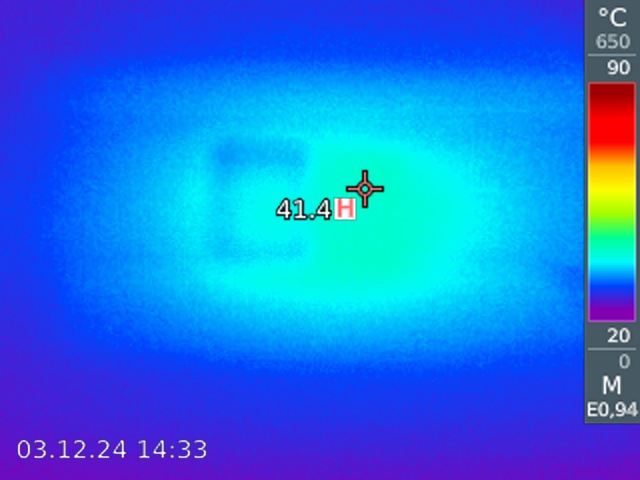

Supplement: S2 Figs — (ZIP) [file pone.0338325.s002.zip › image series/3. HA gel/TR004748.JPG]

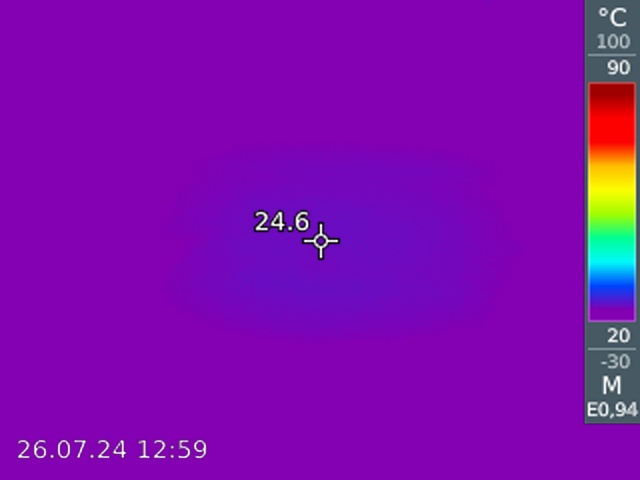

Supplement: S2 Figs — (ZIP) [file pone.0338325.s002.zip › image series/4. 1mm/TR004402.JPG]

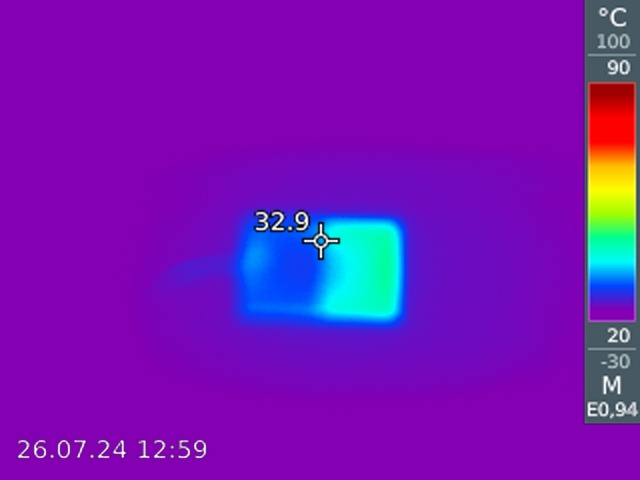

Supplement: S2 Figs — (ZIP) [file pone.0338325.s002.zip › image series/4. 1mm/TR004403.JPG]

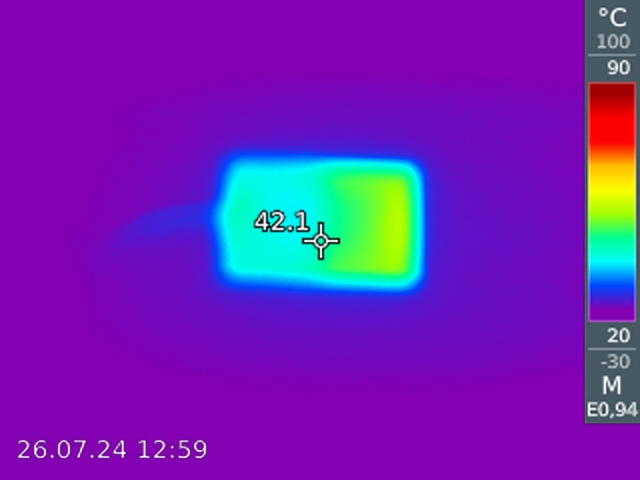

Supplement: S2 Figs — (ZIP) [file pone.0338325.s002.zip › image series/4. 1mm/TR004404.JPG]

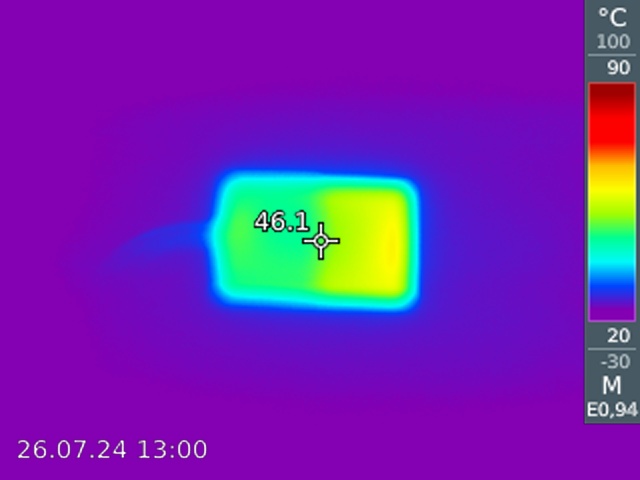

Supplement: S2 Figs — (ZIP) [file pone.0338325.s002.zip › image series/4. 1mm/TR004405.JPG]

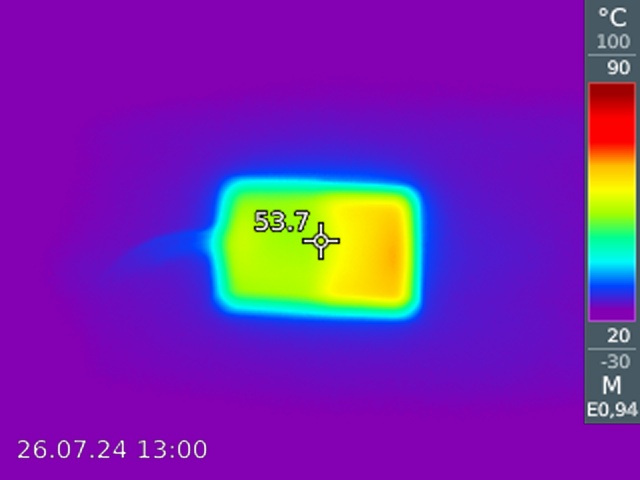

Supplement: S2 Figs — (ZIP) [file pone.0338325.s002.zip › image series/4. 1mm/TR004406.JPG]

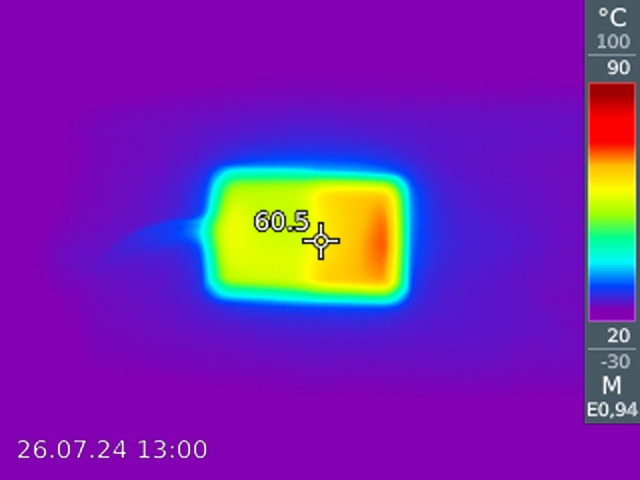

Supplement: S2 Figs — (ZIP) [file pone.0338325.s002.zip › image series/4. 1mm/TR004407.JPG]

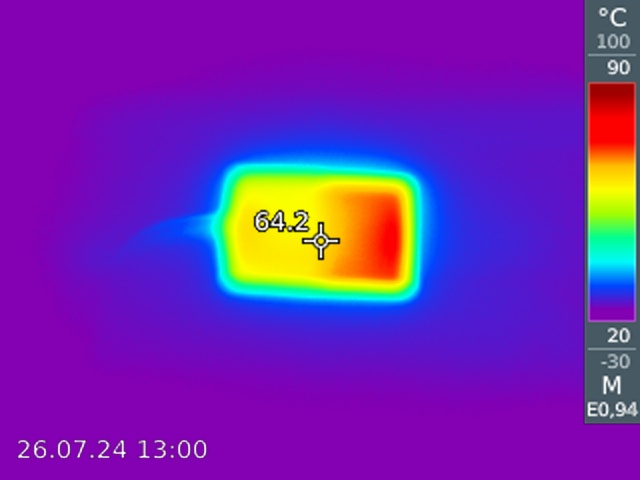

Supplement: S2 Figs — (ZIP) [file pone.0338325.s002.zip › image series/4. 1mm/TR004408.JPG]

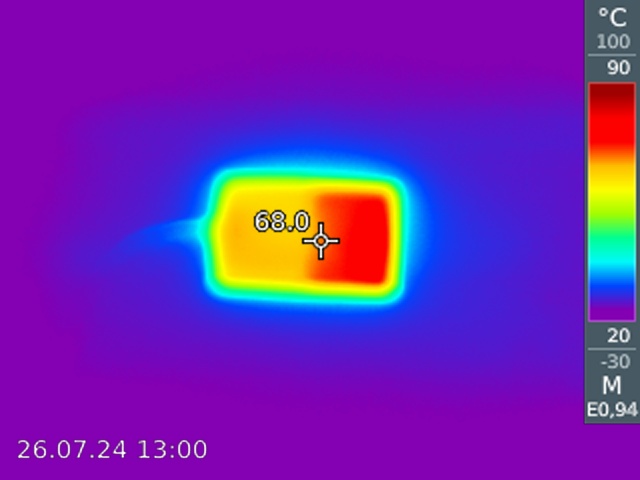

Supplement: S2 Figs — (ZIP) [file pone.0338325.s002.zip › image series/4. 1mm/TR004409.JPG]

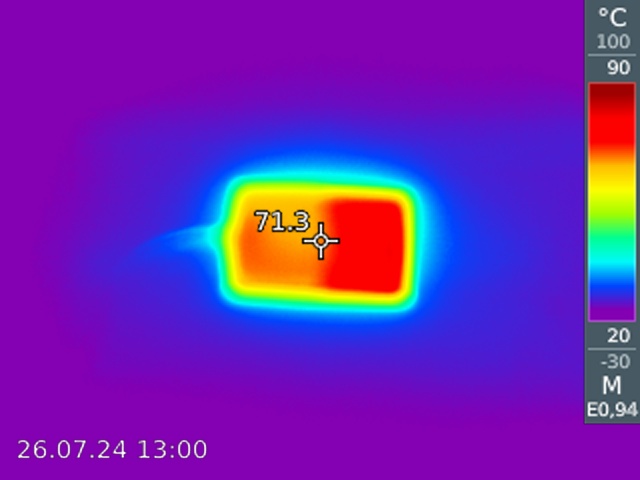

Supplement: S2 Figs — (ZIP) [file pone.0338325.s002.zip › image series/4. 1mm/TR004410.JPG]

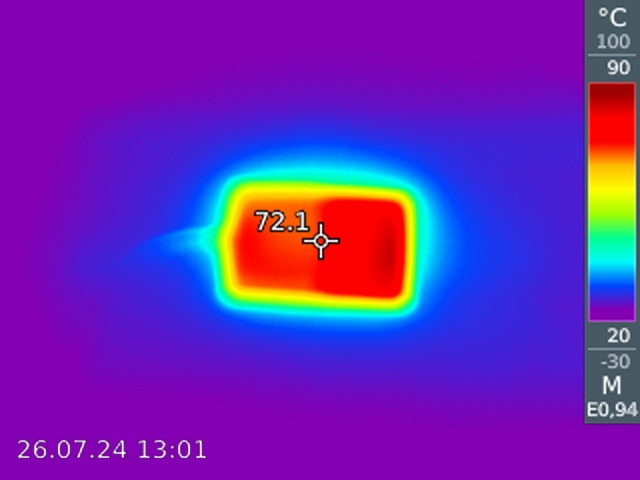

Supplement: S2 Figs — (ZIP) [file pone.0338325.s002.zip › image series/4. 1mm/TR004411.JPG]

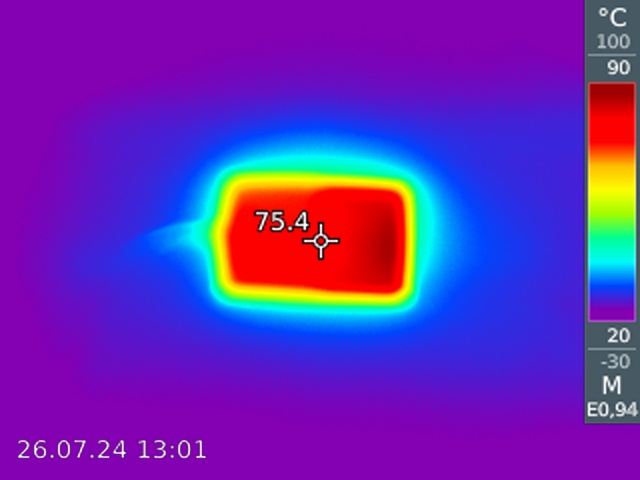

Supplement: S2 Figs — (ZIP) [file pone.0338325.s002.zip › image series/4. 1mm/TR004412.JPG]

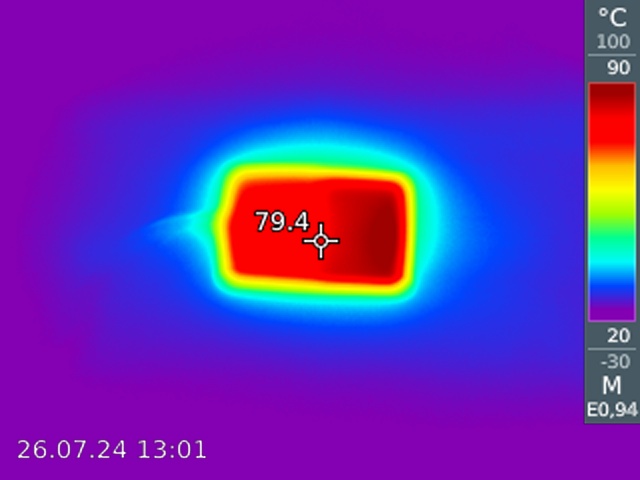

Supplement: S2 Figs — (ZIP) [file pone.0338325.s002.zip › image series/4. 1mm/TR004413.JPG]

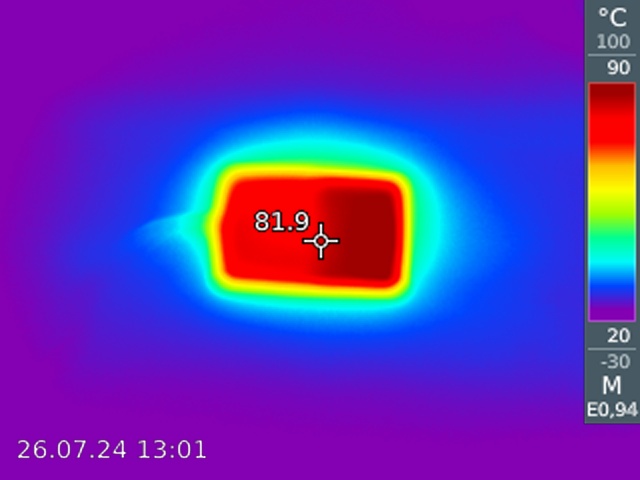

Supplement: S2 Figs — (ZIP) [file pone.0338325.s002.zip › image series/4. 1mm/TR004414.JPG]

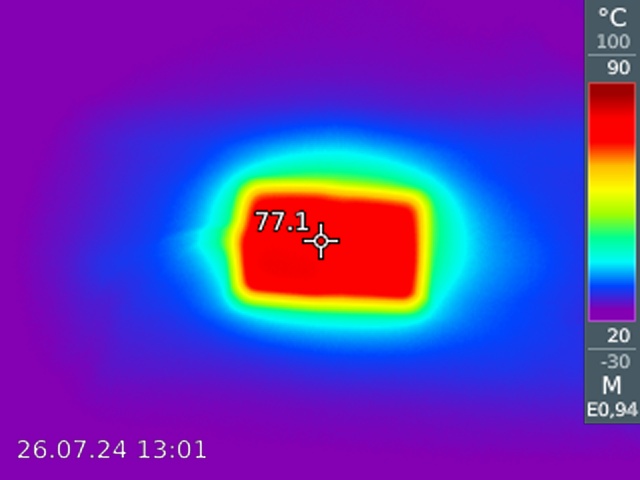

Supplement: S2 Figs — (ZIP) [file pone.0338325.s002.zip › image series/4. 1mm/TR004415.JPG]

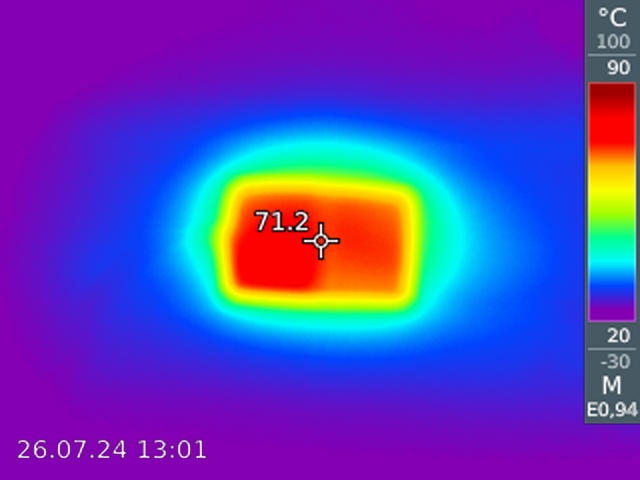

Supplement: S2 Figs — (ZIP) [file pone.0338325.s002.zip › image series/4. 1mm/TR004416.JPG]

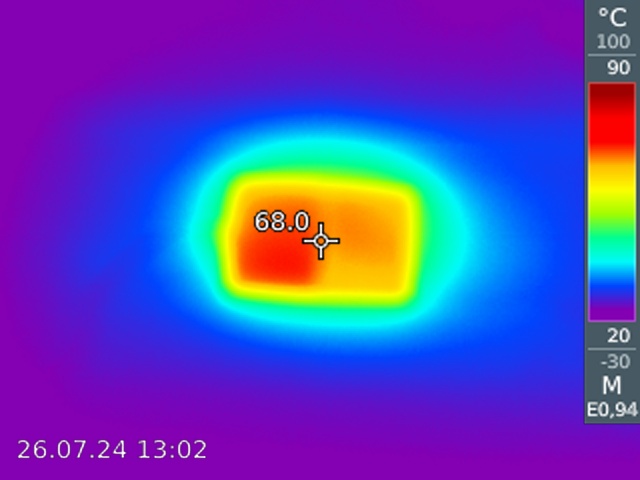

Supplement: S2 Figs — (ZIP) [file pone.0338325.s002.zip › image series/4. 1mm/TR004417.JPG]

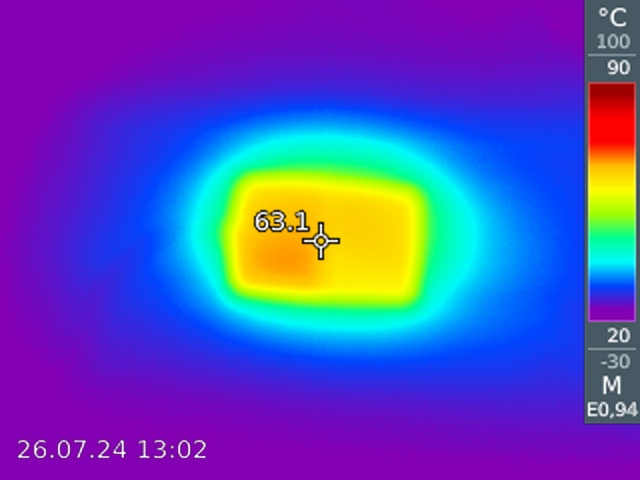

Supplement: S2 Figs — (ZIP) [file pone.0338325.s002.zip › image series/4. 1mm/TR004418.JPG]

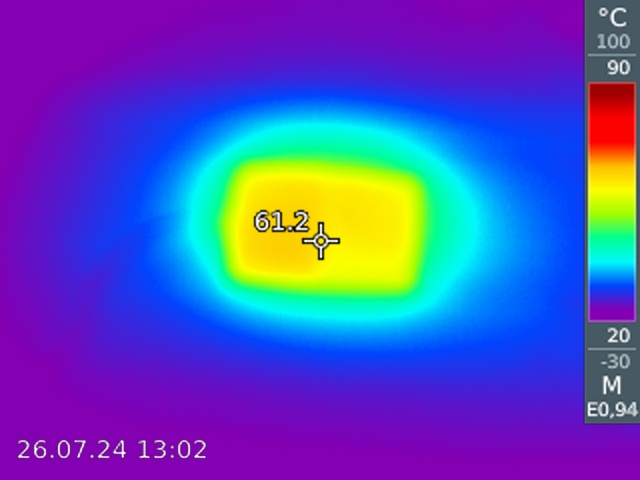

Supplement: S2 Figs — (ZIP) [file pone.0338325.s002.zip › image series/4. 1mm/TR004419.JPG]

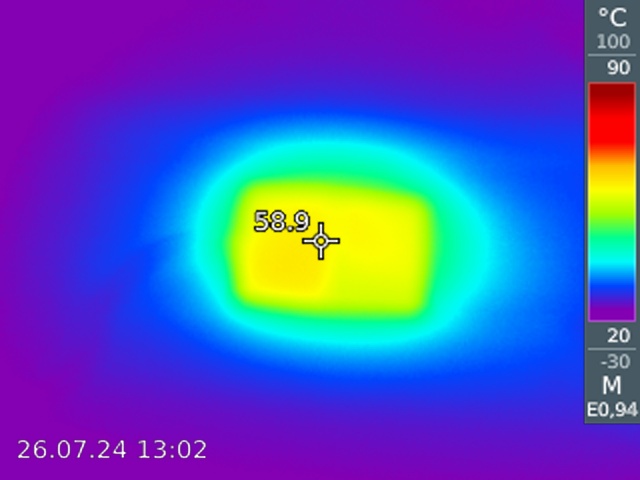

Supplement: S2 Figs — (ZIP) [file pone.0338325.s002.zip › image series/4. 1mm/TR004420.JPG]

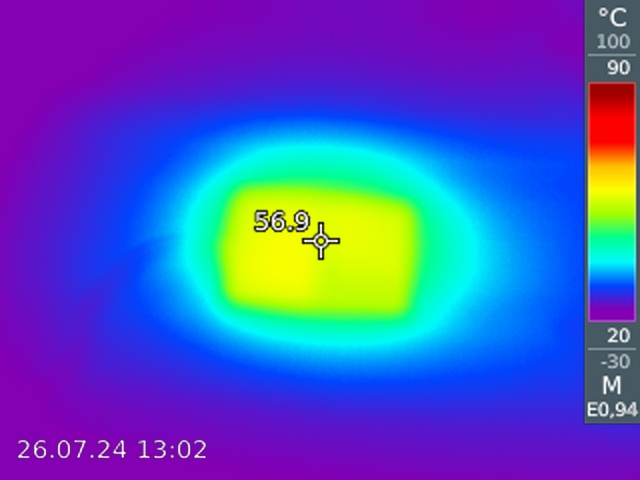

Supplement: S2 Figs — (ZIP) [file pone.0338325.s002.zip › image series/4. 1mm/TR004421.JPG]

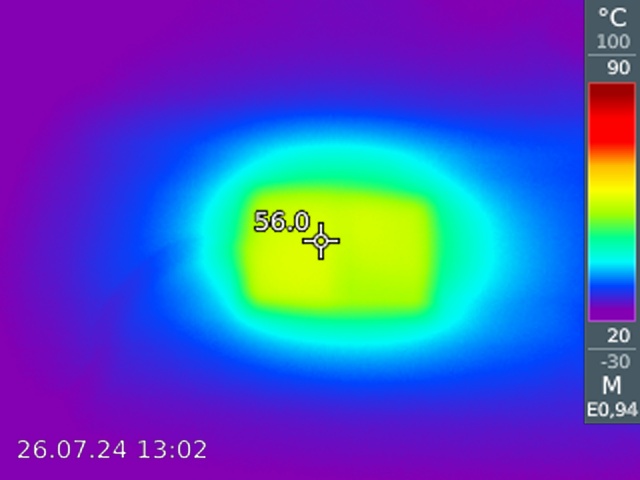

Supplement: S2 Figs — (ZIP) [file pone.0338325.s002.zip › image series/4. 1mm/TR004422.JPG]

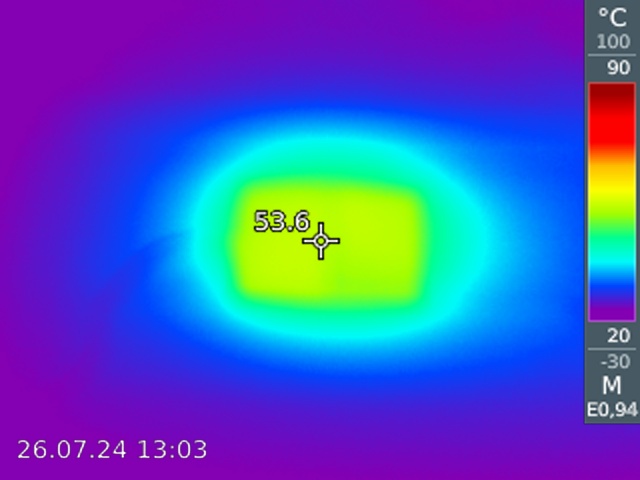

Supplement: S2 Figs — (ZIP) [file pone.0338325.s002.zip › image series/4. 1mm/TR004423.JPG]

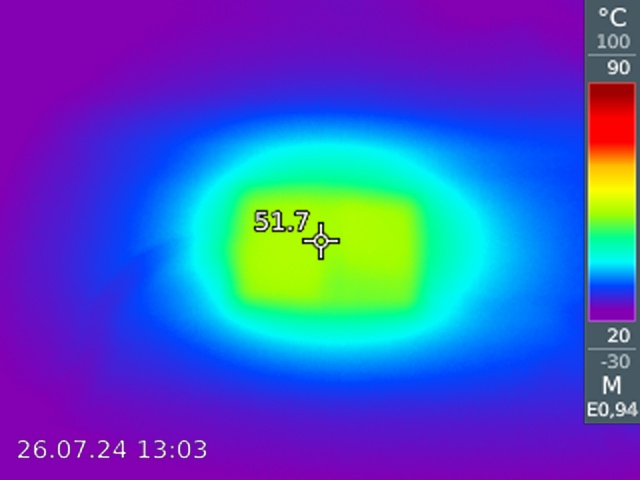

Supplement: S2 Figs — (ZIP) [file pone.0338325.s002.zip › image series/4. 1mm/TR004424.JPG]

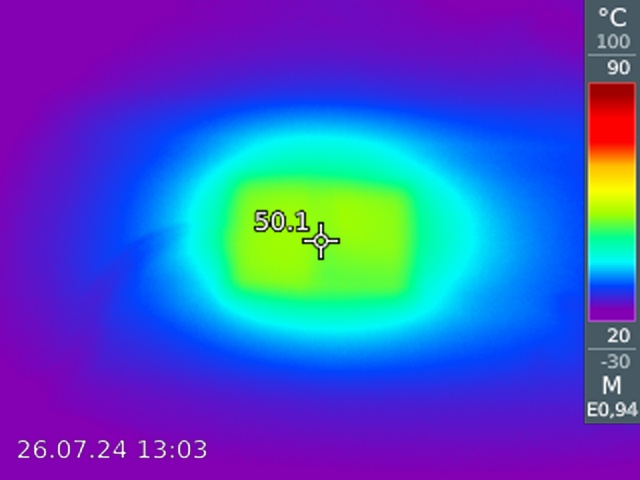

Supplement: S2 Figs — (ZIP) [file pone.0338325.s002.zip › image series/4. 1mm/TR004425.JPG]

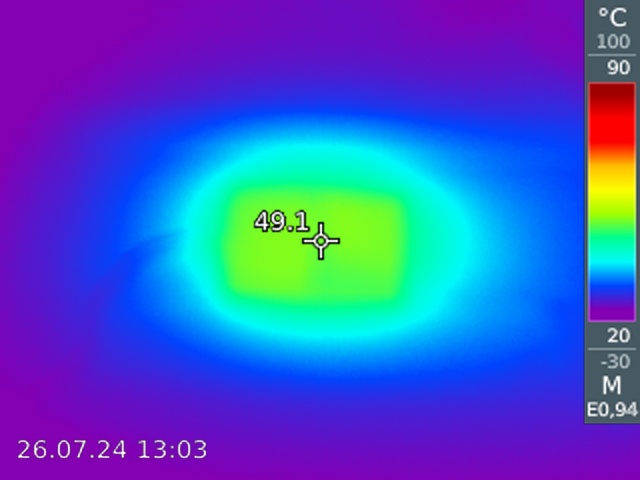

Supplement: S2 Figs — (ZIP) [file pone.0338325.s002.zip › image series/4. 1mm/TR004426.JPG]

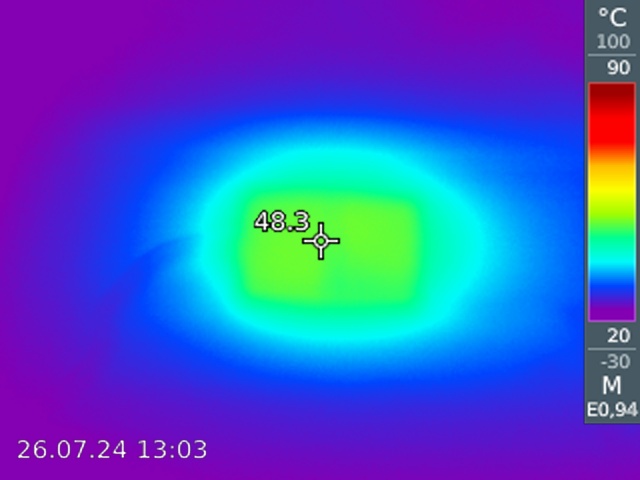

Supplement: S2 Figs — (ZIP) [file pone.0338325.s002.zip › image series/4. 1mm/TR004427.JPG]

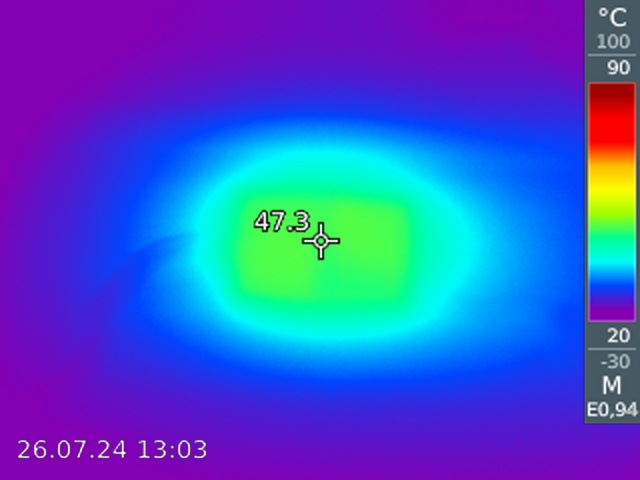

Supplement: S2 Figs — (ZIP) [file pone.0338325.s002.zip › image series/4. 1mm/TR004428.JPG]

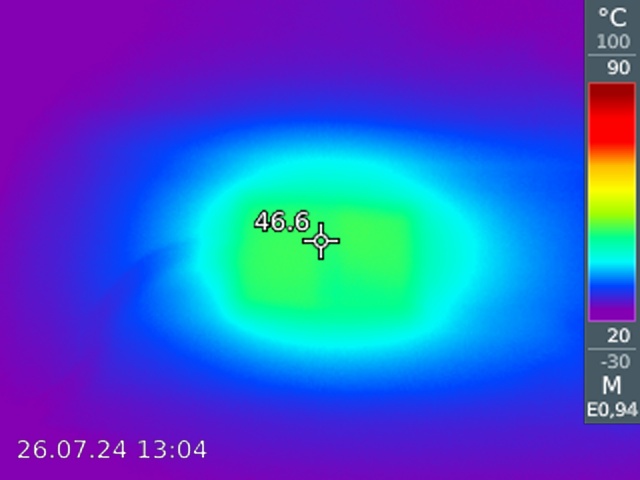

Supplement: S2 Figs — (ZIP) [file pone.0338325.s002.zip › image series/4. 1mm/TR004429.JPG]

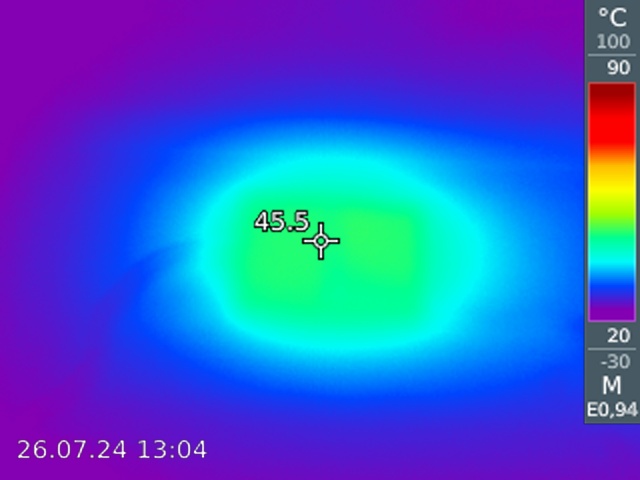

Supplement: S2 Figs — (ZIP) [file pone.0338325.s002.zip › image series/4. 1mm/TR004430.JPG]

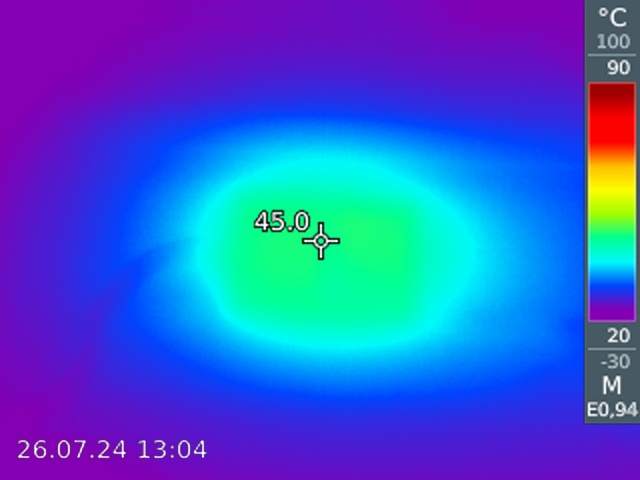

Supplement: S2 Figs — (ZIP) [file pone.0338325.s002.zip › image series/4. 1mm/TR004431.JPG]

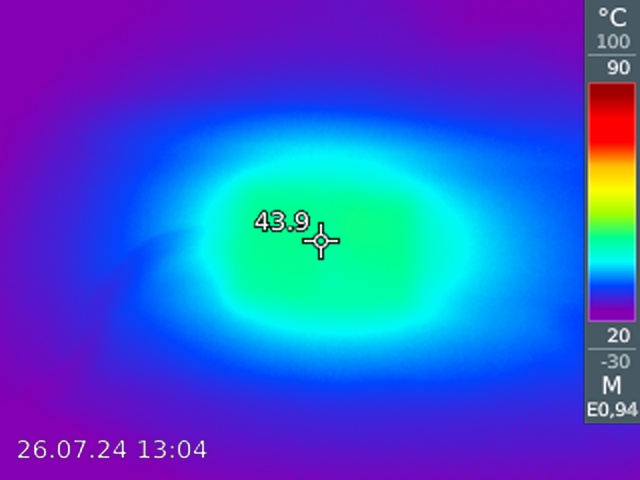

Supplement: S2 Figs — (ZIP) [file pone.0338325.s002.zip › image series/4. 1mm/TR004432.JPG]

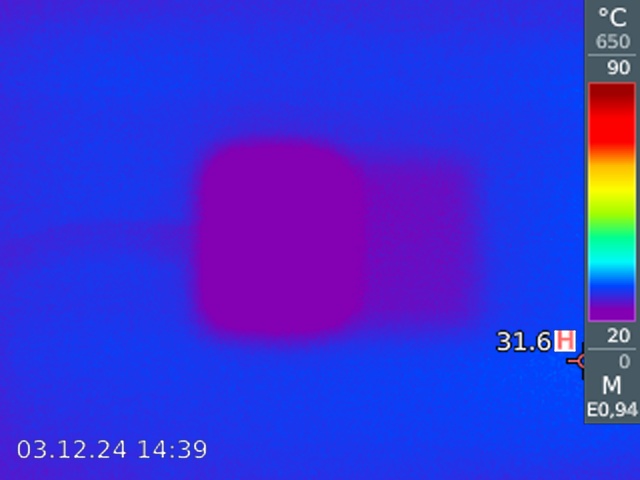

Supplement: S2 Figs — (ZIP) [file pone.0338325.s002.zip › image series/5. 1mm gel/TR004749.JPG]

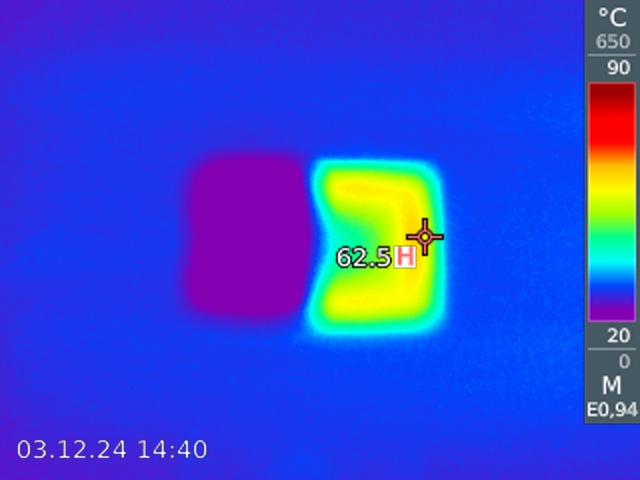

Supplement: S2 Figs — (ZIP) [file pone.0338325.s002.zip › image series/5. 1mm gel/TR004750.JPG]

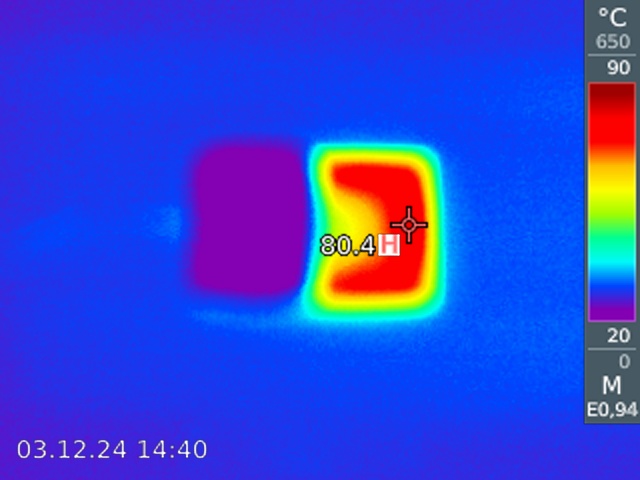

Supplement: S2 Figs — (ZIP) [file pone.0338325.s002.zip › image series/5. 1mm gel/TR004751.JPG]

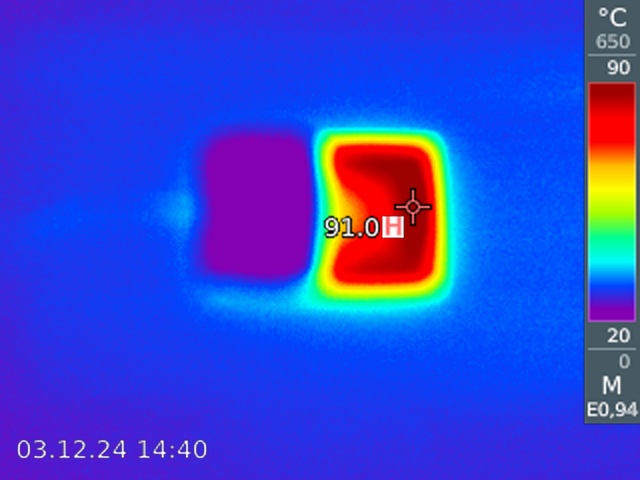

Supplement: S2 Figs — (ZIP) [file pone.0338325.s002.zip › image series/5. 1mm gel/TR004752.JPG]

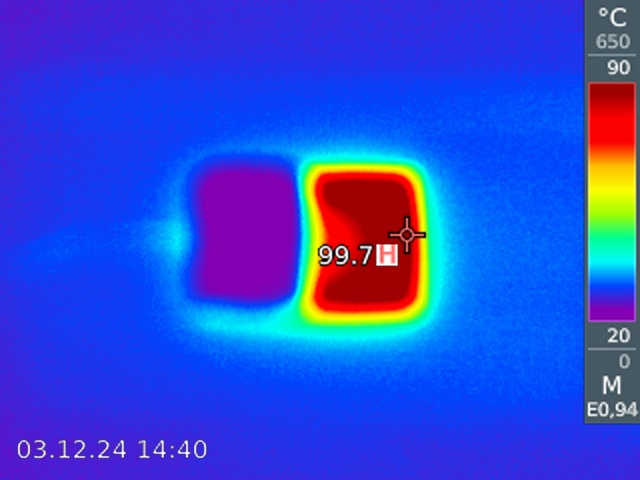

Supplement: S2 Figs — (ZIP) [file pone.0338325.s002.zip › image series/5. 1mm gel/TR004753.JPG]

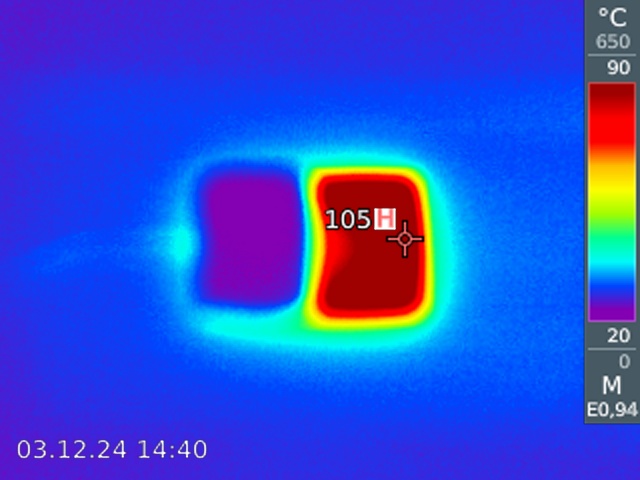

Supplement: S2 Figs — (ZIP) [file pone.0338325.s002.zip › image series/5. 1mm gel/TR004754.JPG]

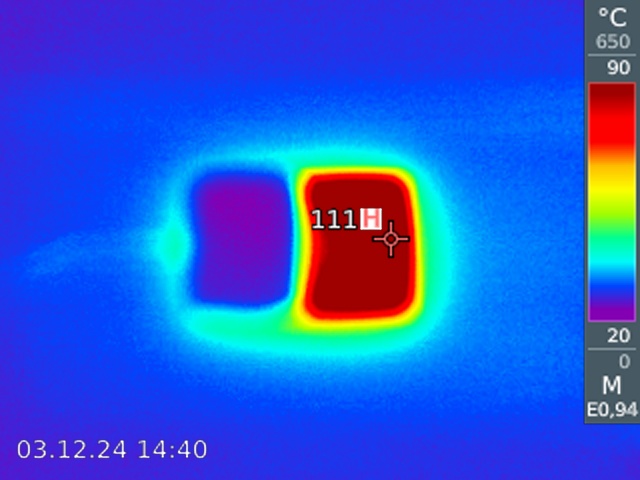

Supplement: S2 Figs — (ZIP) [file pone.0338325.s002.zip › image series/5. 1mm gel/TR004755.JPG]

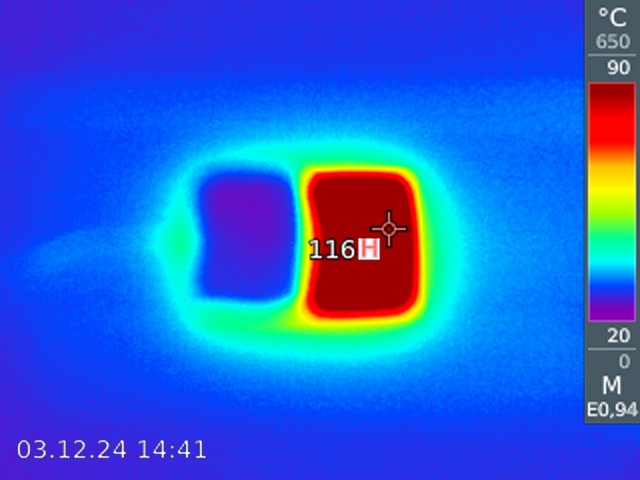

Supplement: S2 Figs — (ZIP) [file pone.0338325.s002.zip › image series/5. 1mm gel/TR004756.JPG]

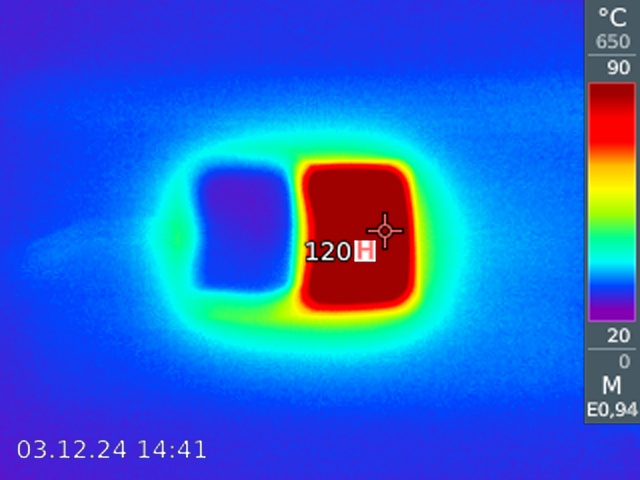

Supplement: S2 Figs — (ZIP) [file pone.0338325.s002.zip › image series/5. 1mm gel/TR004757.JPG]

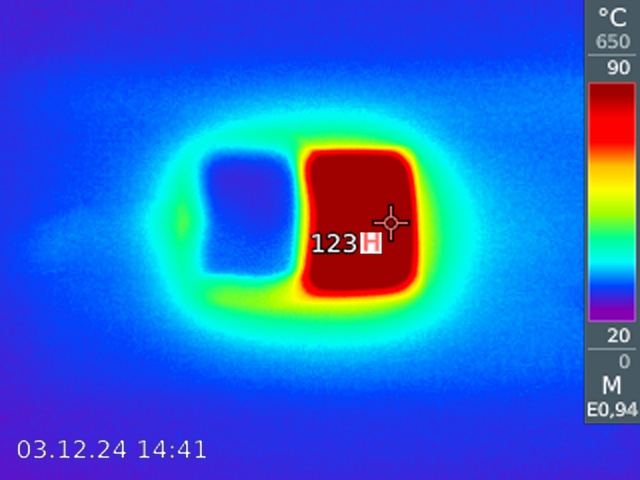

Supplement: S2 Figs — (ZIP) [file pone.0338325.s002.zip › image series/5. 1mm gel/TR004758.JPG]

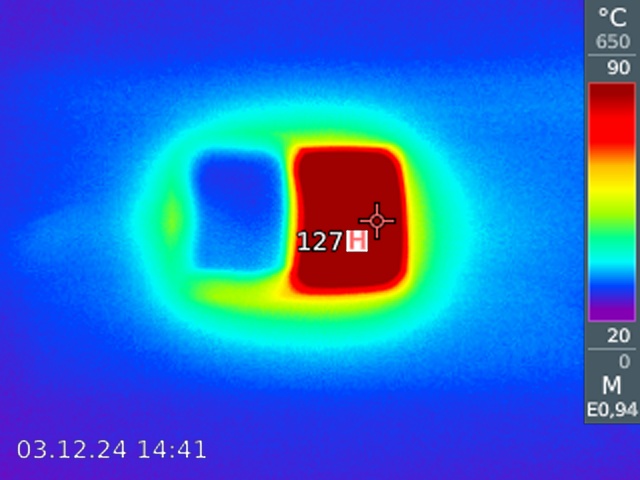

Supplement: S2 Figs — (ZIP) [file pone.0338325.s002.zip › image series/5. 1mm gel/TR004759.JPG]

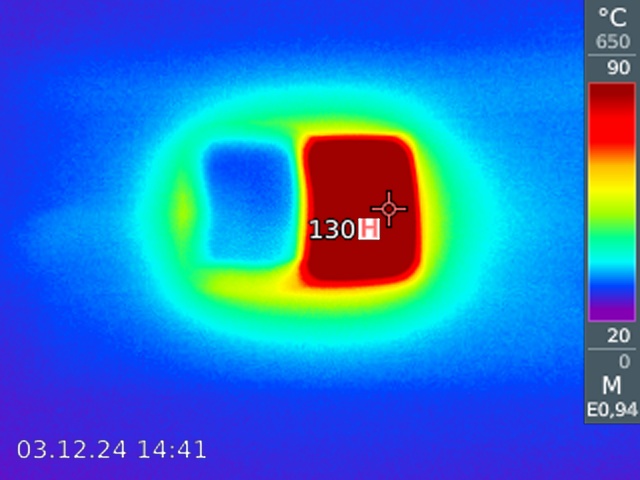

Supplement: S2 Figs — (ZIP) [file pone.0338325.s002.zip › image series/5. 1mm gel/TR004760.JPG]

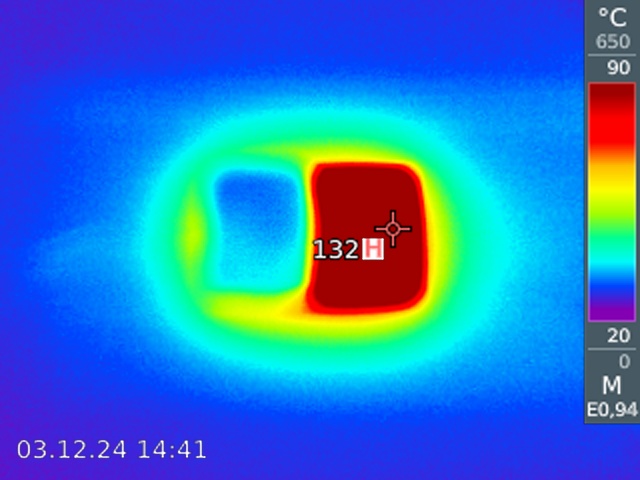

Supplement: S2 Figs — (ZIP) [file pone.0338325.s002.zip › image series/5. 1mm gel/TR004761.JPG]

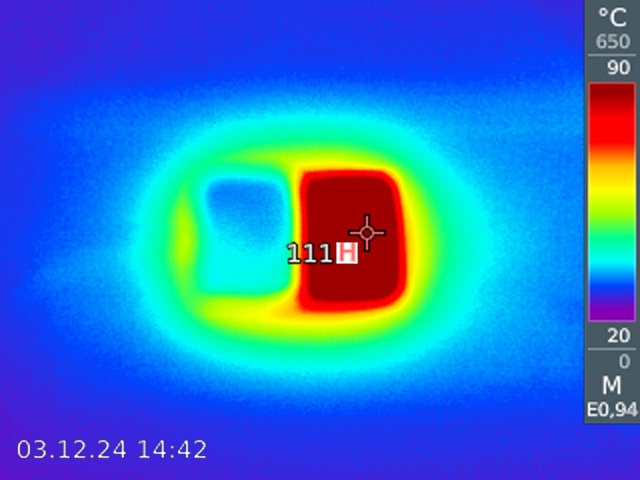

Supplement: S2 Figs — (ZIP) [file pone.0338325.s002.zip › image series/5. 1mm gel/TR004762.JPG]

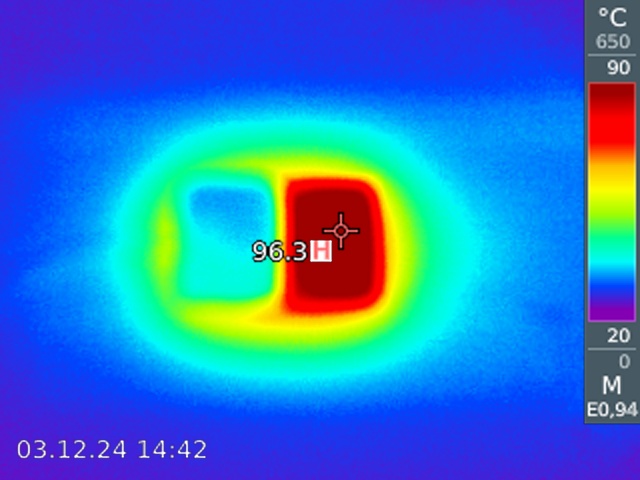

Supplement: S2 Figs — (ZIP) [file pone.0338325.s002.zip › image series/5. 1mm gel/TR004763.JPG]

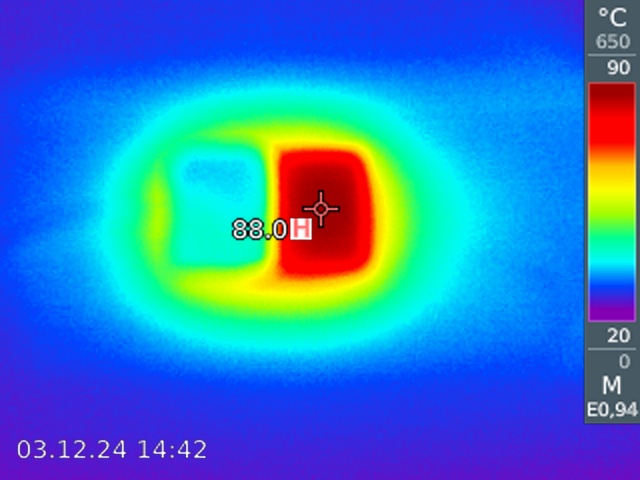

Supplement: S2 Figs — (ZIP) [file pone.0338325.s002.zip › image series/5. 1mm gel/TR004764.JPG]

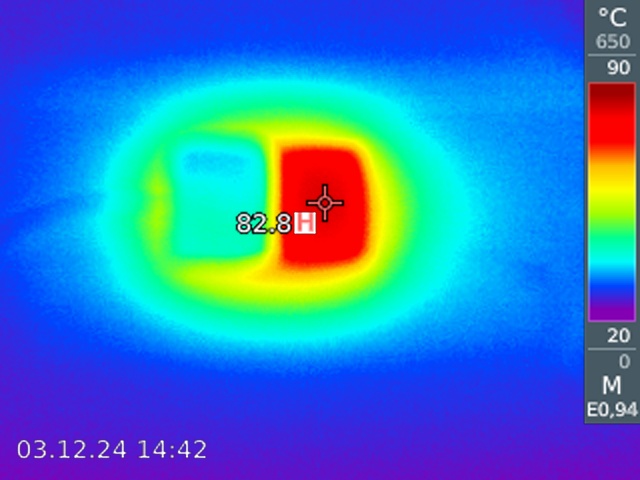

Supplement: S2 Figs — (ZIP) [file pone.0338325.s002.zip › image series/5. 1mm gel/TR004765.JPG]

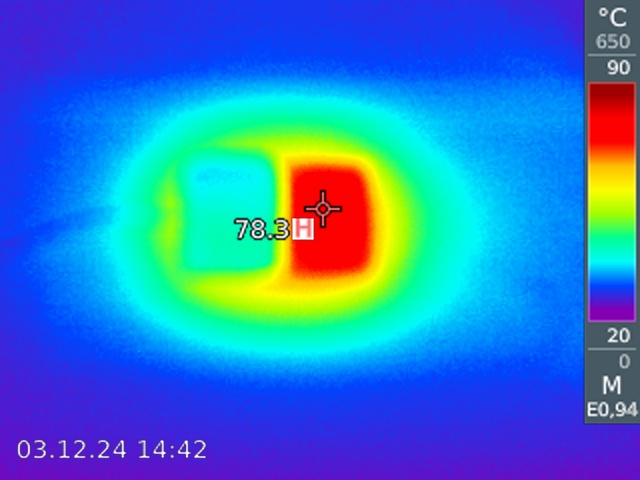

Supplement: S2 Figs — (ZIP) [file pone.0338325.s002.zip › image series/5. 1mm gel/TR004766.JPG]

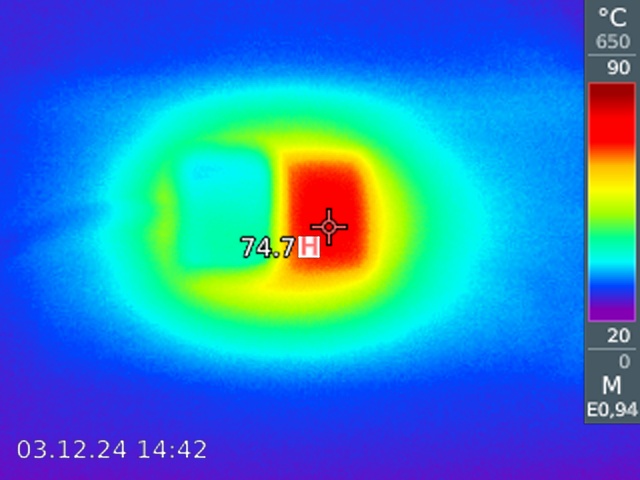

Supplement: S2 Figs — (ZIP) [file pone.0338325.s002.zip › image series/5. 1mm gel/TR004767.JPG]

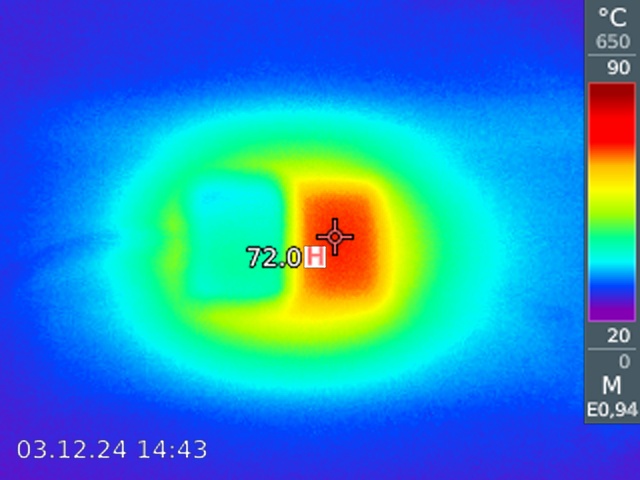

Supplement: S2 Figs — (ZIP) [file pone.0338325.s002.zip › image series/5. 1mm gel/TR004768.JPG]

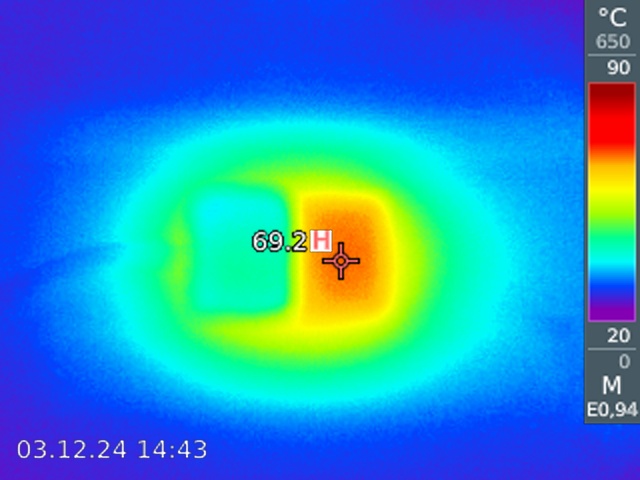

Supplement: S2 Figs — (ZIP) [file pone.0338325.s002.zip › image series/5. 1mm gel/TR004769.JPG]

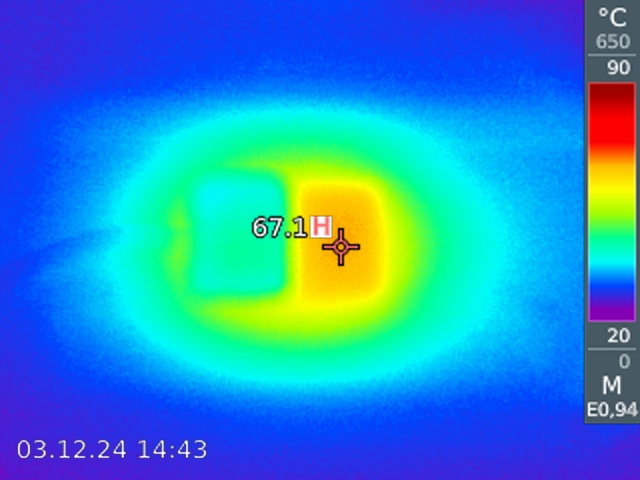

Supplement: S2 Figs — (ZIP) [file pone.0338325.s002.zip › image series/5. 1mm gel/TR004770.JPG]

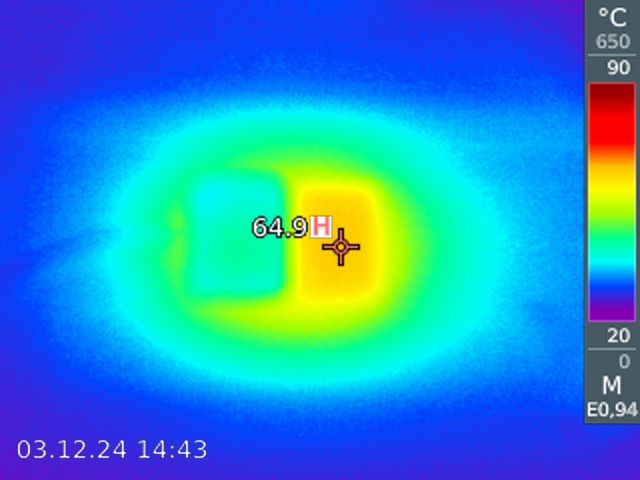

Supplement: S2 Figs — (ZIP) [file pone.0338325.s002.zip › image series/5. 1mm gel/TR004771.JPG]

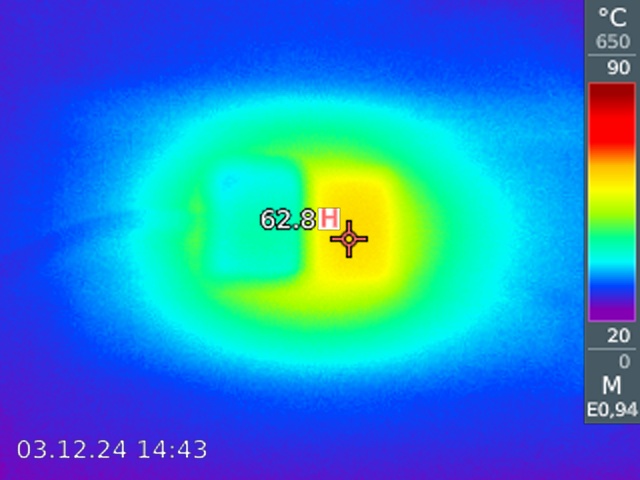

Supplement: S2 Figs — (ZIP) [file pone.0338325.s002.zip › image series/5. 1mm gel/TR004772.JPG]

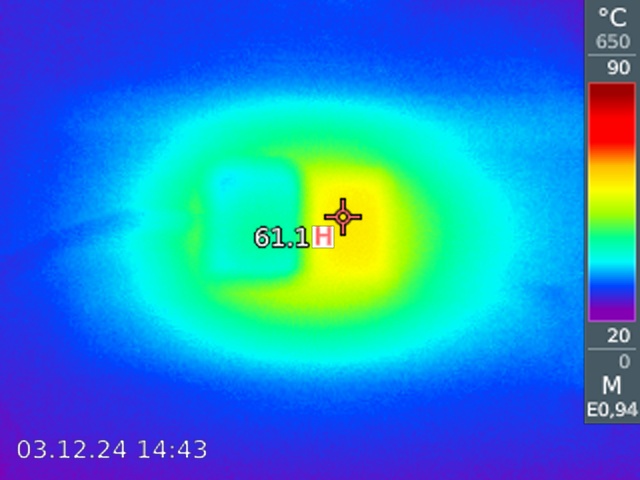

Supplement: S2 Figs — (ZIP) [file pone.0338325.s002.zip › image series/5. 1mm gel/TR004773.JPG]

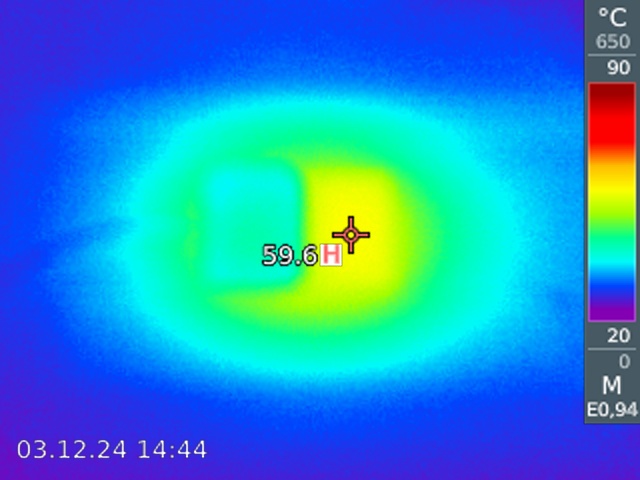

Supplement: S2 Figs — (ZIP) [file pone.0338325.s002.zip › image series/5. 1mm gel/TR004774.JPG]

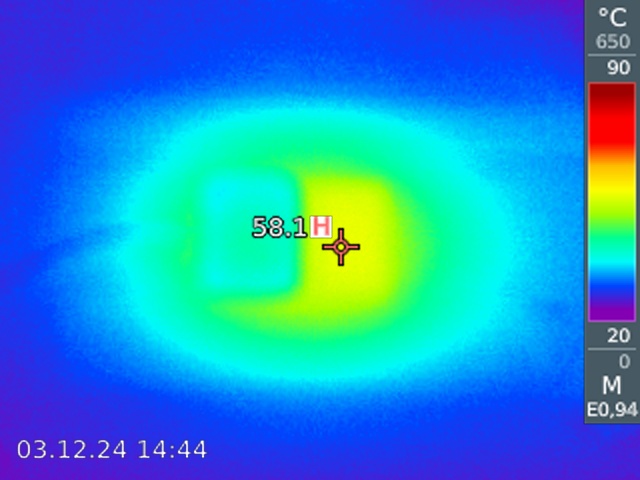

Supplement: S2 Figs — (ZIP) [file pone.0338325.s002.zip › image series/5. 1mm gel/TR004775.JPG]

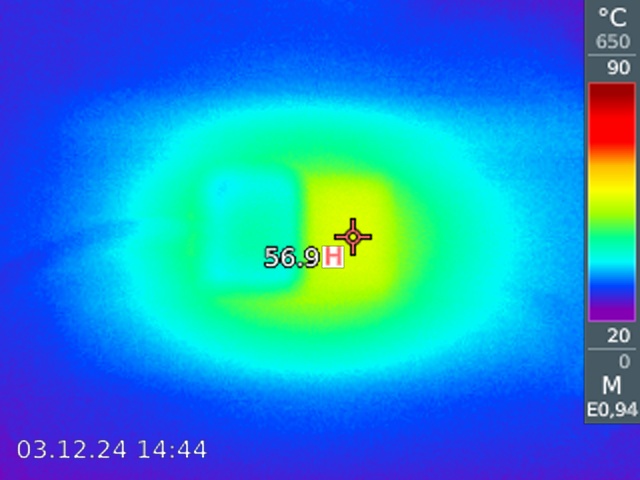

Supplement: S2 Figs — (ZIP) [file pone.0338325.s002.zip › image series/5. 1mm gel/TR004776.JPG]

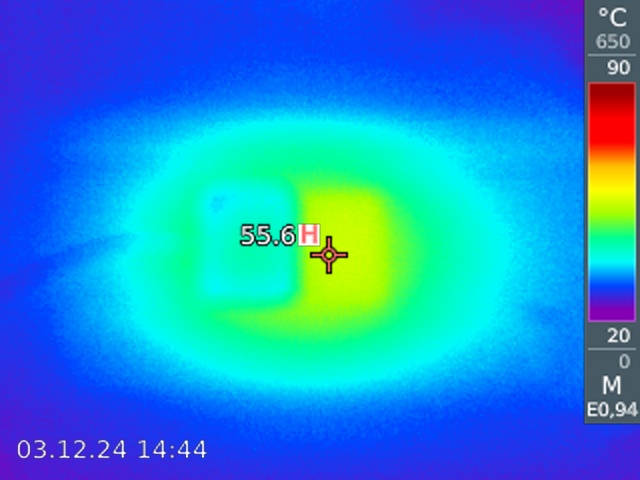

Supplement: S2 Figs — (ZIP) [file pone.0338325.s002.zip › image series/5. 1mm gel/TR004777.JPG]

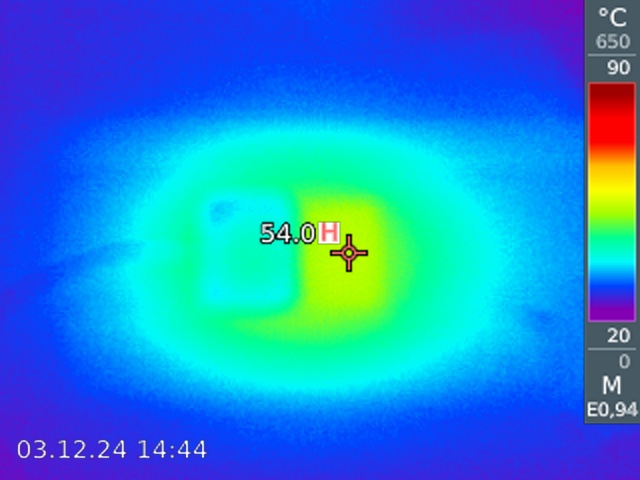

Supplement: S2 Figs — (ZIP) [file pone.0338325.s002.zip › image series/5. 1mm gel/TR004778.JPG]

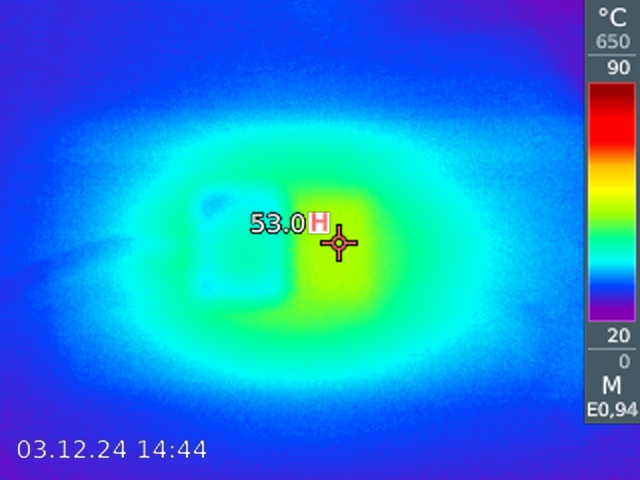

Supplement: S2 Figs — (ZIP) [file pone.0338325.s002.zip › image series/5. 1mm gel/TR004779.JPG]

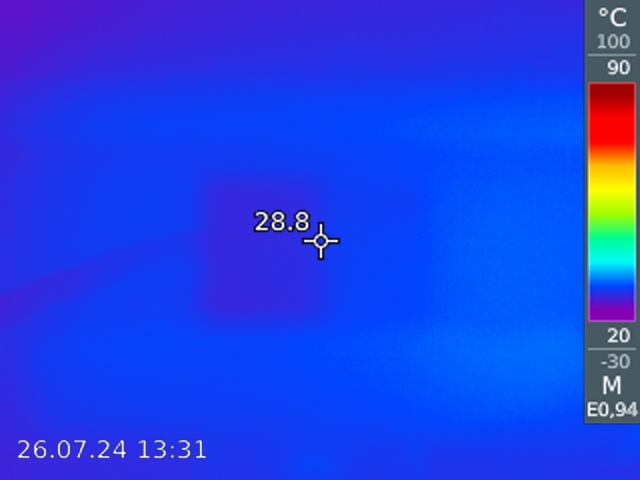

Supplement: S2 Figs — (ZIP) [file pone.0338325.s002.zip › image series/6. 3mm/TR004468.JPG]

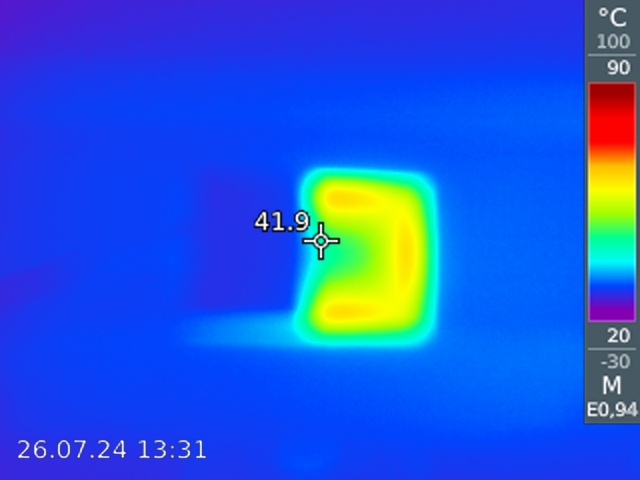

Supplement: S2 Figs — (ZIP) [file pone.0338325.s002.zip › image series/6. 3mm/TR004469.JPG]

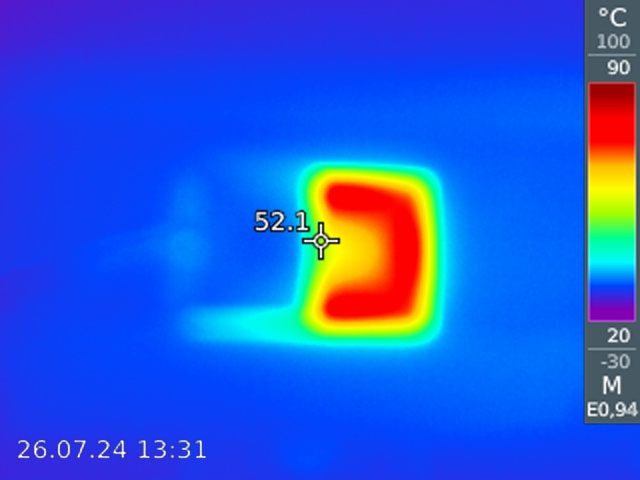

Supplement: S2 Figs — (ZIP) [file pone.0338325.s002.zip › image series/6. 3mm/TR004470.JPG]

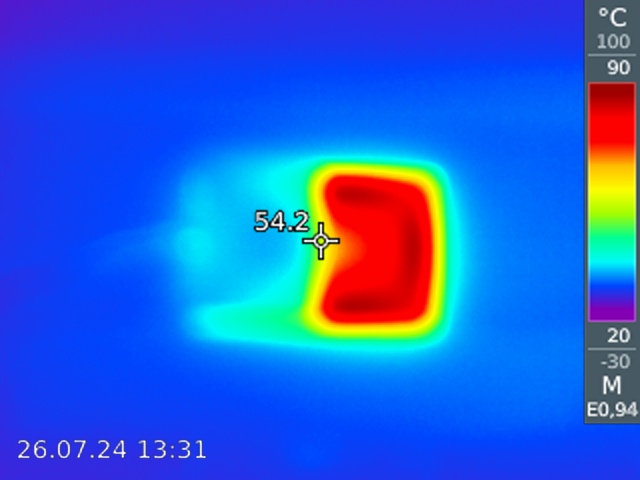

Supplement: S2 Figs — (ZIP) [file pone.0338325.s002.zip › image series/6. 3mm/TR004471.JPG]

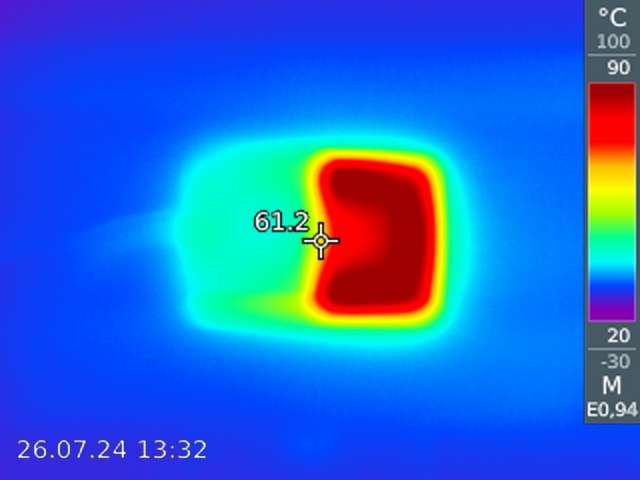

Supplement: S2 Figs — (ZIP) [file pone.0338325.s002.zip › image series/6. 3mm/TR004472.JPG]

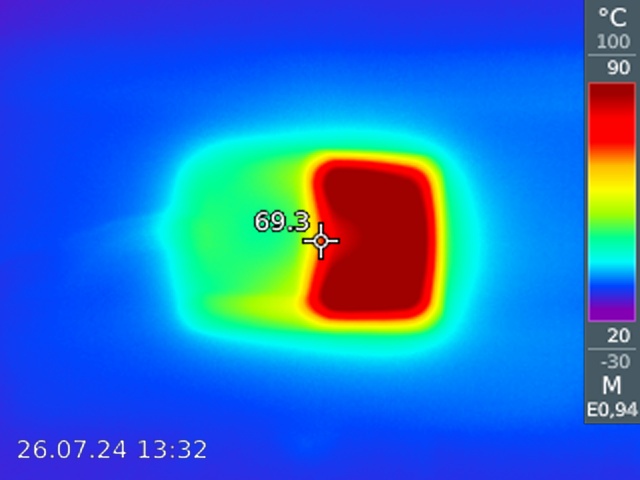

Supplement: S2 Figs — (ZIP) [file pone.0338325.s002.zip › image series/6. 3mm/TR004473.JPG]

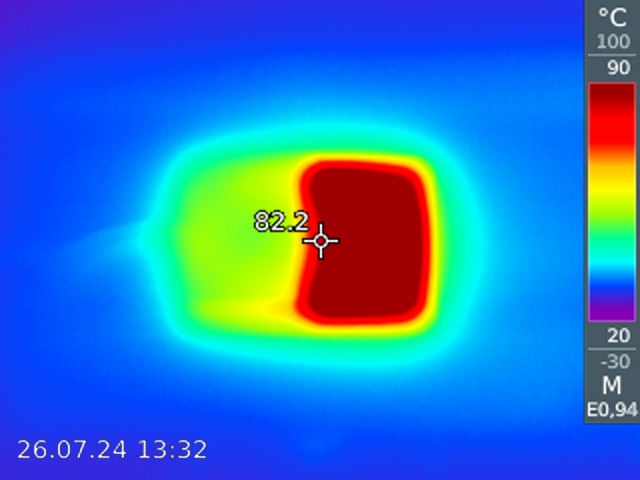

Supplement: S2 Figs — (ZIP) [file pone.0338325.s002.zip › image series/6. 3mm/TR004474.JPG]

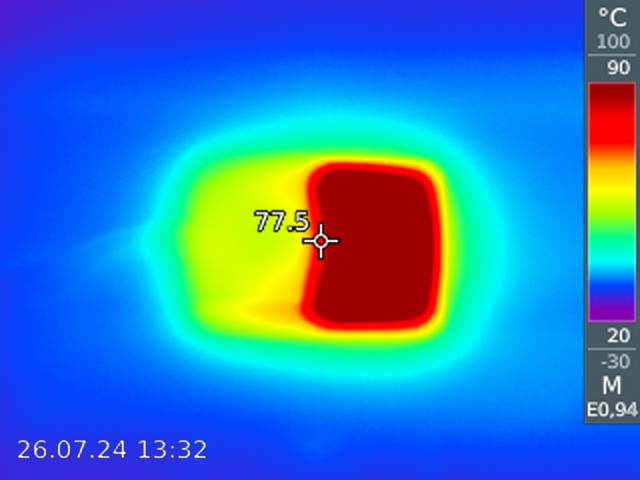

Supplement: S2 Figs — (ZIP) [file pone.0338325.s002.zip › image series/6. 3mm/TR004475.JPG]

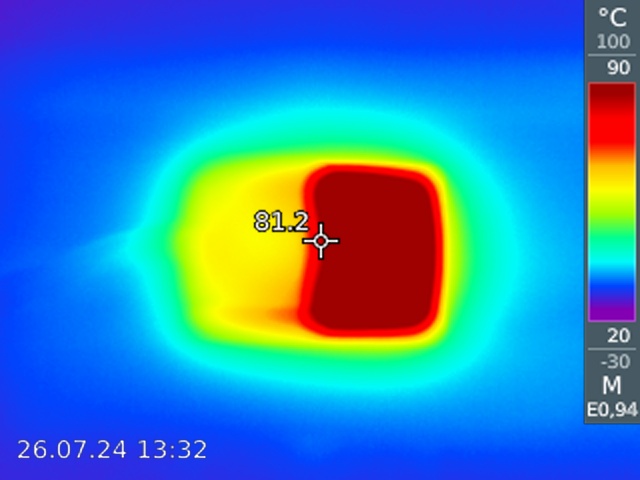

Supplement: S2 Figs — (ZIP) [file pone.0338325.s002.zip › image series/6. 3mm/TR004476.JPG]

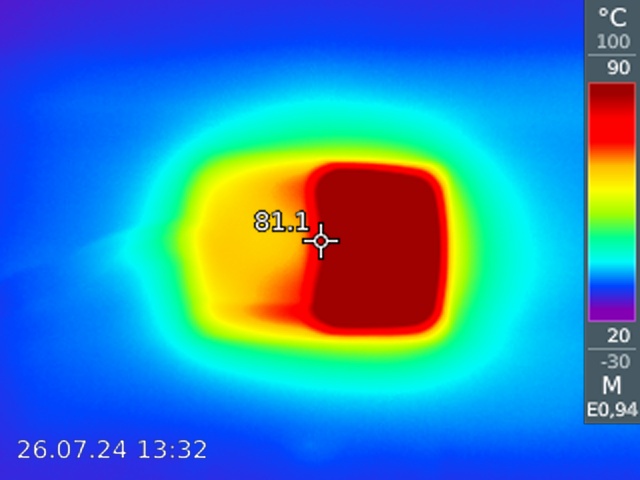

Supplement: S2 Figs — (ZIP) [file pone.0338325.s002.zip › image series/6. 3mm/TR004477.JPG]

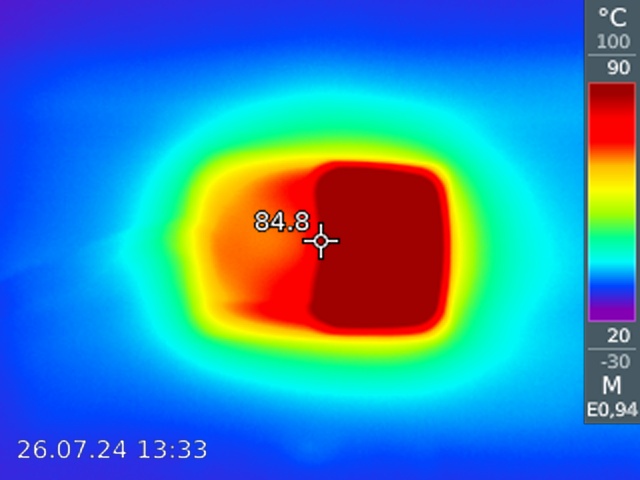

Supplement: S2 Figs — (ZIP) [file pone.0338325.s002.zip › image series/6. 3mm/TR004478.JPG]

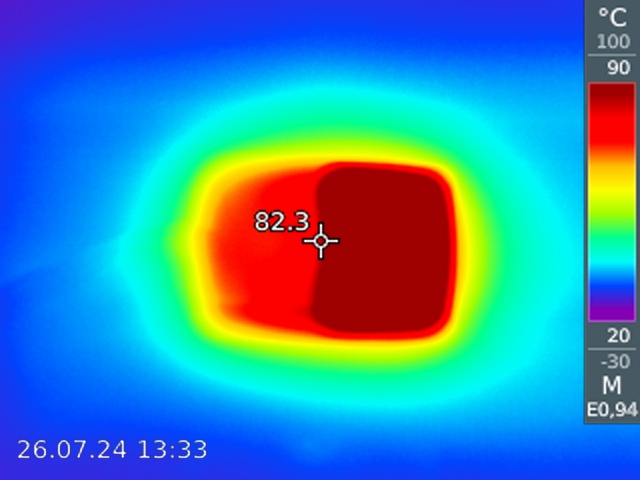

Supplement: S2 Figs — (ZIP) [file pone.0338325.s002.zip › image series/6. 3mm/TR004479.JPG]

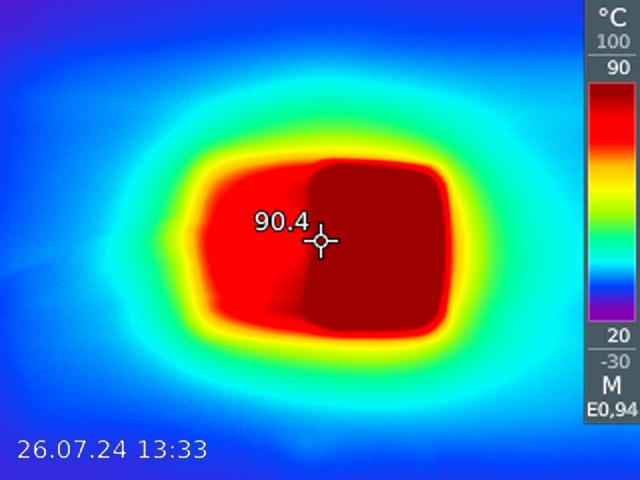

Supplement: S2 Figs — (ZIP) [file pone.0338325.s002.zip › image series/6. 3mm/TR004480.JPG]

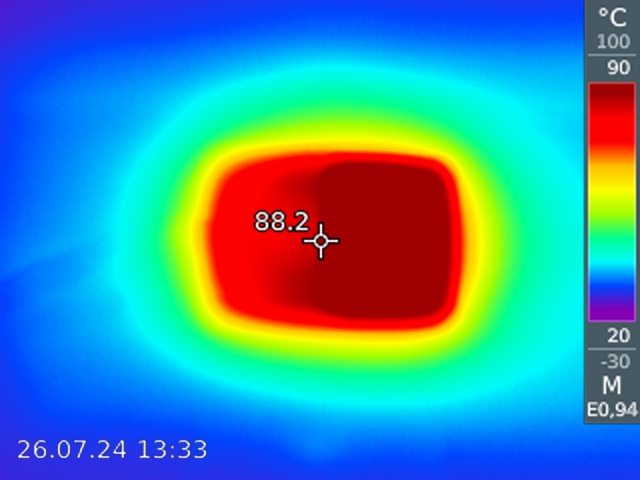

Supplement: S2 Figs — (ZIP) [file pone.0338325.s002.zip › image series/6. 3mm/TR004481.JPG]
