# Supplementary material for: Thermal insulation of poly(methyl methacrylate) bone cement and hydroxyapatite coatings under induction heating of metal implants
Source: PLoS One. 2025 Dec 11;20(12):e0338325. doi: 10.1371/journal.pone.0338325 (PMC12698007; doi:10.1371/journal.pone.0338325)
Supplement: Attachment — Submitted filename: Response to Reviewers.docx [file pone.0338325.s004.docx]

## Journal Requirements

## Response:

Thank you for letting us know where our manuscript deviated from the Plos One requirements. We have amended the sections indicated by you; please let us know if any additional changes are necessary.

## Reviewer 1

### Comment 1:

The introduction only briefly addresses, and in some aspects overlooks, several critical factors influencing the mechanical properties of bone cements. These include the specific characteristics of the mixing process, the presence of contaminants that can naturally occur in the surgical field—such as blood, bone debris, or saline solutions—as well as even minor deviations in the proportions of components in binary materials. Each of these factors can substantially alter the cement’s microstructure, polymerization kinetics, and long-term performance in vivo, ultimately affecting its clinical effectiveness. A more comprehensive discussion of these aspects is warranted, supported by relevant literature highlighting the effects of mixing protocols, intraoperative contamination, and compositional imbalances on the mechanical integrity and reliability of bone cements (DOI: 10.3390/ma12233963; 10.3390/ma12193073; 10.3390/ma15165577; 10.3390/ma15062197).

### Response 1:

Dear reviewer, thank you for your feedback and for providing these interesting references for us to read. We would like to point out that our study concerns the thermal properties of PMMA bone cement and HA coatings in the context of non-contact induction heating (NCIH). The mechanical properties of the cement pre and post NCIH are not within the scope of the current investigation, but have been studied in the past by us (<https://doi.org/10.12688/f1000research.148225.2>).

### Comment 2:

The Ti6Al4V–PMMA and Ti6Al4V–HA constructs used in this study have simplified geometry and reduced mass compared to actual implants, which may influence the real temperature distribution. In future work, it is recommended to extend the study to models with geometries more closely resembling clinical implants. For the current manuscript, the discussion should be supplemented with literature data comparing laboratory conditions with in vivo scenarios.

### Response 2:

Thank you for pointing this out. We have already addressed the expected impact of different implant size and geometry compared to our model in the “Limitations & strengths” section of the manuscript. We have copied the relevant section here for your convenience:

“The time required for heating could be different for real implants, due to their greater mass and complex geometry compared to our model. This could increase the thermal dose measured on the implant-tissue interface. However, a previous study showed that similar timespans were needed to heat real implants to 60°C (10-60s) compared to our model (20-70 seconds). Therefore, it is unlikely that the difference in implant size leads to differences in thermal dose delivered to surrounding tissue.”

### Comment 3:

The PAA gel phantoms do not account for dynamic cooling by blood flow and physiological fluids. The authors are encouraged to include numerical simulations or literature-based models addressing the perfusion effect in tissues to better estimate the safety of NCIH under clinical conditions.

### Response 3:

Thank you for pointing this out. We have already addressed the expected differences between PAA gel and living tissues in the “Limitations & strengths” section of the manuscript. The gel phantoms used in our study are created using a procedure from the international standard ASTM F2182-19. The gel phantoms created using this procedure have a thermal capacity and thermal conductivity constant similar to human tissues.

Nevertheless, there is reason to believe that living tissue will have increased heat sink capacity compared to the gel phantoms used in this study (see studies that modelled the effect of blood flow in hyperthermia situations: <https://doi.org/10.1016/0360-3016(79)90725-9> and <https://doi.org/10.1088/2057-1976/ad0398>). As a consequence, our model can be seen as a ‘worst case scenario’, and the real situation will lead to an even greater cooling effect. Since the possibility of using NCIH has been demonstrated even under these unfavourable conditions, we would argue that it is unnecessary to perform even more in vitro studies, and that the next step would be to test NCIH in ex vivo or in vivo scenarios.

### Comment 4:

The results are presented mainly as averages from two measurements, without an analysis of statistical significance. It would be advisable to perform statistical analysis (e.g., ANOVA or non-parametric tests) to assess differences between sample configurations and temperatures; if this is not feasible, the limitation should be explicitly described in the manuscript.

### Response 4:

Thank you for your feedback. We are of the opinion that statistical analysis of data based on two replicate measurements will not reveal any insights not apparent from qualitative consideration of the data. Our research aim was to establish the relationship between implant modality and the potential temperatures that could be safely employed during NCIH treatment. Since statistical significance does not imply clinical significance and a clear trend in the thermal doses received at the implant surface based on implant modality could be seen, we were satisfied with our current data. There is a growing resistance against the (wrongful) use of p-values (e.g. “Scientists rise up against statistical significance”, <https://doi.org/10.1038/d41586-019-00857-9>) and statistical significance does not equate to clinical significance. Achieving a statistically significant estimation of the exact temperature threshold where the CEM43 of our model exceeds 16 is not clinically relevant. Finally, we would like to point out that the CEM43 threshold is exceeded by multiple orders of magnitude in implant modalities considered ‘unsafe’ in our study. Repeating the experiments simply to be able to perform statistical tests on the data will not lead to better clinical outcome for patients.

### Comment 5:

Although threshold temperatures for PMMA and HA are mentioned, the study does not include post-heating assessments of, for example, changes in mechanical properties or coating adhesion. Future studies should incorporate mechanical and microscopic testing after heating cycles.

### Response 5:

We have already performed a study on the mechanical strength of these materials post induction heating. Our publication is available in open access at <https://doi.org/10.12688/f1000research.148225.2>.

### Comment 6:

The discussion of biological responses, including osteointegration and the effects of cement on surrounding bone tissue, requires further development. This should be complemented by an examination of factors—such as the incorporation of solid admixtures (e.g., ceramics, glassy carbon)—that have the potential to enhance both the mechanical performance and osteointegration of bone cements. Such modifications can influence not only the cement’s load-bearing capacity but also its bioactivity, promoting stronger and more durable bonding at the bone–cement interface. Detailed insights into these effects can be found in the series of studies Effect of Various Admixtures on Selected Mechanical Properties of Medium Viscosity Bone Cements: Part 1, Part 2, and Part 3, which should be reviewed and appropriately cited to complete the reference list.

### Response 6:

Dear reviewer, thank you for pointing out these interesting aspects of bone cement chemistry. As we pointed out in Response 1, our study focused on studying the thermal properties of implant modalities that are currently commonly used in the clinic, in the context of infection treatment using NCIH, rather than mechanical or biological properties of these materials.

### Comment 7:

Although synergy with antibiotics is mentioned, the manuscript lacks data on the effectiveness of NCIH in reducing biofilm at different temperatures. The authors are encouraged to expand the discussion to address the mechanisms by which sub-thermal heat doses affect biofilm and the potential implications for antibiotic resistance.

### Response 7:

The effect of NCIH on biofilms in vitro as well as the synergistic effect of NCIH and antibiotics has already been investigated in depth in several studies, please see the following references:

- <https://doi.org/10.1302/2046-3758.119.BJR-2022-0010.R1>
- <https://doi.org/10.1186/s13018-024-04785-x>
- <https://doi.org/10.1302/2046-3758.145.BJR-2024-0341.R1>
- <https://doi.org/10.1302/2046-3758.94.BJR-2019-0274.R1>

### Comment 8:

The figures and tables are numerous but not always easy to interpret (e.g., small axis labels, lack of standardized color schemes). It would be beneficial to standardize figure formatting, increase the readability of labels, and apply a consistent color scheme throughout the manuscript.

### Response 8:

Thank you for your comment. Our colour scheme is as follows: for diagrams of our experimental setup, we use grey for metal parts, green for PMMA bone cement or HA coatings, light blue for PAA gel and beige for bone tissue. For temperature measurements with thermocouple, we use green for the top surface of the PMMA or HA, and red for the control side. For CEM43 values in Figure 16 specifically, we use blue and orange to specify the groups with and without gel, specifically. Please, let us know which specific changes we can make to improve our figures.

## Reviewer 2

### Comment 1:

Firstly, the pictured application is difficult to envision. The schematic of figure 1 is somehow idealized, the situation in the operating theatre is heterogenous and an exposition of the whole transition from the femoral head to the stem is difficult due to the massive musculature which is present. It is not clear how an induction coil would be placed to reach the desired parts of the implant successfully and adequately.

### Response 1:

Dear reviewer, thank you for your comments. Figure 1 of our manuscript is indeed idealised for clarity. In particular, the induction coil pictured in Figure 1 is deliberately oversized for clarity. In reality, an induction device designed for this purpose would be much smaller and designed to fit over the femoral head. Please see this publication, which features a device in Fig. 5 with a similar size and appearance: <https://doi.org/10.1302/2046-3758.121.BJR-2022-0216.R1>

We hope we have satisfied your curiosity with this answer. Please let us know if we can provide additional information.

### Comment 2:

Furthermore, it seems difficult to understand that the overlying tissue layer – here in the model represented by the PAA gel – is called “heat sink”. The layer which acts in this set up as heat sink is the actual tissue which will be damaged by absorbing parts of the heat.

### Response 2:

Thank you for pointing out this apparent contradiction. Implying the function of the gel is simply a heat sink was not our intention; naturally, the gel itself represents the very tissue that must be protected from thermal damage. To avoid confusion, we have removed most references of the words ‘heat sink’ from the manuscript, except where it is specifically relevant to discuss this property.

We measured the thermal doses at the implant-tissue interface (i.e. T_top_), which is the site of the highest thermal dose administered to living tissue. The gel phantom, in this scenario, represents the surrounding tissue and can indeed act as a heat sink that decreases the temperature of the implant-tissue interface. Since the temperature of the deeper tissue will never be higher than at interface (as evidenced by the thermographs in S2), we can be sure that if the CEM43 at the interface is below the safe upper limit, this will also be true for the surrounding tissue, i.e. the gel.

### Comment 3:

The temperature range (< 30°C) given at which the experiments were conducted is rather broad. Since the surrounding temperature is a crucial factor for the experiments so the exact temperature for each experimental run should be stated to allow for a correct evaluation.

### Response 3:

Thank you for pointing this out. 30°C was selected as the minimum temperature to start the next experiment (naturally, the model has to cool down between each experiment). Therefore, most experiments were started at 25- 30°C. However, the first experiment of any given day for one model started at room temperature (20-25°C). We have added an additional supplementary file S3 containing the starting temperature of each experiment.

### Comment 4:

Lastly, it is not clear why the measurements of each group are only performed twice. While in some groups the double measurement led to relatively similar values, there were more obvious differences in others. Although the presence of these variation in the values are discussed in the limitations & strengths section, the actual values of the single measurements are nowhere stated. They must be included in the paper. Repetition of the measurement will clarify the reason for the variation and if the reason given in the limitations & strengths section is correct. It is strongly suggested to increase the number of measurements or give a reasonable explanation for not doing so.

### Response 4:

Thank you for your comment. Although the differences between the duplicate measurements could be reviewed qualitatively in Figs. 10-15 and S1, the CEM43 values of individual measurements were not shared. We have added an additional Supplementary file S3, containing an expanded version of Table 1 with individual measurements included.

The most major discrepancy between two replicates can be seen for 90°C in the model with a PMMA layer of 1mm and no PAA gel (CEM43 2E09 vs 5E07). However, both of these values are clearly higher than the upper CEM43 safe limit of 1.6E1. This was true for every measurement: In all cases, both replicates were either above or below the safe limit. Therefore, any variation in the measured CEM43 did not affect the outcomes of the study. We have added this observation to the Discussion section.

### Comment 5:

Section 2.1.3 Final sample preparation: Please include some short but clear information about the used spray-paint. The given reference (15) is a paper which also gives no more detailed information but instead also references another literature which is not freely available.

### Response 5:

Thank you for your comment. The spray paint we used was matte black spray paint from the brand SPECTRUM. Without the spray-paint, the thermal emissivity of the different biomaterials (Ti6Al4V, HA, PMMA) will show apparent differences in temperature due to differences in the thermal emissivity of the materials. Adding the paint equalises the emissivity. In Fig. 2 of the reference of our manuscript (<https://doi.org/10.1302/2046-3758.711.BJR-2018-0080.R1>) shows a side-by-side comparison of two implants with and without spray-paint, clearly exhibiting its necessity.

We have expanded Section 2.1.3. of the manuscript to clarify our use of the spray-paint.

### Comment 6:

Section 2.2.2 Quantitative measurement: The exact total number of the experiments for each group has to be included.

### Response 6:

We apologise for any unclarity. The number of duplicate experiments was already stated at the end of section 2.2.2.: “All experiments were conducted in duplicate and average values were reported. All experiments were carried out with and without gel phantoms.”. Since a total of 10 model systems were subjected to heating to 3 distinct temperatures in duplicate, the total number of experiments was 60. We have added this number to Section 2.2.2. to avoid confusion.

### Comment 7:

Fig. 10 – 15: In the Figure captions 90°C are stated while in the figures themselves 70°C is stated. Please revise correctly. Additionally, the thermographic pictures have to be explained better, maybe by adding one schematic, especially for the upper part: what means the different height of the coloured graph? For readers not familiar with this technique this is not self-explainable.

### Response 7:

Thank you for pointing out our mistake, we have changed the Figure captions accordingly.

Additionally, we agree that more clarification is needed for the cross-section profiles of the thermographs. We have added a short description to the Results section and changed the Figure captions accordingly.

### Comment 8:

Table 1: For Ttop max the standard deviation should be stated. Furthermore, it is confusing that for the control implants (Cneg) also the term “T top” is used. Shouldn´t it be “T bottom”? Please clarify and consider adding a respective foot note to the table.

### Response 8:

We chose to refer to the thermocouple that represents the implant-tissue interface (i.e., the only interface that has direct contact with living tissue) as ‘T_top_’. In the case of the negative controls and the HA coated samples, that means ‘T_bottom_’ is not present. The method suggested by you is possible; however, this would mean that the most biologically relevant interface is not named consistently across all samples, and it would further complicate Table 1. We apologise for any confusion this may have caused.

### Comment 9:

Outlook: While alternative strategies for preventing bacterial growth are of great importance, the mentioned strategy here has to be explained further. How should the coil be applied in a closed-skin/prothesis-in-situ situation be placed? What time-points should be chosen for heating? How do you want to decide when to heat the implant for prevention purposes?

### Response 9:

We appreciate your interest in the clinical applications of NCIH. Certainly, a different device is needed for non-invasive heating of the implant, compared to invasive heating. The induction coil, in this case, would need to fit around the patient’s body, or remain to one side. Different coil modalities can be feasible for this purpose, depending on the joint that must be heated. As an example, in a previous study (see Fig. 1 of <https://doi.org/10.12688/f1000research.148225.2>), we used a simple solenoid coil to heat a Ti6Al4V cylinder; this type of coil could be used to heat most types of joint such as knees and ankles, with the possible exception of a hip. As another example, this study (<https://doi.org/10.1302/2046-3758.711.BJR-2018-0080.R1>) used a different, double-coil design where the direction of the magnetic field was perpendicular to the implant. Much research has been performed on optimising induction coil design for targeted and localised heating, see <https://doi.org/10.1016/j.apenergy.2023.121535> and <https://doi.org/10.1088/0031-9155/27/1/001>.

To answer your second question, we envision that the choice to start preventative treatment can be based on risk factors for infection, both pre-op (immunocompromising factors such as diabetes, cancer, or proton pump inhibitor medication) and post-op (persistent wound leakage, signs of inflammation). It should be noted that the temperatures needed to prevent infection are likely lower than those needed to destroy existing biofilms; as such, preventative NCIH can be considered a low-risk intervention that is suitable for most patients with little associated risk.

In terms of preventative NCIH strategies, this could be continual heating at relatively low temperatures, lasting for multiple hours (i.e. a ‘simulated fever’ condition), or short bursts of higher temperatures, similar to an infection treatment protocol. More in vitro research is needed to find the optimal strategy in terms of temperature and heating time to maximally reduce the chance of infection.

We have incorporated these points into the Outlook section of our manuscript.
