# Supplementary material for: Thermal insulation of poly(methyl methacrylate) bone cement and hydroxyapatite coatings under induction heating of metal implants
Source: PLoS One. 2025 Dec 11;20(12):e0338325. doi: 10.1371/journal.pone.0338325 (PMC12698007; doi:10.1371/journal.pone.0338325)
Supplement: Attachment — Submitted filename: Response_to_Reviewers_auresp_2.docx [file pone.0338325.s005.docx]

## Reviewer 1

### Comment 1:

The authors corrected the paper according to the guidelines presented in the previous report, thank you for your contribution to improving the manuscript. In its present form, the paper can be further processed and accepted for publication.

### Response 1:

Dear reviewer, thank you for reviewing our manuscript. We are happy to hear that you are satisfied with the changes we made.

## Reviewer 2

### Comment 2:

Figure captions 10 – 15: Changing “thermal profile” to “thermal cross-section” may improve the understanding, however adding some information IN the figure itself is strongly recommended; at least number/letters should be included for the different figure parts (e.g. A, B, C). As I understand, the cross-section is given for the black line in the thermograph? If so, please add this information to the figure caption.

Furthermore, there is still no explanation about the “height-profile” of the cross-section. Is it just “the higher the temperature, the higher the image of cross-section”? What additional, valuable information does the cross-section offer to the reader which cannot be found in the thermograph? Please explain the reason to include the cross-section.

### Response 2:

Dear reviewer, thank you for your continued scrutiny of our manuscript. Your efforts are appreciated. Indeed, the black line in the top-down thermograph indicates the position of the cross-section view.

We have changed figures 10-15 in the following ways, based on your feedback:

1. We have added letters to indicate each figure part as you suggested, and changed the Figure captions accordingly.
2. We have changed the Figure captions to explain the relation between the top-down thermograph and the thermal cross-section

The y-axis of the thermal cross-sections indicates the temperature indeed. The thermal cross-sections do not convey additional information, compared to the top-down thermographs; however, they do allow the reader to see more clearly the transitions from one part of the implant models to another, since it is generally easier to distinguish changes in shape rather than changes in hue, especially for people who are colour-blind. Since the cross-sections do not take up any space that could otherwise be utilised for something else, we have elected to keep them as-is. Please let us know if you disagree.

### Comment 3:

I understand the explanation for using Ttop consistently for all sample as representative to the implant – tissue – interface. However, as it is crucial for the reader to understand the terminology unmistakably and the method section for this terminology does not explain it well enough the reader relies mainly on Figure 9. Therefore, Figure 9 has to be supplemented by a second scheme for the control samples, so that it is total clear which terminology applies for which part of which sample.

### Response 3:

We agree with your suggestion to expand Figure 9 to provide more clarity on the nomenclature of the thermocouples. We have changed Figure 9 to represent all 3 configurations (bone cement, HA coating and control), and changed the figure caption accordingly.

### Comment 4:

### There is a typo in the discussion (section 4.1.1, second paragraph):

### “...for 3.5 minutes[14,15].[18] While…”

### Please check for the use of space bar and style of given references.

### Response 4:

Thank you for your diligence. We have amended the error that you pointed out, as well as other formatting errors.
